# Structural and Biophysical Characterization of Aminobenzo[*d*]isothiazole 1,1-Dioxide SOS1 Ligands

**DOI:** 10.1021/acsmedchemlett.6c00300

**Published:** 2026-07-03

**Authors:** Thiago Moreira Pereira, Atilio Reyes Romero, Marleen van der Laan, Guan Zhirui, Rick Oerlemans, Matthew Groves, Matteo Mori, Fiorella Meneghetti, Alexander Dömling

**Affiliations:** † Regional Centre of Advanced Technologies and Materials, Czech Advanced Technology and Research Institute (CATRIN), 74795 Palacký University Olomouc, Šlechtitelů 27, Olomouc, 783 71, Czech Republic; ‡ Institute of Molecular and Translational Medicine, Faculty of Medicine and Dentistry, Palacký University and University Hospital Olomouc, Olomouc, 779 00, Czech Republic; § Faculty of Science and Engineering, Chemical and Pharmaceutical Biology - Groningen Research Institute of Pharmacy, 225118 University of Groningen, Groningen, 9713 AV, Netherlands; ∥ Department of Pharmaceutical Sciences DISFARM, University of Milan, Milano, 20133, Italy

**Keywords:** aminobenzo[*d*]isothiazole 1, 1-dioxide, SOS1, KRAS, biophysics, protein−protein interactions, cancer

## Abstract

SOS1 is a guanine nucleotide exchange factor that promotes KRAS activation by catalyzing GDP release and GTP loading, making SOS1-mediated nucleotide exchange an attractive therapeutic target in KRAS-driven cancers. Herein, we report the synthesis, biophysical characterization, and structural analysis of aminobenzo­[*d*]­isothiazole 1,1-dioxide SOS1 ligands. Compound **6f** engaged SOS1 with a K_D_ of 570 nM by microscale thermophoresis, supported by surface plasmon resonance. X-ray crystal structures of SOS1 bound to compounds **6b** and **6f** refined previous binding hypotheses regarding pocket engagement. Rather than being dominated by sulfone-mediated contacts, SOS1 recognition is primarily driven by hydrophobic and aromatic packing within the canonical pocket, a conserved ligand NH hydrogen bond to Asn879, and π-π stacking with Tyr884, recapitulating key features of BI-3406 binding. Comparative analysis further delineates vector requirements for productive engagement of KRAS-facing regions, providing a structure-based framework for future optimization of aminobenzo­[*d*]­isothiazole 1,1-dioxide SOS1 ligands.

## Graphical abstract

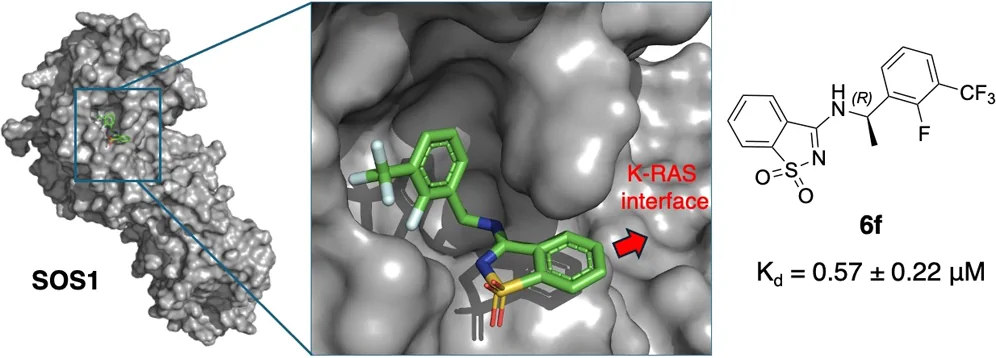

Son of sevenless (SOS) proteins are guanine nucleotide exchange factors (GEFs) that promote KRAS activation by catalyzing guanosine diphosphate (GDP)-to-guanosine triphosphate (GTP) exchange, thereby converting KRAS from its inactive GDP-bound state into its active GTP-bound form.^1^ Active KRAS engages downstream signaling pathways including the RAF-MEK-ERK mitogen-activated protein kinase (MAPK) cascade and the phosphoinositide 3-kinase (PI3K)-AKT-mTOR signaling axis, which regulate proliferation, survival, and resistance to apoptosis.^2–4^ Oncogenic KRAS mutations such as G12C, G12D, G13D, and Q61R impair GTP hydrolysis, resulting in persistent KRAS activation and sustained oncogenic signaling in multiple cancers.^5,6^ Consequently, pharmacological modulation of SOS1-mediated nucleotide exchange has emerged as an attractive strategy to suppress KRAS-driven tumors.^7–11^ SOS1 activates KRAS through its catalytic CDC25 domain and is further stimulated by an allosteric Ras-binding site that amplifies KRAS-GTP production through a positive-feedback mechanism. Because KRAS activation requires formation of the ordered SOS1-KRAS complex, the SOS1-KRAS protein–protein interface represents a pharmacologically actionable target. The discovery of small-molecule ligands such as BI-3406 demonstrated that this interface is ligandable and that disruption of SOS1-mediated nucleotide exchange can shift KRAS toward its inactive GDP-bound state.^12,13^ Following the discovery of SOS1-KRAS interface ligands such as BI-3406, medicinal chemistry efforts have increasingly focused on improving the physicochemical properties of SOS1-targeting scaffolds. Recently, amino benzo­[*d*]­isothiazole 1,1-dioxide derivatives were identified by AstraZeneca as a promising follow-up chemotype displaying improved solubility and lower LogD values relative to benzothiadiazine analogues.^14^ However, structural information describing how this scaffold engages SOS1 and whether pocket binding translates into functional modulation of nucleotide exchange remains limited. To address this question, we synthesized a focused series of amino benzo­[*d*]­isothiazole 1,1-dioxide derivatives (**6a**–**g**) (Scheme S1) and evaluated them using orthogonal biophysical assays including thermal shift assay (TSA), microscale thermophoresis (MST), surface plasmon resonance (SPR), X-ray crystallography, and nucleotide exchange assays (NEA). Single-crystal structures of selected ligands were also obtained to support unambiguous structural assignment and provide conformational context for this chemotype; these data are reported in the Supporting Information and deposited with the CCDC.

Initial TSA screening identified racemic **6e** and the (*R*)-enantiomer **6f** as weak but reproducible SOS1 binders, producing Δ*T*_m_ shifts of +1.0 and +2.0 °C in line with previously reported measurements,^13^ respectively (Figure 1A), whereas the remaining analogues showed no detectable stabilization. Given the limited sensitivity of the initial TSA screening and the modest magnitude of the observed melting temperatures, SOS1 binding was further investigated using MST as an orthogonal solution-phase assay.

**Figure 1:**
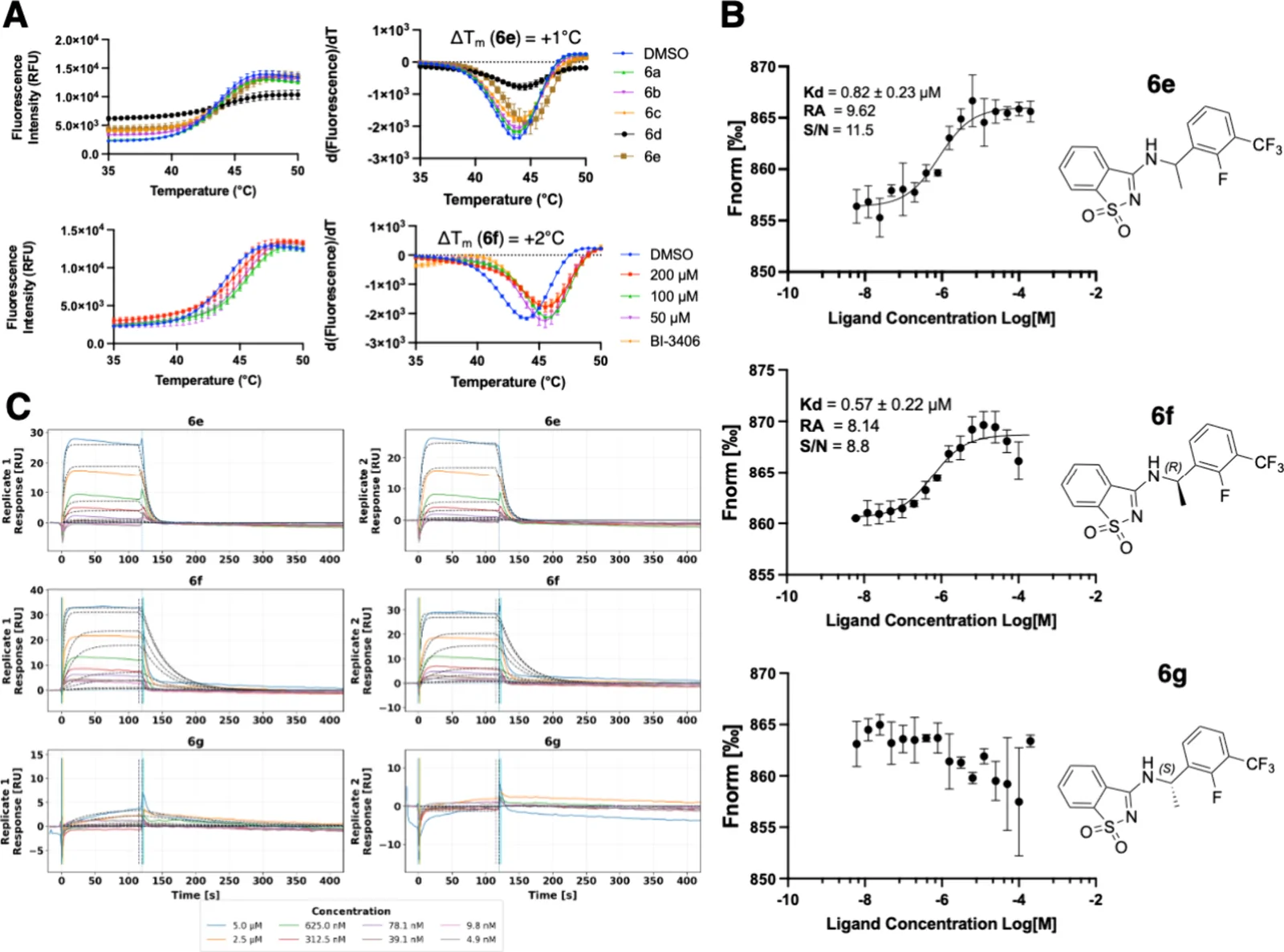
Biophysical characterization of aminobenzo­[*d*]­isothiazole 1,1-dioxide derivatives **6e**, **6f**, and **6g** against SOS1. (**A**) Primary screening performed by thermal shift assay displayed as raw fluorescence traces (left) and first-derivative plots (right) of SOS1 with DMSO, BI-3406, and compounds **6a**–**6e** (top), and dose-dependent profiling of **6f** (bottom). Raw fluorescence traces and first-derivative plots are shown in the first and second columns, respectively, with the corresponding Δ*T*_m_ values. The temperature range was restricted to 35–50 °C to improve visualization of the melting transitions. (**B**) Compound **6g** showed no detectable binding, while **6f** displayed an apparent hook effect at the highest ligand concentrations. (**C**) SPR sensorgrams. Experimental traces (colored) overlaid with global kinetic fits using a 1:1 Langmuir binding model (black lines). MST and TSA data are represented as mean ± SD of three technical replicates.

MST measurements confirmed binding of **6e** and **6f** in a dose-dependent manner with dissociation constants (K_d_) of 0.82 ± 0.23 and 0.57 ± 0.22 μM, respectively, while the (*S*)-enantiomer **6g** showed no measurable interaction (Figure 1B). SPR experiments independently supported concentration-dependent binding of **6e** and **6f** to SOS1 (Figure 1C and Table S1). Consistently with the MST results, compounds **6a**–**d** did not show appreciable binding in the orthogonal validation experiments, irrespective of the aromatic substituent introduced at the methyl-bearing stereocenter, namely, phenyl (**6a**), naphthyl (**6b**), 4-(benzyloxy)­phenyl (**6c**), and 2-chloro-4-(4-chlorophenoxy) phenyl (**6d**) groups (Figure S1A). Similarly, the SPR data and low apparent *R*_max_ values did not provide convincing kinetic profiles compatible with a 1:1 Langmuir binding model for compounds **6a**–**c**. The only exception was **6d**, whose sensorgrams, recorded over a concentration range from 35 μM to 34 nM, could be fitted by global kinetic analysis, indicating binding to SOS1 (Figure S1B).

To elucidate the structural basis of binding, X-ray crystal structures of SOS1 in complex with **6f** (Figures 2A and S2A) and **6b** (Figures 2B and S2B) were determined at 1.54 Å resolution (Table S2). Both compounds engaged the canonical SOS1 pocket through a hydrophobic region defined by Phe890, Lys898, and Leu901, retained π-related contacts with Tyr884 and His905, and displayed conserved polar interactions involving Asn879. Specifically, the ortho-fluorine atom of **6f** mediates high binding cooperativity by engaging in interactions with Glu902 and Leu901. The chlorine substituent in **6d** may provide additional halogen-associated contacts compared to the hydrogen-substituted analogue **6c**. However, its larger van der Waals radius may limit optimal complementarity within the SOS1 pocket, contributing to the weak overall binding behavior observed by SPR.

**Figure 2:**
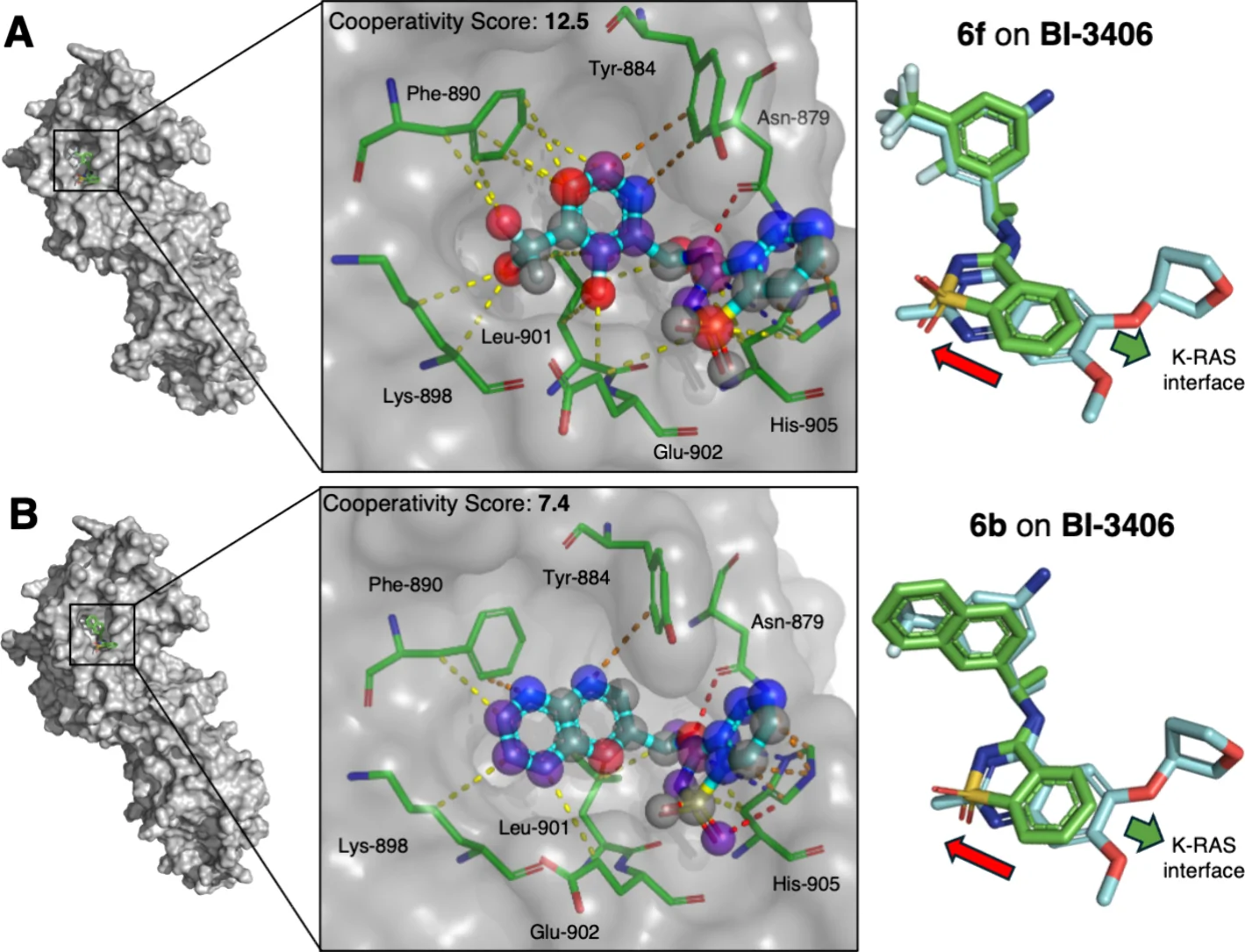
Crystal structures of SOS1 bound to compounds (**A**) **6f** (PDB ID: 30JO) and (**B**) **6b (**PDB ID: 30JN), respectively. Each panel shows, from left to right, the ligand position within the SOS1 binding pocket, the local binding environment with selected interacting residues, and the structural overlay with the reference inhibitor BI-3406 (cyan sticks, PDB ID: 6SCM). Both structures show the lack of steric hindrance toward the KRAS interface and the retraction of the aminobenzo­[*d*]­isothiazole 1,1-dioxide scaffold compared to the isoquinoline one. SOS1 is shown as a gray molecular surface while ligands and selected residues are shown as green sticks. Protein–ligand interactions are color-coded as follows: hydrogen bonds (red), van der Waals contacts (yellow), π-π interactions (orange), cation-π interactions (blue). The green arrow indicates the KRAS-facing projection vector occupied by BI-3406, while the red arrow highlights the recessed position of the aminobenzo­[*d*]­isothiazole 1,1-dioxide scaffold, which limits extension toward the SOS1–KRAS interface.

Structural overlay with BI-3406 further highlighted the conserved role of the trifluoromethyl group as an anchoring element within the SOS1 pocket. On the other hand, the naphthyl ring anchoring **6b** to SOS1 contributed 3.0 out of a Scorpion score of 7.4 (41%), whereas the corresponding fluorinated aromatic region of **6f** contributed 7.8 out of 12.5 (62%). The reduced binding cooperativity combined with poor geometric complementarity with pocket residues may explain the lack of measurable affinity in solution via MST and the absence of convincing binding in SPR. At the same time, the overlay revealed that ring contraction of the heteroaromatic scaffold from a six- to a five-membered system, as observed for **6f** and **6b**, leads to a more-recessed binding pose. Together, the structural and biophysical data support the ability of the aminobenzo­[*d*]­isothiazole 1,1-dioxide scaffold to engage the SOS1 pocket. At the same time, the recessed binding geometry imposed by scaffold contraction may restrict extension toward the KRAS interface, offering a structural rationale for the apparent disconnect between pocket binding affinity and functional inhibition.

To address this hypothesis, the functional consequences of SOS1 binding were evaluated using BODIPY-GTP loading and BODIPY-GDP dissociation assays at 10 μM compound concentration. Consistently with the crystallographic analysis, only the reference compound BI-3406 produced robust inhibition of SOS1-mediated nucleotide exchange in both assay formats (Figure 3A-D). In the GTP-loading assay, BI-3406 reduced the exchange rate by 98.7 ± 2.6% relative to DMSO (adjusted P = 0.004), whereas compounds **6a**–**e** produced only modest effects that did not reach statistical significance. Similarly, in the GDP-dissociation assay, BI-3406 decreased the exchange rate by 46.9 ± 7.1% (adjusted P = 0.007), while the amino benzo­[*d*]­isothiazole 1,1-dioxide derivatives showed weaker and more variable responses. These findings suggest that the recessed binding geometry of this scaffold limits productive projection toward the KRAS interface despite measurable SOS1 pocket engagement. Complete quantitative values are summarized in Table 1.

**Figure 3:**
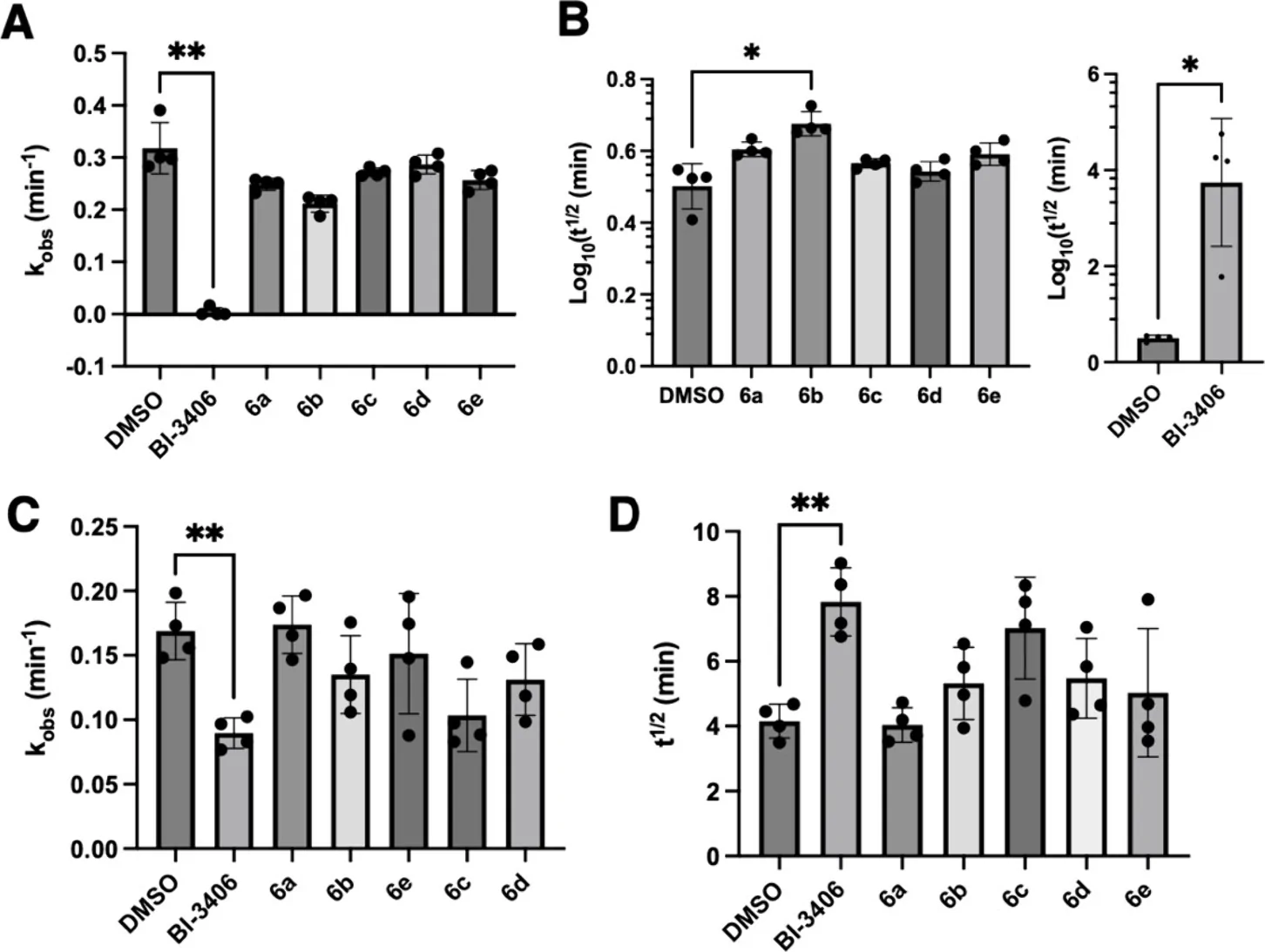
Bar plots summarizing SOS1-mediated nucleotide exchange, shown as the observed rate constant (*k*_obs_) and half-time (*t*_1/2_) for the BODIPY-GTP association (**A, B**) and as *k*_obs_ and half-life for the BODIPY-GDP dissociation assays (**C, D**). Bars represent mean ± SD (*n* = 4). Statistical significance is indicated as * *P* < 0.05; ** *P* < 0.01; *** *P* < 0.001.

**Table 1:**
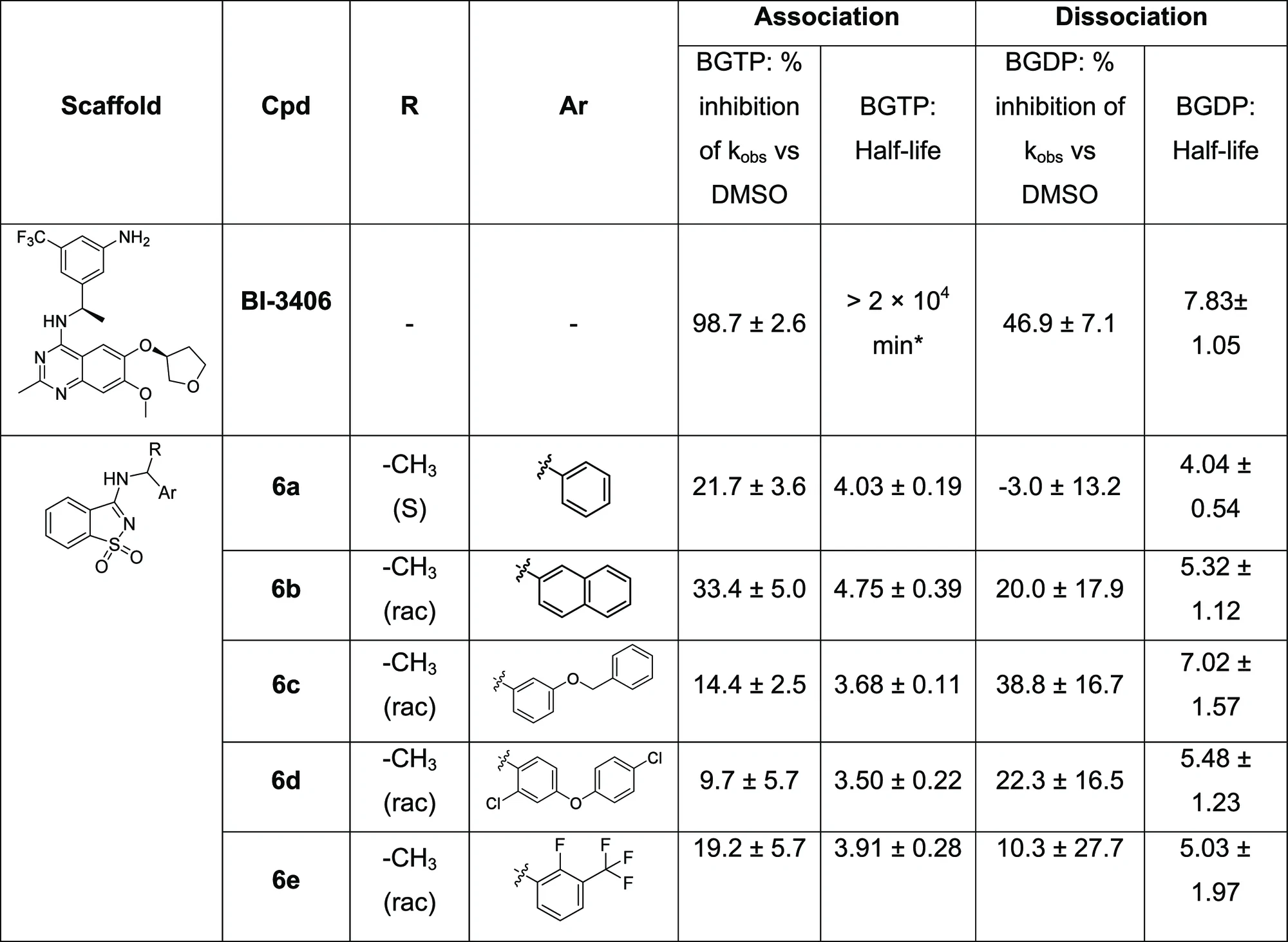
Summary of BGTP and BGDP Nucleotide Exchange Parameters for BI-3406 and **6a**–**e**

In this study, we provide a concise structural and biophysical characterization of aminobenzo­[*d*]­isothiazole 1,1-dioxide derivatives engaging the SOS1 pocket, including crystal structures of representative members of this scaffold in complex with SOS1. These findings extend the work of Bonomo and co-workers,^14^ who identified this chemotype as a promising follow-up to benzothiadiazine dioxides based on improved physicochemical properties and docking-derived binding hypotheses. Specifically, our crystallographic data refine previous binding hypotheses regarding pocket engagement. Whereas prior modeling suggested a major contribution from sulfone-mediated interactions, our structural analysis reveals that SOS1 recognition is primarily driven by hydrophobic and aromatic packing interactions, alongside a conserved hydrogen bond between the ligand NH group and Asn879 and π stacking interaction Tyr884, closely mimicking the binding mode of BI-3406. Next, we define the strict stereochemical requirements governing SOS1 pocket recognition. An enantiomeric comparison between **6f** and **6g** indicates that binding is highly dependent on the orientation of the chiral center: the (*R*)-enantiomer (**6f**) displays a robust binding profile with the methyl group filling a small cavity and orients the phenyl group for an edge-to-face interaction with Phe890. In addition, we demonstrate that measurable SOS1 pocket binding does not automatically translate into robust functional modulation of nucleotide exchange. Despite submicromolar binding for selected analogues, these aminobenzo­[*d*]­isothiazole 1,1-dioxide derivatives produced only modest effects in BODIPY-GTP loading and BODIPY-GDP dissociation assays compared with BI-3406. Structural comparison suggests that this functional disconnect is not caused by a loss of canonical pocket recognition, as the ligands retain key contacts and engage Asn879. Instead, both the contraction of the heteroaromatic scaffold from a six- to a five-membered ring system and the lack of a functional group mediating steric contact position the core in a more recessed binding pose, thereby limiting productive projection toward the KRAS-facing interface. Vectors addressing the Tyr884/Arg73 region, as exemplified by quinazoline^15^ and phthalazine such as MRTX0902,^16^ appear critical for translating SOS1 binding into PPI disruption. In our series, Tyr884-associated contacts may contribute to pocket engagement, but the recessed binding pose likely limits efficient perturbation of the SOS1-KRAS interface. Taken together, these findings do not exclude the aminobenzo­[*d*]­isothiazole 1,1-dioxide scaffold as a viable SOS1 ligand platform. Instead, they define a structural boundary condition for its future optimization: productive SOS1 inhibition will require preserving the favorable pocket interactions and physicochemical profile of this chemotype while redirecting substituent vectors toward the KRAS-facing interface. Future analogue design should therefore prioritize stereochemically defined ligands that maintain the Asn879-anchored binding mode while extending from vectors capable of perturbing the Tyr884/Arg73 interface region.

## Supplementary Material



## Data Availability

CCDC entries 2528438 (**6a**), 2528439 (**6c**), 2527969 (**3b**), 2528324 (**3d**), 2528325 (**1d**), 2528702 (**6d**), 2528437 (**6b**) contain all supplementary crystallographic data. These data can be obtained free of charge from the Cambridge Crystallographic Data Centre via https://www.ccdc.cam.ac.uk/structures. The crystallographic coordinates of the SOS1 complexes with **6f** and **6b** have been deposited in the Protein Data Bank under accession codes **30JO** and **30JN**, respectively.

## Notes

Synthetic procedures; compound characterization data; ^1^H and ^13^C NMR, HPLC, and HRMS spectra; chiral SFC separation details; crystallographic methods, refinement statistics, and ligand electron-density maps; biophysical assay protocols and supplementary TSA, MST, SPR, and nucleotide exchange data; computational ligand binding analysis (PDF)
