# Supplementary material for: Structural and Biophysical Characterization of Aminobenzo[d]isothiazole 1,1-Dioxide SOS1 Ligands
Source: ACS Med Chem Lett. 2026 Jul 3;17(8):1895–900. doi: 10.1021/acsmedchemlett.6c00300 (PMC13488084; doi:10.1021/acsmedchemlett.6c00300)
Supplement: Supplementary file 1 [file ml6c00300_si_001.pdf]

Supporting Information
For
Structural and Biophysical Characterization of Aminobenzo[*d*]isothiazole 1,1-Dioxide SOS1 Ligands

Thiago Moreira Pereira,[1,2, #] Atilio Reyes Romero,[1,2, #], Marleen van der Laan,[3] Guan Zhirui,[1,2] Rick Oerlemans,[3] Matthew Groves,[3] Matteo Mori,[4] Fiorella Meneghetti,[4] and Alexander Dömling[1,2,*]

[1] Regional Centre of Advanced Technologies and Materials, Czech Advanced Technology and Research Institute (CATRIN), Palacký University Olomouc, Šlechtitelů 27, Olomouc, 783 71, Czech Republic.

[2] Institute of Molecular and Translational Medicine, Faculty of Medicine and Dentistry, Palacký University and University Hospital Olomouc, Olomouc, Czech Republic.

[3] Faculty of Science and Engineering, Chemical and Pharmaceutical Biology - Groningen Research Institute of Pharmacy, University of Groningen, Groningen, Netherlands.

[4] Department of Pharmaceutical Sciences DISFARM, University of Milan, Milano, Italy

[*] Corresponding author: alexander.domling@upol.cz (A.D.)

# These authors contributed equally to this work

TABLE OF CONTENTS

SUPPLEMENTARY FIGURES … S5
Figure S1. Biophysical profiling of compounds **6a–6d** against SOS1 … S5
Figure S2. Electron-density map for SOS1-bound compounds **6f** and **6b** … S6
Figure S3. Thermal-ellipsoid diagram and crystal packing of **6a** … S31
Figure S4. Hirshfeld surface and energy-framework analysis of **6a** … S32
Figure S5. Thermal-ellipsoid diagram and crystal packing of **6c** … S33
Figure S6. Hirshfeld surface and energy-framework analysis of **6c** … S34
Figure S7. Energy-framework analysis of **6a** with CrystalExplorer … S47
Figure S8. Energy-framework analysis of **6c** with CrystalExplorer … S48

SUPPLEMENTARY TABLES … S7
Table S1. SPR global kinetic fitting parameters for SOS1 binders … S7
Table S2. Crystallographic statistics for **6f** and **6b** bound to SOS1 … S8
Table S3. Crystal data and structure refinement statistics for **6a** and **6c** … S36
Table S4. Crystal data and structure refinement for **3b** … S38
Table S5. Crystal data and structure refinement for **3d** … S40
Table S6. Crystal data and structure refinement for **1d** … S42
Table S7. Crystal data and structure refinement for **6d** … S44
Table S8. Crystal data and structure refinement for **6b** … S46
Table S9. Hirshfeld surface analysis of **6a** … S47
Table S10. Hirshfeld surface analysis of **6c** … S48

MATERIAL AND METHODS … S9

1. **CHEMISTRY** … S9
1.1 Chemistry General Experimental … S9
1.2 NMR Spectroscopy … S9
1.3 High-Resolution Mass Spectroscopy … S9
1.4 HPLC Analytical Methods … S9
1.5 Synthesis of the Compounds … S9
Scheme S1. Synthesis of amino benzo[d]isothiazole 1,1-dioxide series (**6a–e**) … S10
1.5.1 Synthesis of Formamides (**3b–e**) … S10
1.5.2 Synthesis of 1-(naphthalen-2-yl)ethan-1-amine (**1b**) … S10
1.5.3 Synthesis of 1-(3-(benzyloxy)phenyl)ethan-1-amine (**1c**) … S11
1.5.4 Synthesis of 1-(2-chloro-4-(4-chlorophenoxy)phenyl)ethan-1-amine hydrochloride (**1d**) … S11

1.5.5 Synthesis of 1-(2-fluoro-3-(trifluoromethyl)phenyl)ethan-1-amine (**1e**) S11
1.5.6 Synthesis of 3-chlorobenzo[d]isothiazole 1,1-dioxide (**5**) S11
1.5.7 Synthesis of Benzosulfimide derivatives (**6a–e**) S12
1.5.8 Synthesis of (S)-3-((1-phenylethyl)amino)benzo[d]isothiazole 1,1-dioxide (**6a**) S12
1.5.9 Synthesis of 3-((1-(naphthalen-2-yl)ethyl)amino)benzo[d]isothiazole
1,1-dioxide (**6b**) S12
1.5.10 Synthesis of 3-((1-(3-(benzyloxy)phenyl)ethyl)amino)benzo[d]isothiazole
1,1-dioxide (**6c**) S12
1.5.11 Synthesis of 3-((1-(2-chloro-4-(4-chlorophenoxy)phenyl)ethyl)amino)
benzo[d]isothiazole 1,1-dioxide (**6d**) S13
1.5.12 Synthesis of 3-((1-(2-fluoro-3-(trifluoromethyl)phenyl)ethyl)amino)
benzo[d]isothiazole 1,1-dioxide (**6e**) S13
1.5.13 Enantiomeric separation of **6e** S13

1.6 ^{1}H NMR and ^{13}C NMR Spectra of Compounds S15
1.6.1 ^{1}H NMR (500 MHz) spectra of compound **6a** in DMSO-d6 .... S15
1.6.2 ^{13}C NMR (125 MHz) spectra of compound **6a** in DMSO-d6 .... S16
1.6.3 HPLC chromatogram and HRMS spectrum of compound **6a** S17
1.6.4 ^{1}H NMR (500 MHz) spectra of compound **6b** in DMSO-d6 .... S18
1.6.5 ^{13}C NMR (125 MHz) spectra of compound **6b** in DMSO-d6 .... S19
1.6.6 HPLC chromatogram and HRMS spectrum of compound **6b** S19
1.6.7 ^{1}H NMR (500 MHz) spectra of compound **6c** in DMSO-d6 .... S21
1.6.8 ^{13}C NMR (125 MHz) spectra of compound **6c** in DMSO-d6 .... S22
1.6.9 HPLC chromatogram and HRMS spectrum of compound **6c** S23
1.6.10 ^{1}H NMR (500 MHz) spectra of compound **6d** in DMSO-d6 .... S24
1.6.11 ^{13}C NMR (125 MHz) spectra of compound **6d** in DMSO-d6 .... S25
1.6.12 HPLC chromatogram and HRMS spectrum of compound **6d** S26
1.6.13 ^{1}H NMR (500 MHz) and ^{13}C NMR (125 MHz) spectra of compound **6e** in DMSO-d6 .. S27
1.6.14 ^{13}C NMR (125 MHz) spectra of compound **6e** in DMSO-d6 .... S28
1.6.15 HPLC chromatogram and HRMS spectrum of compound **6e** S28

2. **COMPOUND STRUCTURAL ELUCIDATION BY X-RAY CRYSTALLOGRAPHY** S29
2.1 Compound Crystallography Experimental Procedures S34
2.2 N-(1-(Naphthalen-2-yl)ethyl)formamide (**3b**) S37
2.3 (R)-N-(1-(2-Chloro-4-(4-chlorophenoxy)phenyl)ethyl)formamide (**3d**) S39
2.4 (S)-1-(2-Chloro-4-(4-chlorophenoxy)phenyl)ethan-1-amine hydrochloride (**1d**) S41
2.5 3-((1-(2-Chloro-4-(4-chlorophenoxy)phenyl)ethyl)amino)benzo[d]isothiazole

1,1-dioxide (**6d**) S43
2.6 3-((1-(Naphthalen-2-yl)ethyl)amino)benzo[d]isothiazole 1,1-dioxide (**6b**) S45
2.7 (S)-3-((1-Phenylethyl)amino)benzo[d]isothiazole 1,1-dioxide (**6a**) S47
2.8 3-((1-(3-(Benzyloxy)phenyl)ethyl)amino)benzo[d]isothiazole 1,1-dioxide (**6c**) S48

3. **BIOLOGY** S49
3.1 Protein Expression and Purification S49
3.2 SOS1 (co-)crystallization S49
3.3 Thermal Shift Assay S50
3.4 Microscale Thermophoresis S50
3.5 Loading of KRAS with Nucleotides S51
3.6 Nucleotide Exchange Assay S51
3.7 Surface Plasmon Resonance S52

4. **COMPUTATIONAL CHEMISTRY** S53
4.1 Evaluation of Ligand Binding Cooperativity of the Co-crystal Structures S53

5. **REFERENCES** S53

SUPPLEMENTARY FIGURES

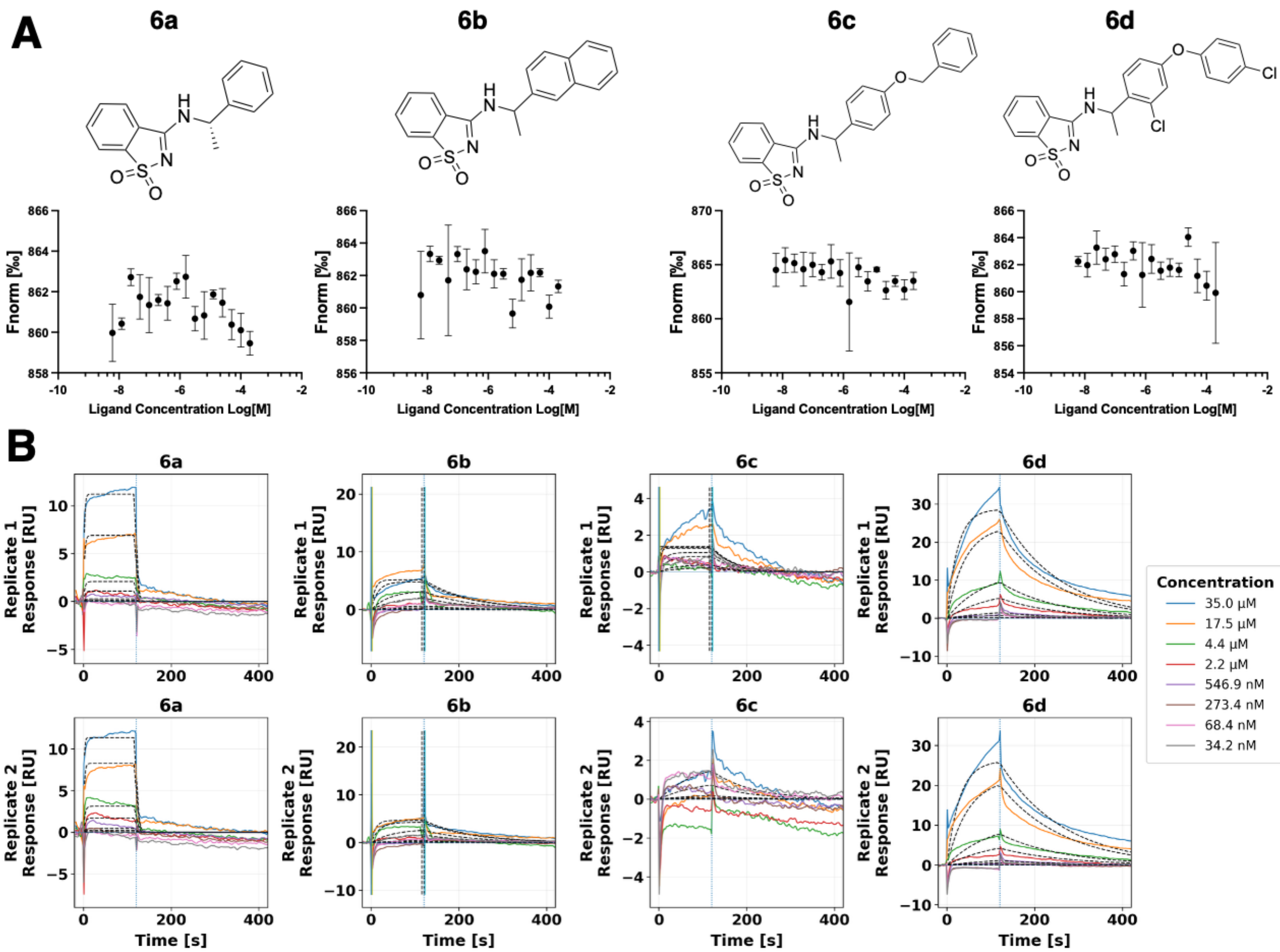

Figure S1. Biophysical profiling of compounds **6a**-**6d** against SOS1. (**A**) MST binding curves with corresponding chemical structures plotted as normalized fluorescence (F_{norm}) versus ligand concentration; none of the compounds showed detectable binding due to the low signal to noise ratio. Data are represented as mean ± SD of three technical replicates. (**B**) SPR sensorgrams; experimental traces (colored) fitted to a 1:1 Langmuir model (black dotted lines).

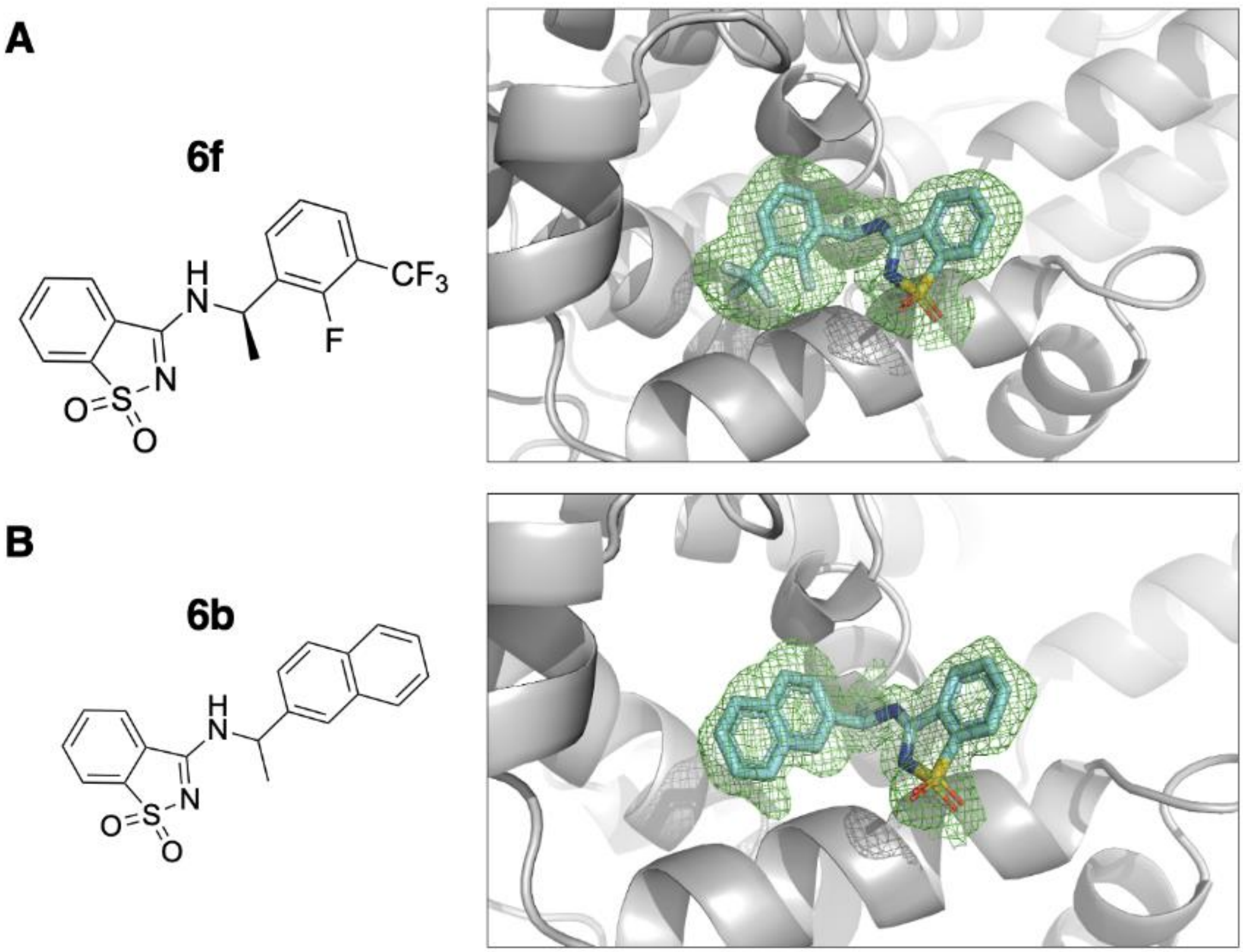

Figure S2. Electron-density map for SOS1-bound compounds **6f** (**A**) and **6b** (**B**). Chemical structures are shown on the left; crystallographic binding poses in the SOS1 pocket are shown on the right. Ligands are displayed as sticks and overlaid with the corresponding mFo–DFc electron-density maps (green mesh, contoured at +3.0 σ, carved within 1.6 Å around the ligand atoms).

SUPPLEMENTARY TABLES

Table S1. SPR global kinetic fitting parameters for SOS1 binders. Values are reported as mean ± SD of n= 2.

Compound	Global fit quality	k_on (M^{-1} s^{-1})	k_off (s^{-1})	KD (μM)	Rmax (RU)	χ^2/Rmax (%)	Off-rate χ^2/Rmax (%)
**6a**	Pass/Pass	(8.81 ± 4.93) $\times 10^3$	0.2832 ± 0.0357	39.49 ± 26.17	24.02 ± 8.22	2.3 ± 1.6	3.0 ± 2.0
**6b**	Care/Care	(2.88 ± 0.44) $\times 10^3$	0.0093 ± 0.0001	3.27 ± 0.46	5.37 ± 0.35	10.6 ± 0.9	6.2 ± 0.0
**6c**	Care/Pass	(5.91 ± 8.36) $\times 10^4$	0.0158 ± 0.0065	(1.03 ± 1.46) $\times 10^5$	(5.83 ± 8.25) $\times 10^3$	10.1 ± 14.2	6.2 ± 8.8
**6d**	Pass/Pass	(8.88 ± 1.19) $\times 10^2$	0.0073 ± 0.0005	8.21 ± 0.52	33.88 ± 1.74	4.0 ± 0.0	2.8 ± 0.3
**6e**	Pass/Pass	(3.51 ± 1.27) $\times 10^4$	0.1213 ± 0.0133	3.62 ± 0.93	43.57 ± 3.14	2.1 ± 0.5	1.6 ± 0.8
**6f**	Care/Care	(1.03 ± 0.04) $\times 10^5$	0.0310 ± 0.0019	0.300 ± 0.006	32.53 ± 3.25	45.2 ± 5.9	27.3 ± 7.0
**6g**	Care/Poor	(1.53 ± 1.70) $\times 10^4$	(1.72 ± 2.44) $\times 10^4$	(6.30 ± 8.91) $\times 10^5$	(-1.62 ± 2.29) $\times 10^5$	3.3 ± 4.7	2.7 ± 3.8

Table S2. Crystallographic statistics for **6f** and **6b** bound to SOS1 catalytic pocket**.**

**Data collection**	**SOS1 and 6f (PDB ID: 30JO)**	**SOS1 and 6b (PDB ID: 30JN)**
Space group	P 21 21 21	P 21 21 21
Cell dimensions		
a, b, c (Å)	41.228, 85.489, 173.868	41.407, 86.386, 173.235
α, β, γ (º)	90.000, 90.000, 90.000	90.000, 90.000, 90.000
Resolution	1.54 (8.44)	1.54 (8.43)
Completeness (%)	100.0 (99.2)	100.0 (99.5)
R_{merge}	0.040 (0.026)	0.049 (0.026)
<I / σ (I)>	24.2 (1.0)	20.8 (1.0)
Redundancy	13.2 (80.6)	13.3 (11.2)
CC1/2	1.000 (0.999)	1.000 (1.000)
**Refinement**		
Resolution	1.54 - 40.15	1.54 - 43.23
No. reflections	91973	93036
R_{work}/R_{free}	0.166/0.203	0.186/0.218
No. atoms		
Proteins	7545	7539
Ligand/Ion	37	40
Water	336	376
B-Factors (Å)		
Proteins	41	36
Ligand/Ion	34.6	38.3
Water	47.9	42.1
RMS deviations		
Bond lengths (Å)	0.0116	0.0113
Bond angles (º)	1.705	1.852

MATERIAL AND METHODS

1. CHEMISTRY

1.1 Chemistry General Experimental

Reagents were available from commercial suppliers (BLDpharm, Fluorochem, Sigma Aldrich, ABCR, Acros and AK Scientific) and used without any purification. Thin layer chromatography was performed on precoated silica gel 60 F254 plates (Merck, Darmstadt). Flash chromatography was performed on a Buchi Pure Essential Chromatography System Normal-phase Silica Flash Columns (Silica Gel 60 Å, 230 - 400 mesh). BI-3406 was kindly provided by Boehringer Ingelheim via its open innovation platform opnMe (https://www.opnme.com/).

1.2 NMR Spectroscopy

Nuclear magnetic resonance spectra were recorded on a JEOL ECX500 spectrometer (^{1}H NMR (500 MHz), ^{13}C NMR (125 MHz)). Chemical shifts for ^{1}H NMR were reported in ppm relative to TMS (δ = 0.00 ppm) or the corresponding solvent peak (DMSO-*d*$_6$ δ = 2.50 ppm) and coupling constants were reported in Hertz (Hz). The following abbreviations were used for spin multiplicity: s = singlet, bs = broad singlet, d = doublet, dd = doublet of doublets, t = triplet, q = quartet, and m = multiplet. Chemical shifts for ^{13}C NMR were reported in ppm relative to the solvent peak (DMSO-*d*$_6$ δ = 39.52 ppm).

1.3 High-Resolution Mass Spectroscopy

HRMS analysis was performed using an LC-MS Orbitrap Elite high-resolution mass spectrometer with electrospray ionization (Dionex Ultimate 3000, Thermo Exactive plus, MA, USA)

1.4 HPLC Analytical Methods

Liquid chromatography analysis was used to determine compound purity using a Waters UPLC-UV-MS Acquity instrument. Enantiomeric separation was performed using Waters ACQUITY UPC^2 equipped with a photodiode array (PDA) detector and a QDa detector.

1.5 Synthesis of the Compounds

Aminobenzo[*d*]isothiazole 1,1-dioxide derivatives (**6a-e**) were synthesized by three-step protocol. As shown in **Scheme S1**, the first step was the synthesis of amines (**1b-e**) from commercially available ketones under Leuckart-Wallach conditions.[1] Ketones (**2b-e**) were refluxed in formamide-formic acid medium to afford the corresponding formamides (**3b-e**), which were subsequently hydrolyzed under acidic or basic medium leading to substituted alkylamines (**1b-e**) (78 to 91% yield).
The second step was the synthesis of 3-chlorobenzo[*d*]isothiazole 1,1-dioxide (**5**) from saccharin (**4**) via chlorination in $POCl_3$ at reflux for 18h, affording **5** in 57% yield. Finally, in the third step, amino

benzo[*d*]isothiazole 1,1-dioxide (**6a-f**) were obtained in 60-73% yield via nucleophilic substitution of the imidoyl chloride (**5**) with (S)-1-phenylethan-1-amine (**1a**) or alkylamines (**1b-e**) in THF at reflux (**Scheme S1**). Amino benzo[*d*]isothiazole 1,1-dioxide derivatives **6f-g** were obtained by enantiomeric separation of **6e** using chiral supercritical fluid chromatography (SFC) with CO_2 and EtOH as the mobile phase.

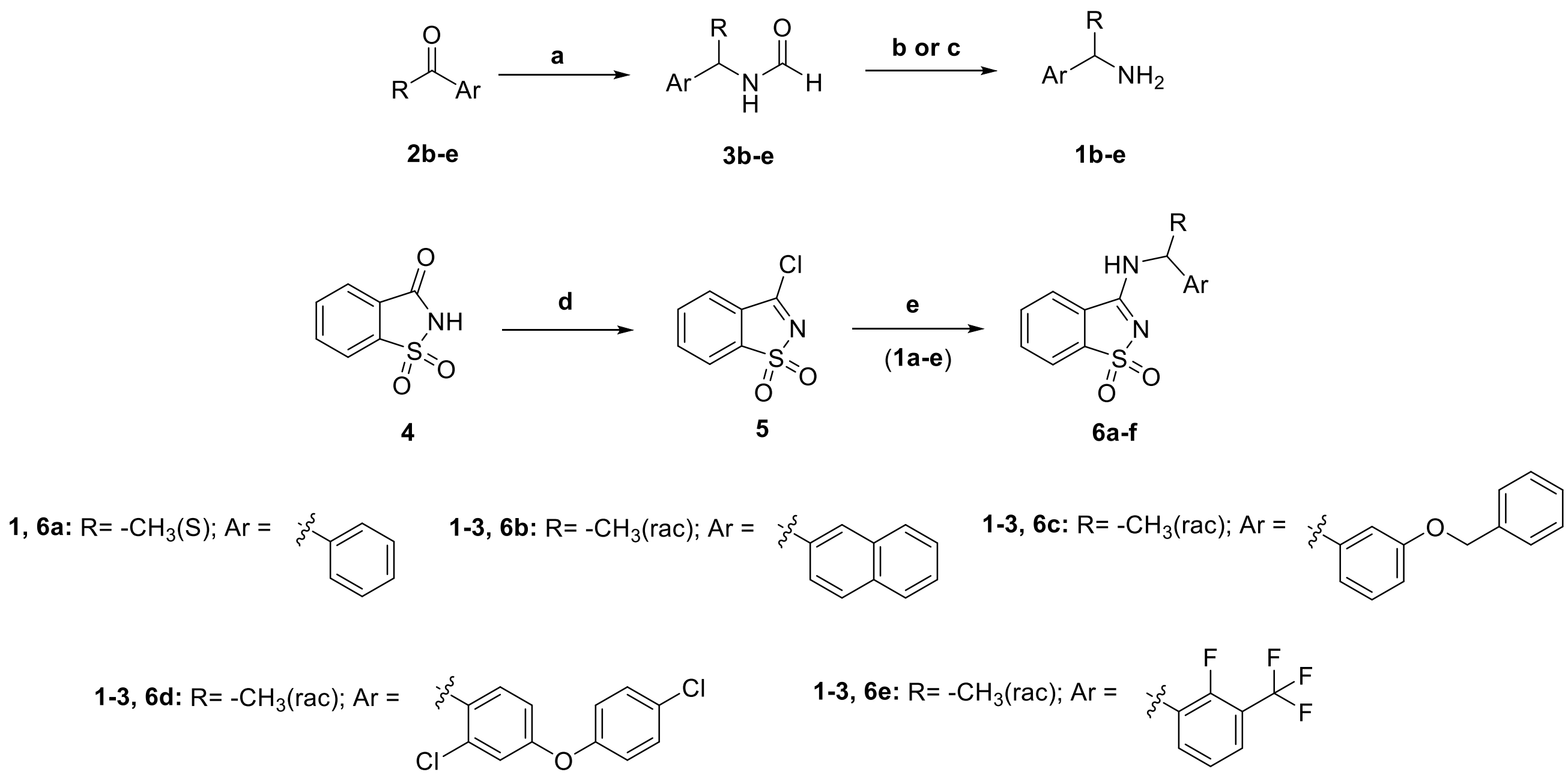

Scheme S1. Synthesis of amino benzo[*d*]isothiazole 1,1-dioxide series (**6a-e**). Reagents and Conditions: (a) HCOOH, $HCONH_2$, 180 °C, 5 h. (b) HCl 4M, H_2O, 100 °C, 14-20 h, yield 65 to 83 %. (c) NaOH, H_2O: EtOH: 1,4-dioxane (1: 1: 1), 100 °C, 6 h, yield 90 %. (d) $POCl_3$, 140 °C, 18 h, yield 57 %. (e) THF, reflux, 16h, yield 60 to 73%.

1.5.1 Synthesis of Formamides (3b-e)

A borosilicate reaction vessel equipped with magnetic stir bar was charged with ketone (**2b-e**) (3.00 g), formamide ($HCONH_2$) (15 mL) and formic acid (HCOOH) (6 mL). The resulting mixture was stirred at 180 °C for 5 hours and the progress of the reaction was monitored by TLC. The reaction mixture was cooled to room temperature, dissolved with H_2O (50 mL) and extracted with DCM (50 mL x 3). The combined organic phase was washed with H_2O (2 x 50 mL) and brine (50 mL), dried over Na_2SO_4 and concentrated under reduced pressure to afford the title product without further purification.

1.5.2 Synthesis of 1-(naphthalen-2-yl)ethan-1-amine (1b)

The corresponding formamide 3b (1 equiv., 0.25 g, 1.25 mmol) was added in a borosilicate reaction vessel equipped with a Teflon stir bar and dissolved in 4 mL of H_2O: 1,4-dioxane (1: 1) followed by the

addition of HCl(aq), 35% (w/w) (1 mL). The reaction mixture was stirred at 100 °C for 14h and the progress of the reaction was monitored by LC-MS. After completion, the crude mixture was cooled to room temperature, diluted with H_2O (10 mL) and extracted with DCM (10 mL x 3). The aqueous phase was then basified until pH > 12 and extracted with ethyl acetate (10 mL x 3). The combined organic phase was then dried over anhydrous Na_2SO_4 and concentrated under reduced pressure to afford the title product (0.167 g, 0.975 mmol, 78%) without further purification.

1.5.3 Synthesis of 1-(3-(benzyloxy)phenyl)ethan-1-amine (1c)

The corresponding formamide **3c** (1 equiv., 0.5 g, 1.96 mmol) was added in a borosilicate reaction vessel equipped with a Teflon stir bar and dissolved in 4 mL of H_2O: EtOH: 1,4-dioxane (1: 1: 1). NaOH (3 equiv.) was then added, and the reaction mixture was stirred at 100 °C for 6h. The progress of the reaction was monitored by LC-MS. The crude mixture was cooled to room temperature, diluted with H_2O (10 mL) and acidified to pH 2. The resulting solution was extracted with DCM (10 mL x 3). The aqueous phase was then basified to pH > 12 and extracted with ethyl acetate (10 mL x 3). The combined organic phase was then dried over anhydrous Na_2SO_4 and concentrated under reduced pressure to afford the title product without further purification.

1.5.4 Synthesis of 1-(2-chloro-4-(4-chlorophenoxy)phenyl)ethan-1-amine hydrochloride (1d)

The corresponding formamide **3d** (1 equiv., 0.5 g, 1.6 mmol) was added in a borosilicate reaction vessel equipped with a Teflon stir bar and dissolved in 8 mL of H_2O: 1,4-dioxane (1: 1) followed by the addition of HCl(aq), 35% (w/w) (2 mL). The reaction mixture was stirred at 100 °C for 20h and the progress of the reaction was monitored by LC-MS. The crude mixture was cooled to room temperature and then diluted with H_2O (20 mL) and DCM (20 mL). The precipitated formed was then filtered off to obtain the title product as white solid (0.2 g, 0.7 mmol, 43%) without further purification.

1.5.5 Synthesis of 1-(2-fluoro-3-(trifluoromethyl)phenyl)ethan-1-amine (1e)

The corresponding formamide **3e** (1 equiv., 0.5 g, 0.21 mmol) was added in a borosilicate reaction vessel equipped with a Teflon stir bar and dissolved in 8 mL of H_2O: 1,4-dioxane (1: 1) followed by the addition of HCl(aq), 35% (w/w) (2 mL). The reaction mixture was stirred at 100 °C for 14h and the progress of the reaction was monitored by LC-MS. After completion, the crude mixture was cooled to room temperature, diluted with H_2O (10 mL) and extracted with DCM (10 mL x 3). The aqueous phase was then basified until pH > 12 and extracted with ethyl acetate (10 mL x 3). The combined organic phase was then dried over anhydrous Na_2SO_4 and concentrated under reduced pressure to afford the title product (0.285 g, 1.38 mmol, 65%) without further purification.

1.5.6 Synthesis of 3-chlorobenzo[*d*]isothiazole 1,1-dioxide (**5**)

A 150 mL round-bottom flask equipped with a Teflon stir bar was charged with saccharin (**4**) (1 equiv., 2 g, 10.9 mmol) and $POCl_3$ (15 equiv., 25.1 g, 15.3 mL, 164 mmol). A condenser was coupled and the reaction mixture was then placed in a preheated sand bath at 140 °C until complete conversion of the starting material was detected by TLC. The reaction mixture was cooled, concentrated under reduced pressure, diluted with ethyl acetate (50 mL) and then washed with H_2O (50 mL), aqueous saturated $NaHCO_3$ (50 mL) and brine (50 mL). The organic layer was then dried over anhydrous Na_2SO_4 and concentrated to give the desired product **5** (1.25 g, 6.2 mmol, 57%).

1.5.7 Synthesis of aminobenzo[*d*]isothiazole 1,1-dioxide derivatives (**6a-e**)

General procedure: A borosilicate reaction vessel equipped with a Teflon stir bar was charged with 3-chlorobenzo[*d*]isothiazole 1,1-dioxide (**5**) (1 equiv., 0.1 g, 0.5 mmol), THF (2 mL) and amine (2 equiv., 1 mmol). The reaction mixture was heated at reflux overnight and then cooled to room temperature. Crushed ice was added to the mixture and the solid formed was filtered off and dried under air exposure. The dry solid was then sonicated with ethyl acetate and filtered off to give desired product.

1.5.8 Synthesis of (S)-3-((1-phenylethyl)amino)benzo[*d*]isothiazole 1,1-dioxide (**6a**)

This product was obtained as white solid using general procedure, 0.5 mmol scale, 103.1 mg, 73% yield. ^1H NMR (500 MHz, DMSO-d_6): δ 1.60-1.62 (d, *J* = 7.0 Hz, 3H), 5.25 (m, 1H), 7.29 (t, *J*= 7.30 Hz, 1H), 7.38 (t, *J*= 7.65 Hz, 2H), 7.43-7.45 (m, 2H), 7.81-7.86 (m, 2H), 7.96-7.97 (m, 1H), 8.33-8.35 (m, 1H), 9.66 (s, 1H). ^{13}C NMR (125 MHz, DMSO-d_6): δ 21.6, 52.3, 121.2, 123.2, 126.2, 127.3, 127.7, 128.5, 133, 133.5, 142.2, 142.5, 158.5. HRMS (ESI) m/z calculated for $C_{15}H_{14}N_2O_2S$ $[M+H]^+$: 287.0848; found $[M+H]^+$: 287.0845.

1.5.9 Synthesis of 3-((1-(naphthalen-2-yl)ethyl)amino)benzo[*d*]isothiazole 1,1-dioxide (**6b**)

This product was obtained as white solid using general procedure, 0.5 mmol scale, 118.3 mg, 71% yield. ^1H NMR (500 MHz, DMSO-d_6): δ 1.66-1.68 (d, *J* = 6.9 Hz, 3H), 5.39 (q, *J* = 6.9 Hz, 1H), 7.47-7.53 (m, 2H), 7.63 (dd, *J*= 1.7 Hz, *J*= 8.5 Hz, 1H), 7.77-7.82 (m, 2H), 7.89-7.95 (m, 5H), 8.29-8.31 (d, *J*= 7.0 Hz, 1H). ^{13}C NMR (125 MHz, DMSO-d_6): δ 22, 53, 120.9, 123.2, 124.4, 125, 125.8, 126.2, 127.5, 127.8, 128, 132.2, 132.7, 132.9, 133, 142.4, 158. HRMS (ESI) m/z calculated for $C_{19}H_{16}N_2O_2S$ $[M+H]^+$: 337.1005; found $[M+H]^+$: 337.1003.

1.5.10 Synthesis of 3-((1-(3-(benzyloxy)phenyl)ethyl)amino)benzo[*d*]isothiazole 1,1-dioxide (**6c**)

This product was obtained as white solid using general procedure, 0.5 mmol scale, 115.9 mg, 60 % yield. ^1H NMR (500 MHz, DMSO-d_6): δ 1.59-1.60 (d, *J* = 7.0 Hz, 3H), 5.09 (s, 2H), 5.20-5.26 (m, 1H),

6.94 (dd, *J*= 2.3 Hz, *J*= 8 Hz, 1H), 7.01-7.02 (d, *J*= 7.7 Hz, 1H), 7.1 (m, 1H), 7.27-7.31 (m, 2H), 7.34-7.37 (m, 2H), 7.43-7.45 (m, 2H), 7.81-7.87 (m, 2H), 7.96-7.98 (m, 1H), 8.32-8.34 (m, 1H), 9.61-9.63 (d, *J*= 7.4 Hz, 1H). ^{13}C NMR (125 MHz, DMSO-d_6): δ 21.7, 52.2, 69.2,113, 113.3, 118.6, 121.2, 123.3, 127.7, 127.8, 127.8, 128.4, 129.6, 133, 133.5, 137, 142.1, 144.2, 158.5. HRMS (ESI) m/z calculated for $C_{22}H_{20}N_2O_3S$ [M+H]$^+$: 393.1267; found [M+H]$^+$: 393.1263.

1.5.11 Synthesis of 3-((1-(2-chloro-4-(4-chlorophenoxy)phenyl)ethyl)amino)benzo[*d*]isothiazole 1,1-dioxide (6d)

A borosilicate reaction vessel equipped with a Teflon stir bar was charged with 3-chlorobenzo[*d*]isothiazole 1,1-dioxide (**5**) (1 equiv., 0.1 g, 0.5 mmol), THF (2 mL), MeCN (2 mL), Et_3N (3 equiv., 1.5 mmol) and corresponding amine (1.3 equiv., 0.65 mmol). The reaction mixture was heated at reflux overnight and then cooled to room temperature. Crushed ice was added, and the resulting solid was collected by filtration and dried in air. The crude solid was sonicated with ethyl acetate, then filtered and dried. The resulting solid was recrystallized with EtOH:MeCN (1: 1) to give the desired product as white needles (67.4 mg, 0.5 mmol, 30% yield). ^{1}H NMR (500 MHz, DMSO-d_6): δ 1.58-1.60 (d, *J* = 7 Hz, 3H), 5.44-5.50 (m, 1H), 7.05 (dd, *J*= 2.5 Hz, *J*= 8.6 Hz, 1H),7.09-7.12 (m, 2H), 7.15 (d, *J*= 2.5 Hz, 1H), 7.44-7.47 (m, 2H), 7.57-7.59 (d, , *J*= 8.7 Hz, 1H), 7.82-7.88 (m, 2H), 7.96-7.98 (m, 1H), 8.34-8.36 (m, 1H), 9.70-9.72 (d, *J*= 6.6 Hz, 1H). ^{13}C NMR (125 MHz, DMSO-d_6): δ 20.5, 49.4, 117.6, 119.1, 121, 121.3, 123.3, 127.5, 128, 128.4, 130, 132.6, 133.1, 133.6, 135.2, 142, 154.7, 156.4, 158.5. HRMS (ESI) m/z calculated for $C_{21}H_{16}Cl_2N_2O_3S$ [M+H]$^+$: 447.0331; found [M+H]$^+$: 447.0348.

1.5.12 Synthesis of 3-((1-(2-fluoro-3-(trifluoromethyl)phenyl)ethyl)amino)benzo[*d*]isothiazole 1,1-dioxide (6e)

This product was obtained as white solid using general procedure, 0.5 mmol scale, 112.7 mg, 61% yield. ^{1}H NMR (500 MHz, DMSO-d_6): δ 1.64-1.65 (d, *J* = 7.0 Hz, 3H), 5.48 (m, 1H), 7.45 (t, *J*= 7.8 Hz, 1H), 7.72-7.75 (m, 1H), 7.82-7.88 (m, 3H), 7.96-7.98 (m, 1H), 8.33-8.35 (m, 1H), 9.77 (s, 1H). ^{13}C NMR (125 MHz, DMSO-d_6): δ 20.5, 46.6, 121.3, 123.4, 125.2, 126.5, 127.4, 131.3, 131.4, 132.6, 133.2, 133.7, 142, 158.7. HRMS (ESI) m/z calculated for $C_{16}H_{12}F_4N_2O_2S$ [M+H]$^+$: 373.0628; found [M+H]$^+$: 373.0626.

1.5.13 Enantiomeric separation of 6e

Enantiomeric separation of compound **6e** was performed by supercritical fluid chromatography (SFC) using a Waters ACQUITY UPC^2 system equipped with a photodiode array (PDA) detector and a QDa detector. Separations were carried out on a CHIRALPAK IG-3 column (Daicel; 150 mm × 3.0 mm i.d., 3

μm) maintained at 40 °C, using CO_2 (A) and EtOH (B) as the mobile phases. Isocratic elution was employed at 92% A / 8% B with a flow rate of 2.0 mL min^{-1} over a total run time of 7 min. The PDA was operated with a spectral acquisition range of 210-500 nm. The make-up pump flow rate was set to 0.4 mL min^{-1}.

1.6 ^{1}H NMR and ^{13}C NMR Spectra of Compounds

1.6.1 ^{1}H NMR (500 MHz) spectra of compound **6a** in DMSO-d_6.

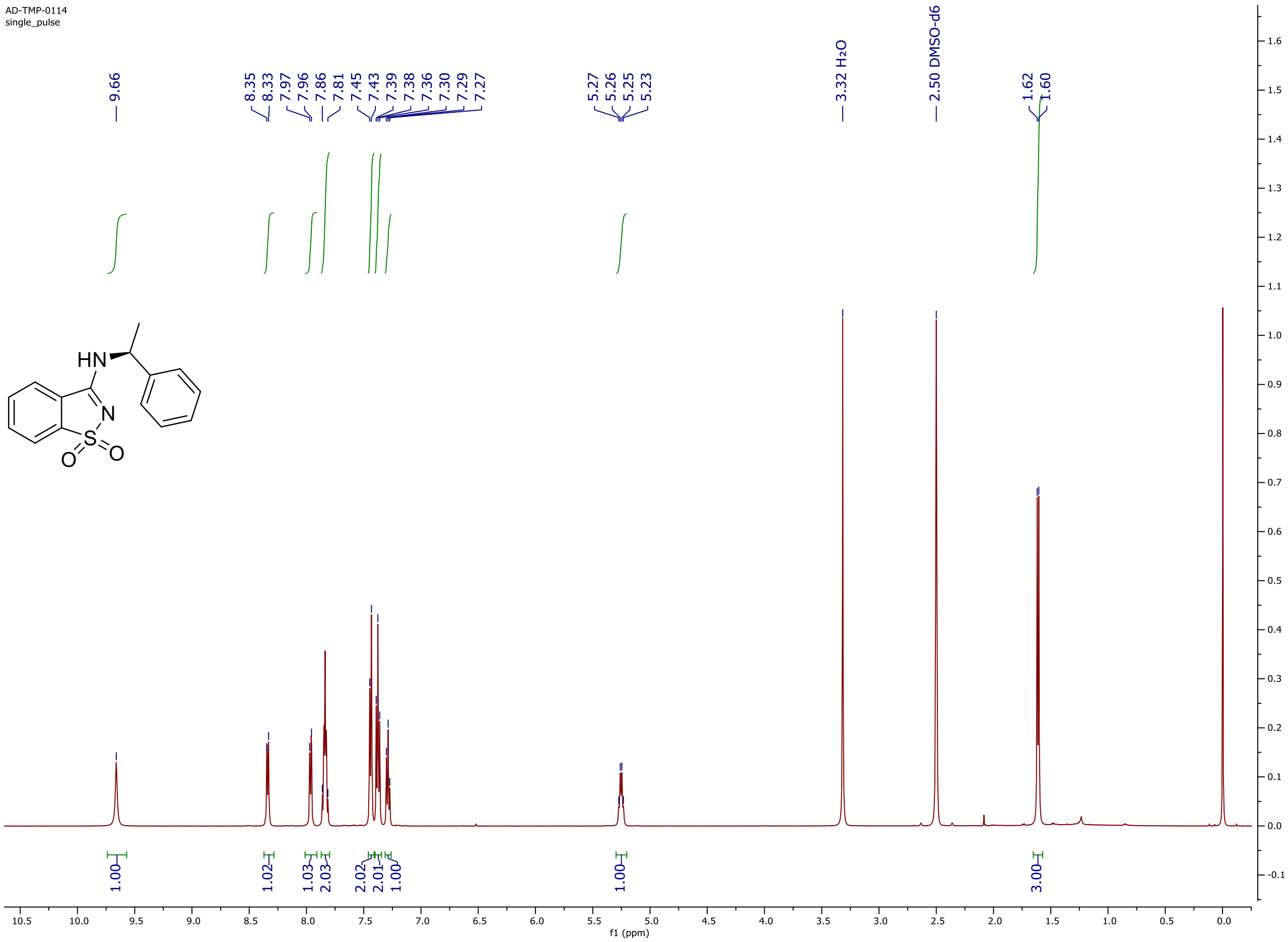

1.6.2 ^{13}C NMR (125 MHz) spectra of compound **6a** in DMSO-d_6

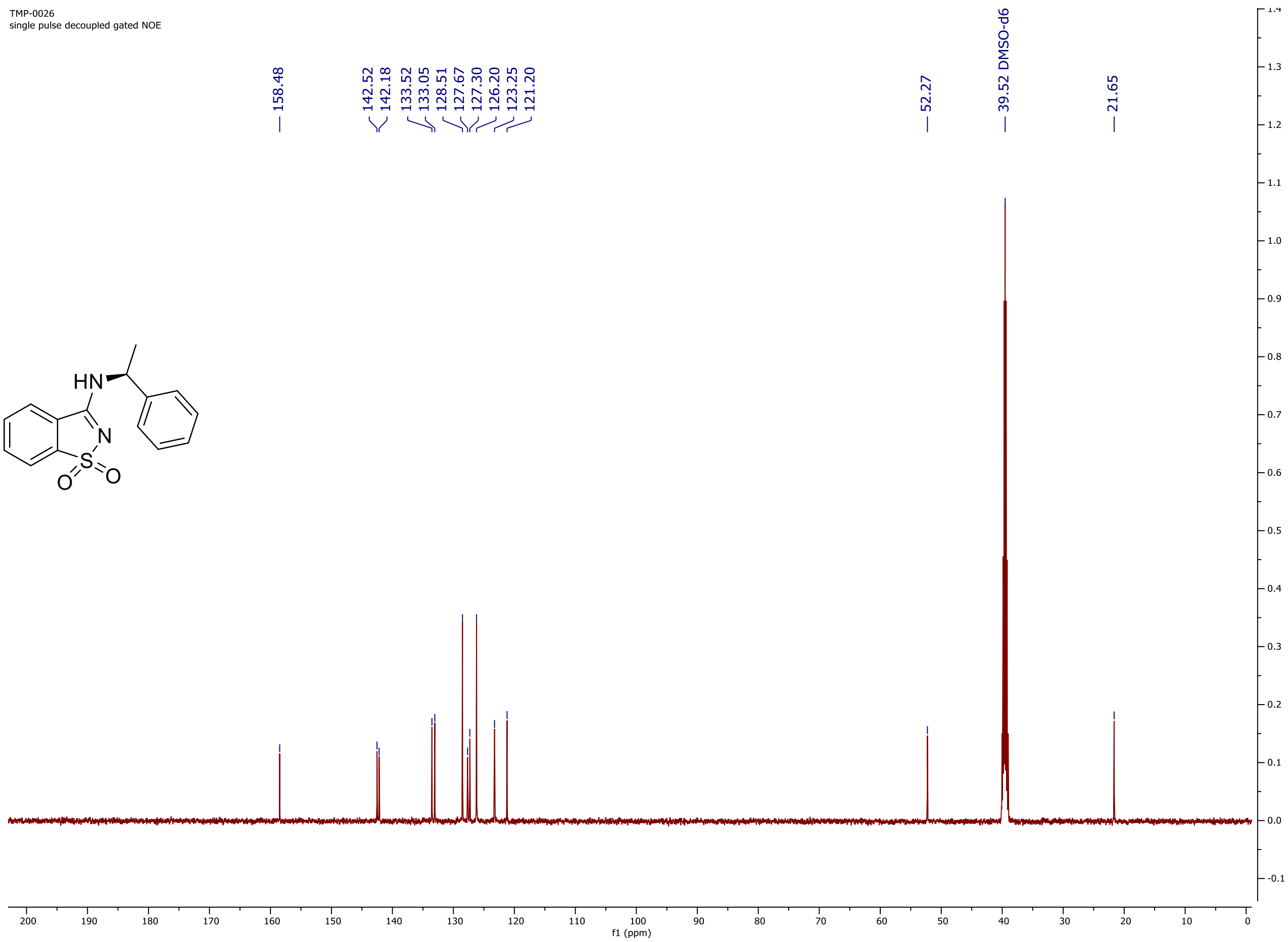

1.6.3 HPLC chromatogram and HRMS spectrum of compound **6a**.

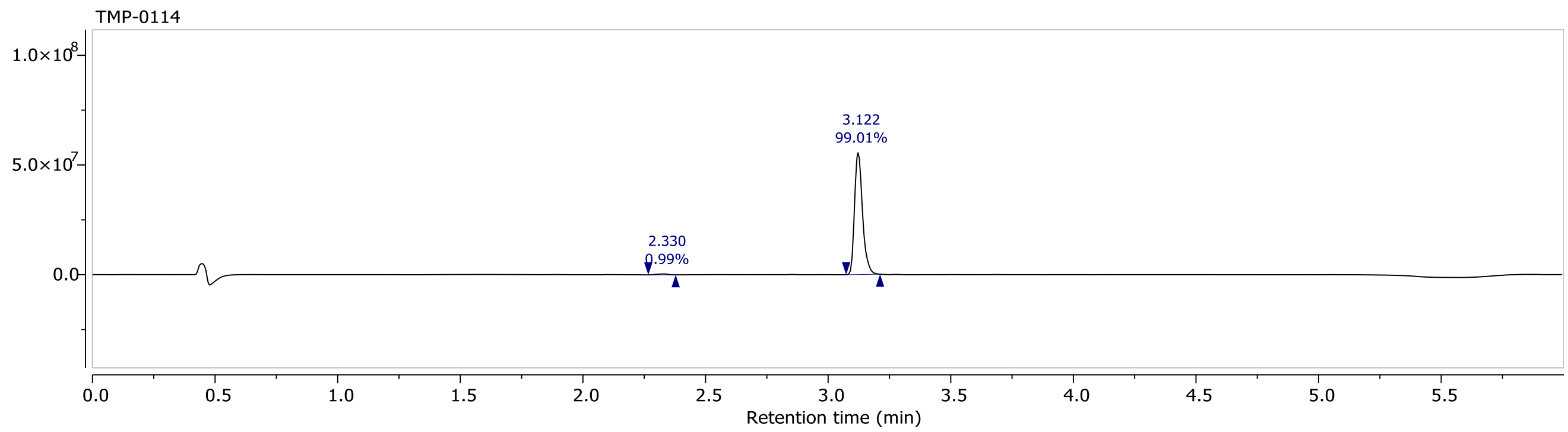

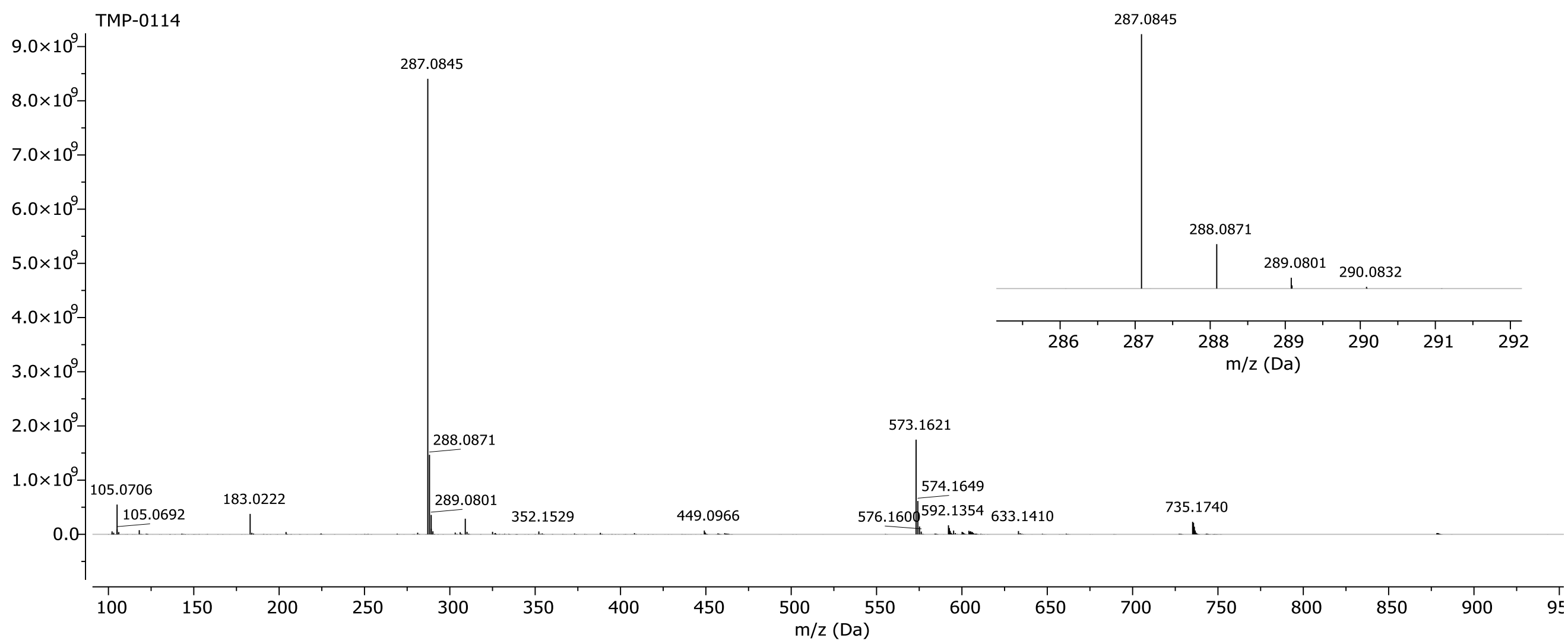

Calculated to $C_{15}H_{14}N_2O_2S$ $[M+H]^+$: 287.0848; found $[M+H]^+$: 287.0845 (**error:** 1.04 ppm);

1.6.4 ^{1}H NMR (500 MHz) spectra of compound **6b** in DMSO-d_6.

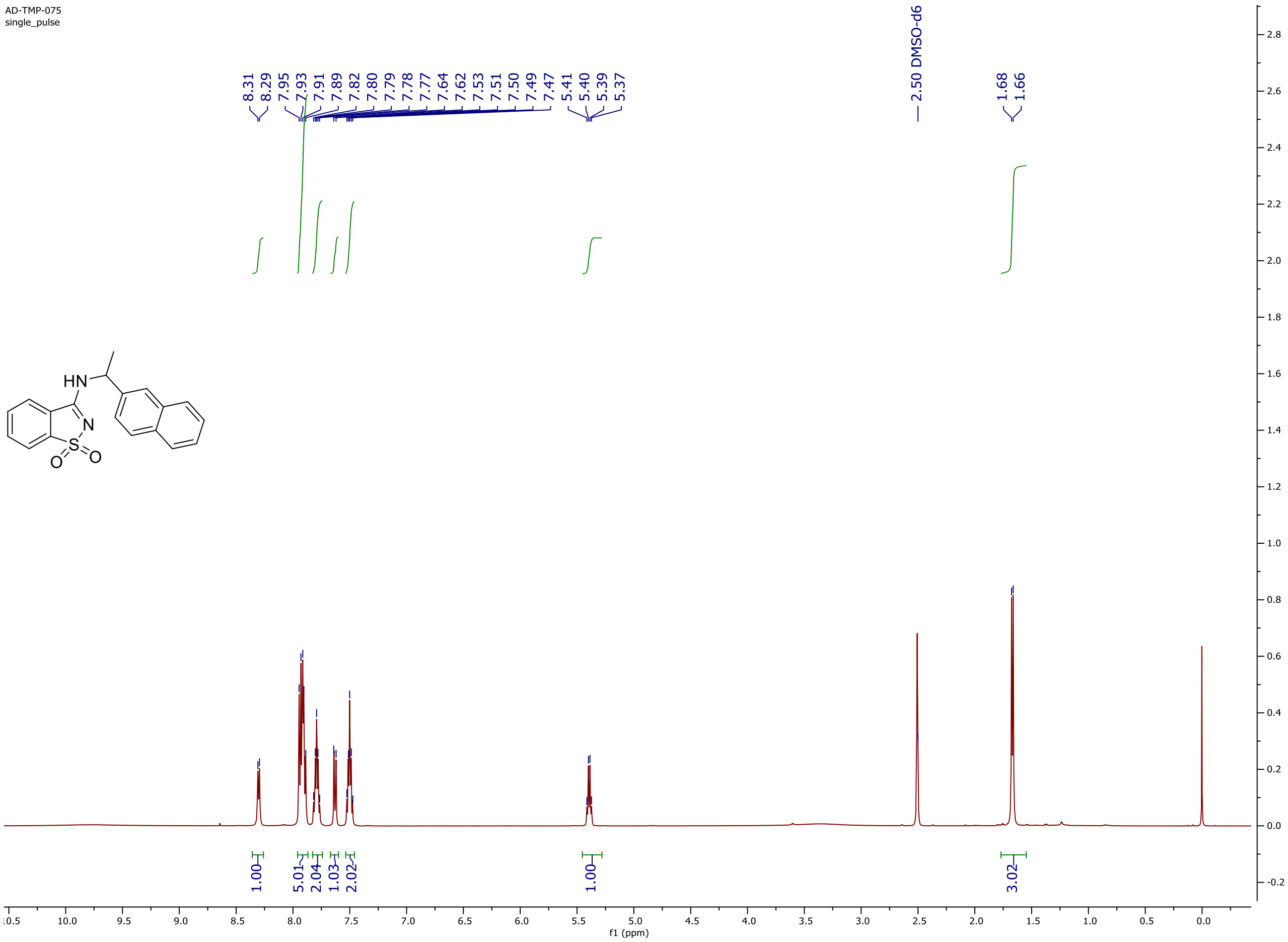

1.6.5 ^{13}C NMR (125 MHz) spectra of compound **6b** in DMSO-d_6.

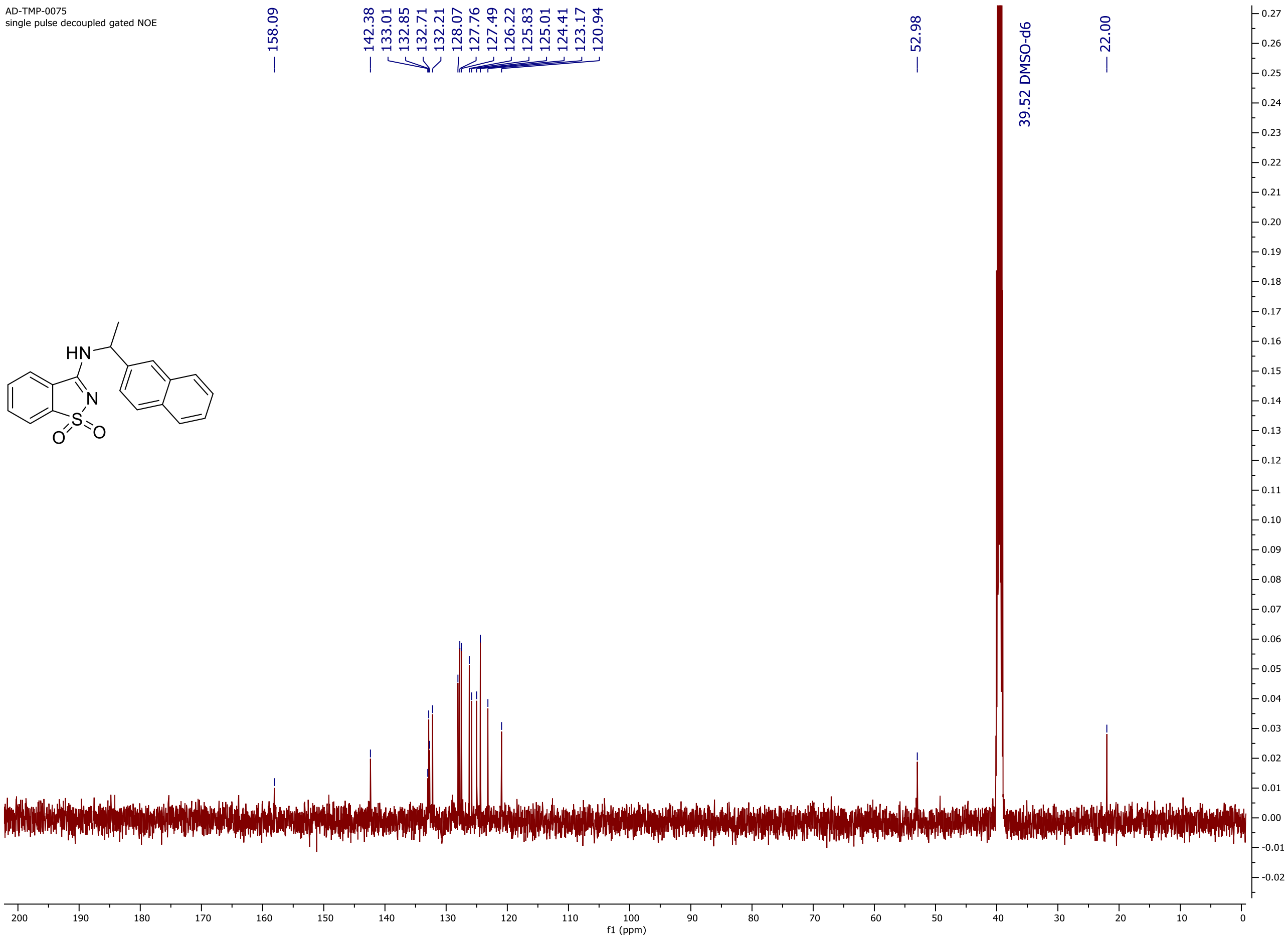

1.6.6 HPLC chromatogram and HRMS spectrum of compound **6b.**

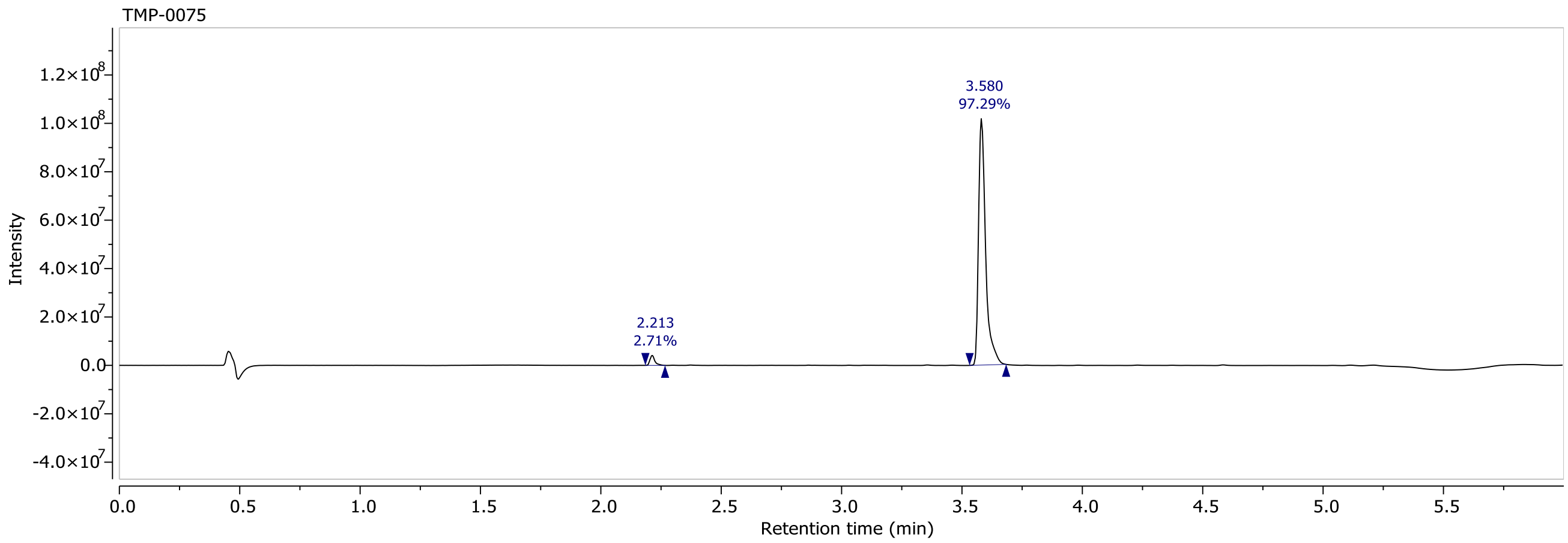

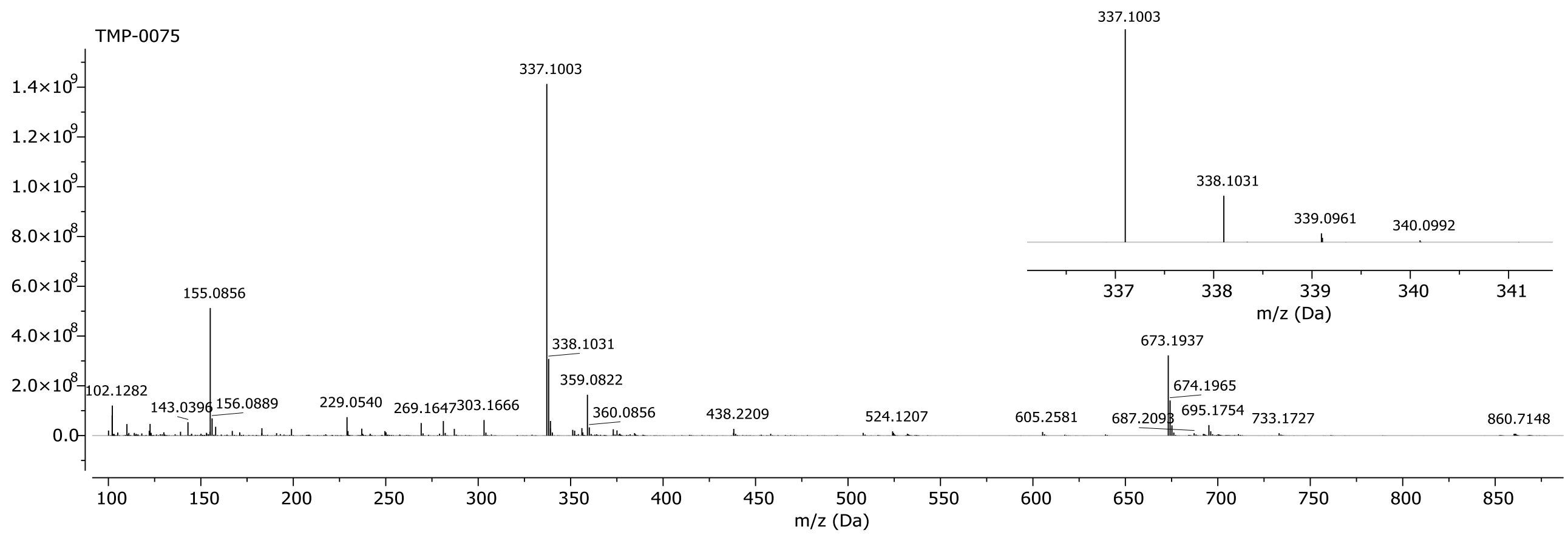

Calculated to $C_{19}H_{16}N_2O_2S$ $[M+H]^+$: 337.1005; found $[M+H]^+$: 337.1003 (**error:** 0.59 ppm);

1.6.7 ^{1}H NMR (500 MHz) spectra of compound **6c** in DMSO-*d_6*.

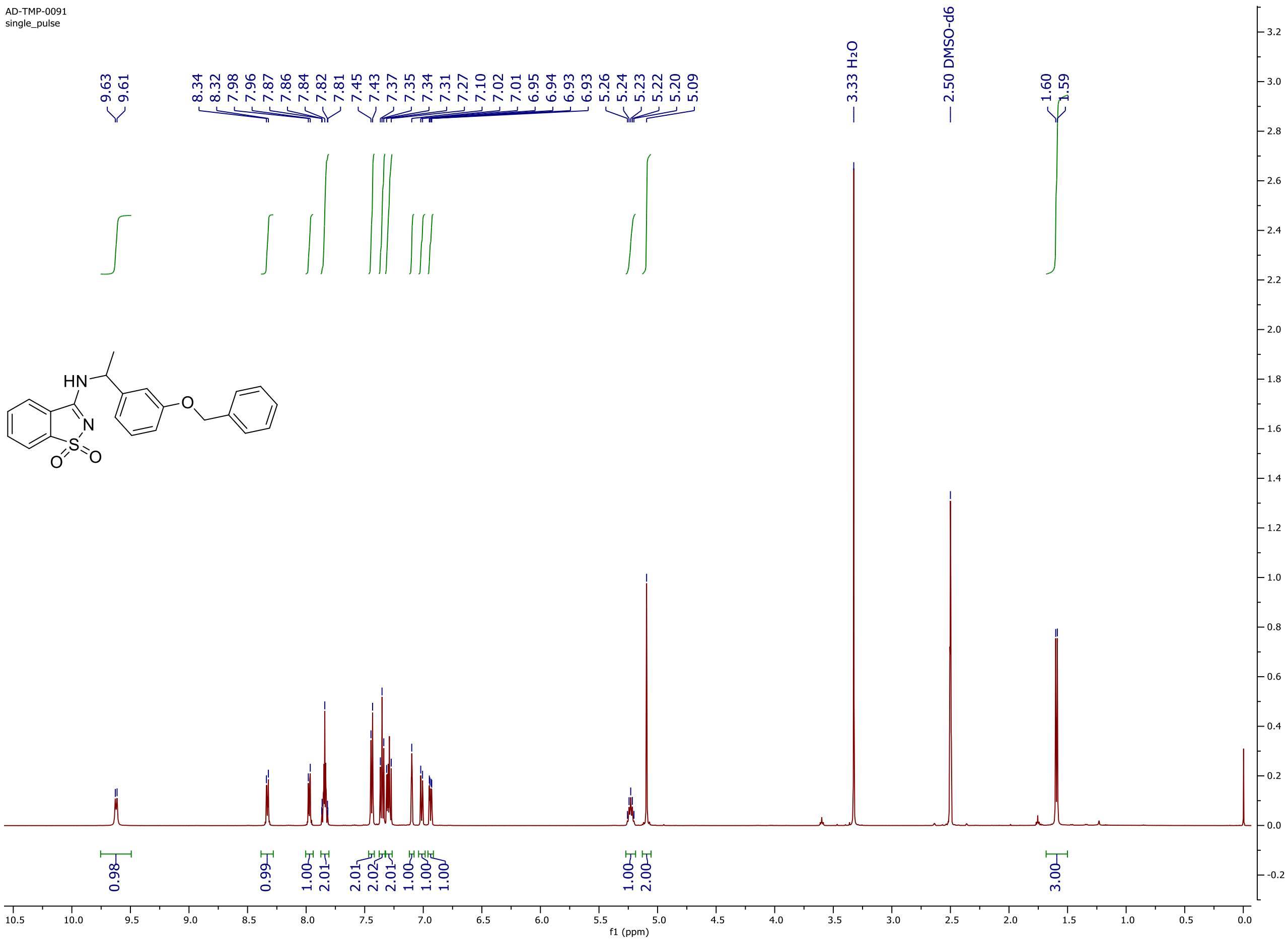

1.6.8 ^{13}C NMR (125 MHz) spectra of compound **6c** in DMSO-d_6.

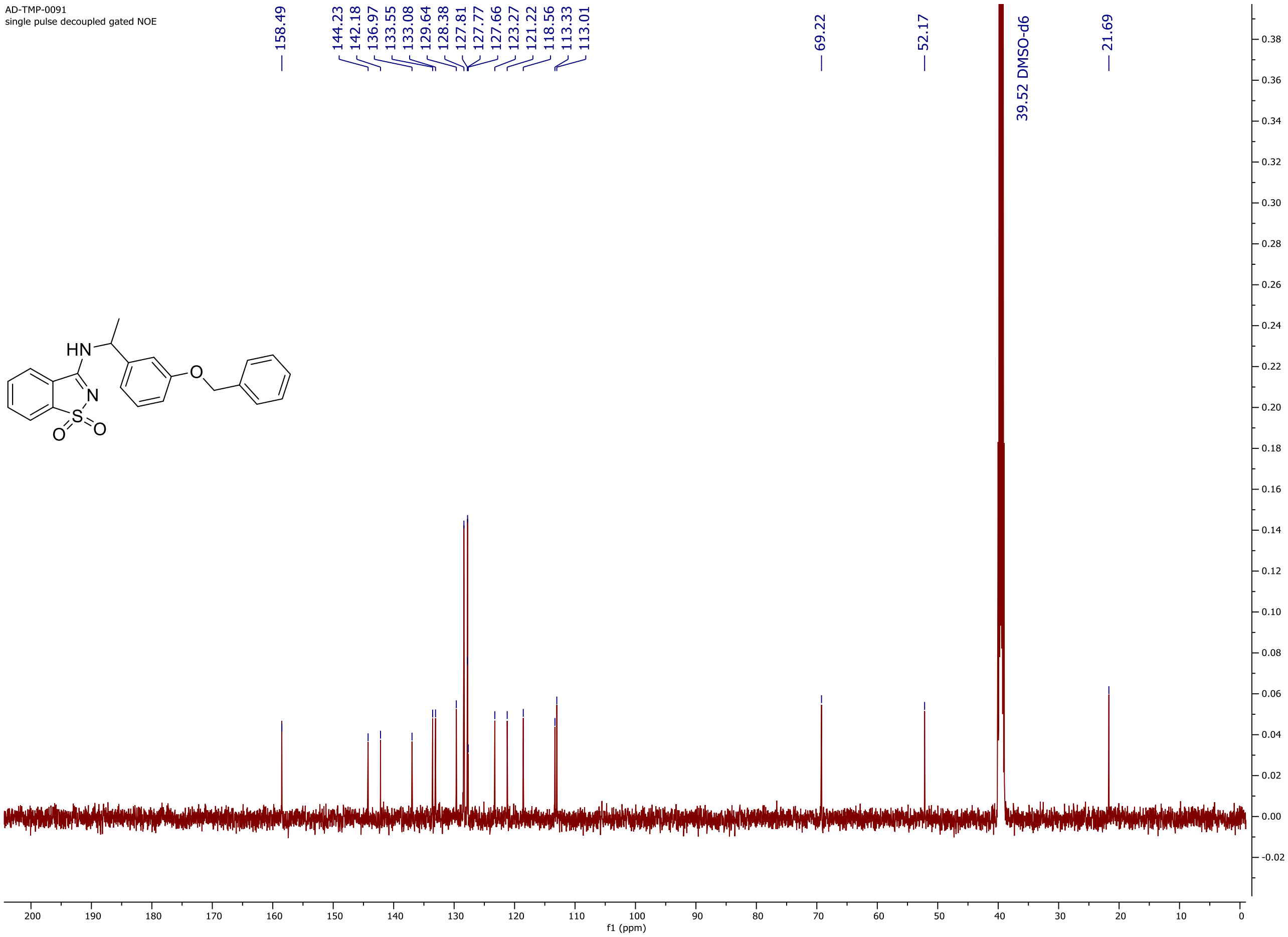

1.6.9. HPLC chromatogram and HRMS spectrum of compound **6c**.

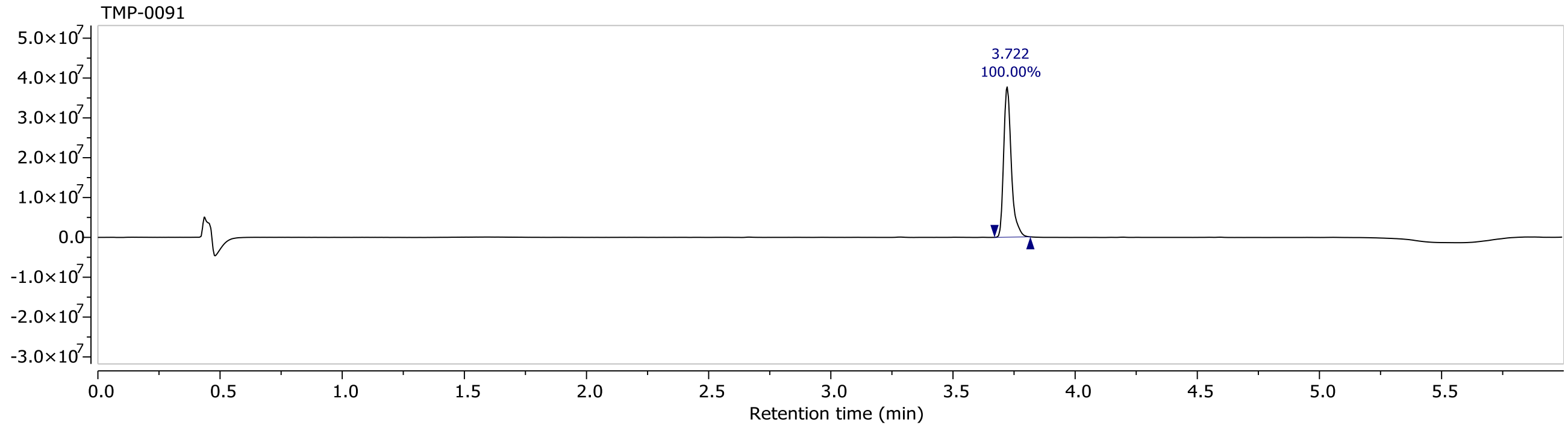

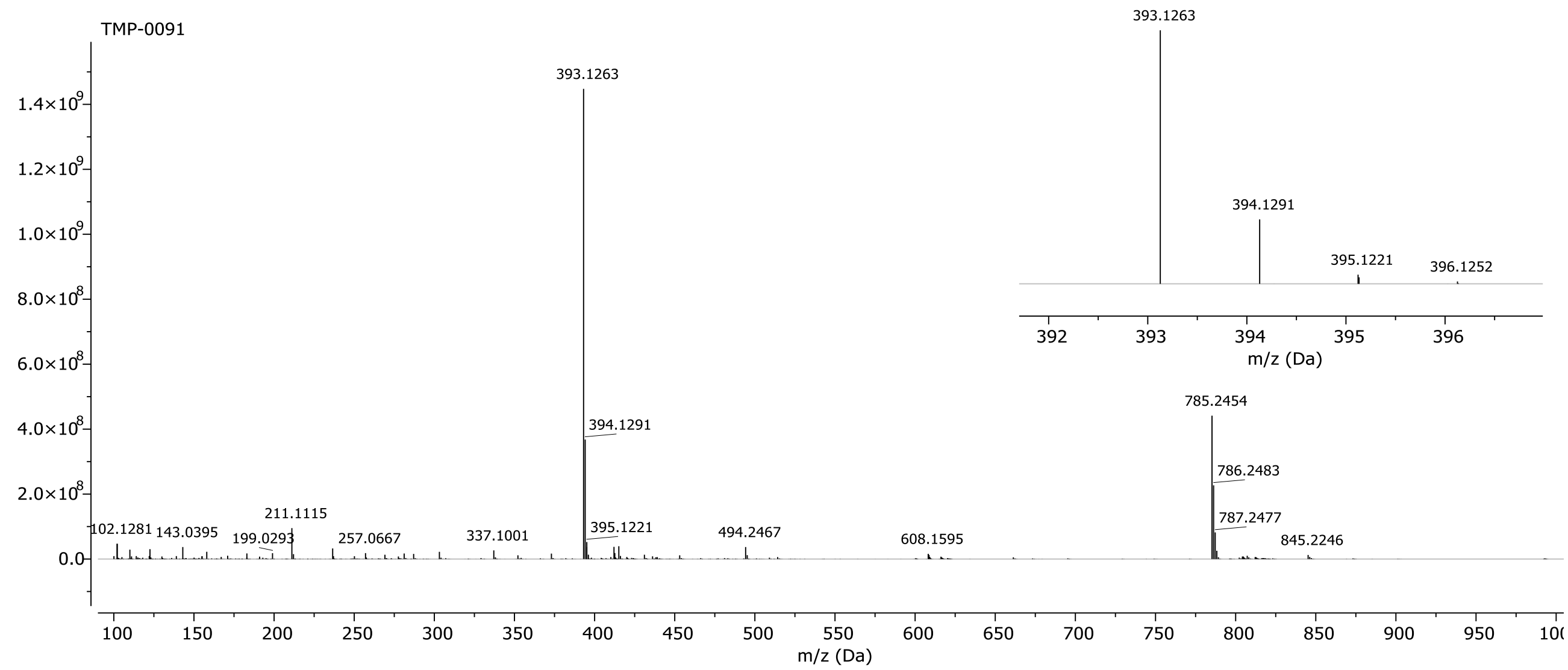

Calculated to $C_{22}H_{20}N_2O_3S$ $[M+H]^+$: 393.1267; found $[M+H]^+$: 393.1263 (**error:** 1.01 ppm);

1.6.10. ^{1}H NMR (500 MHz) spectra of compound **6d** in DMSO-d_6.

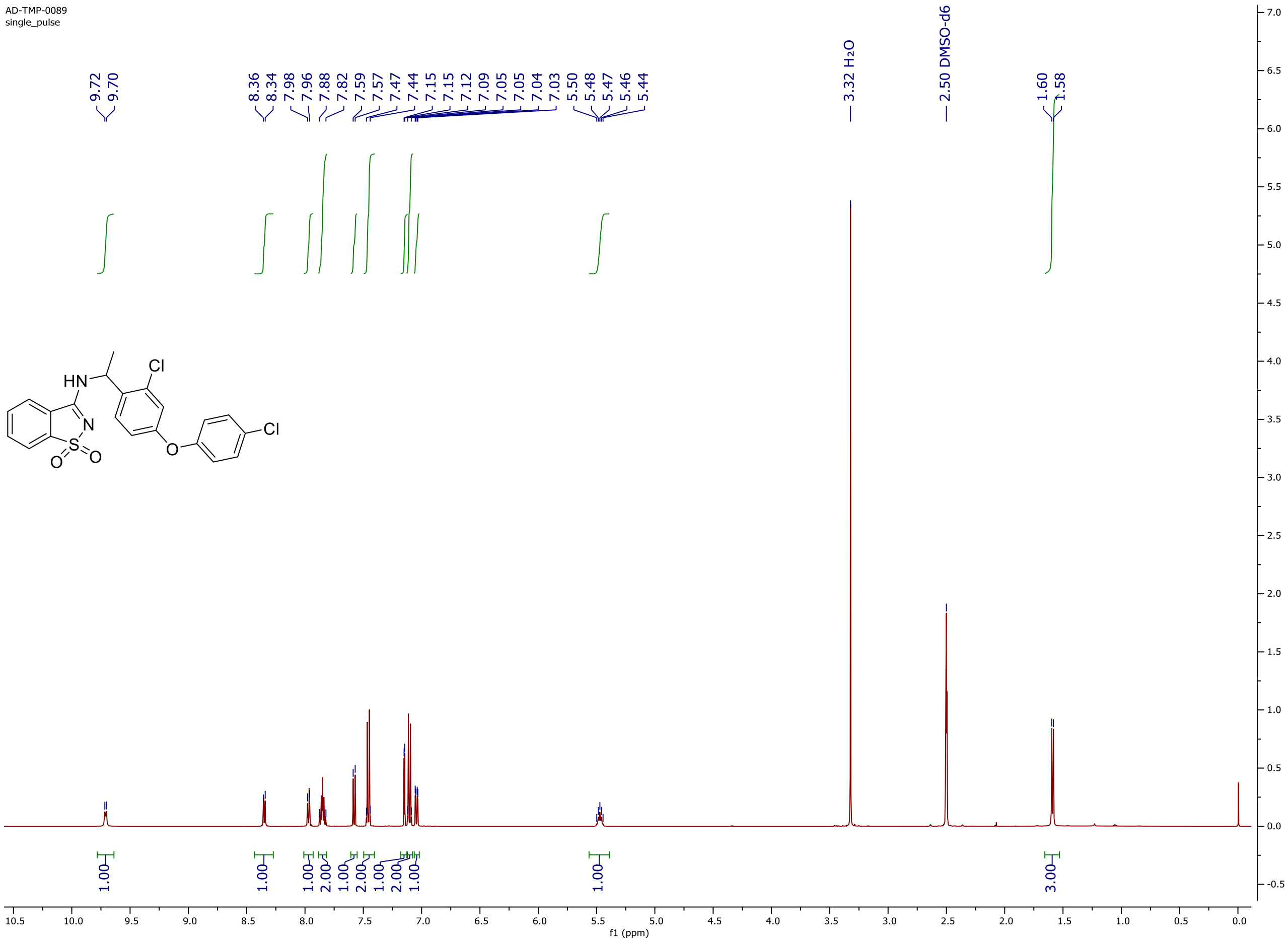

1.6.11. ^{13}C NMR (125 MHz) spectra of compound **6d** in DMSO-d_6.

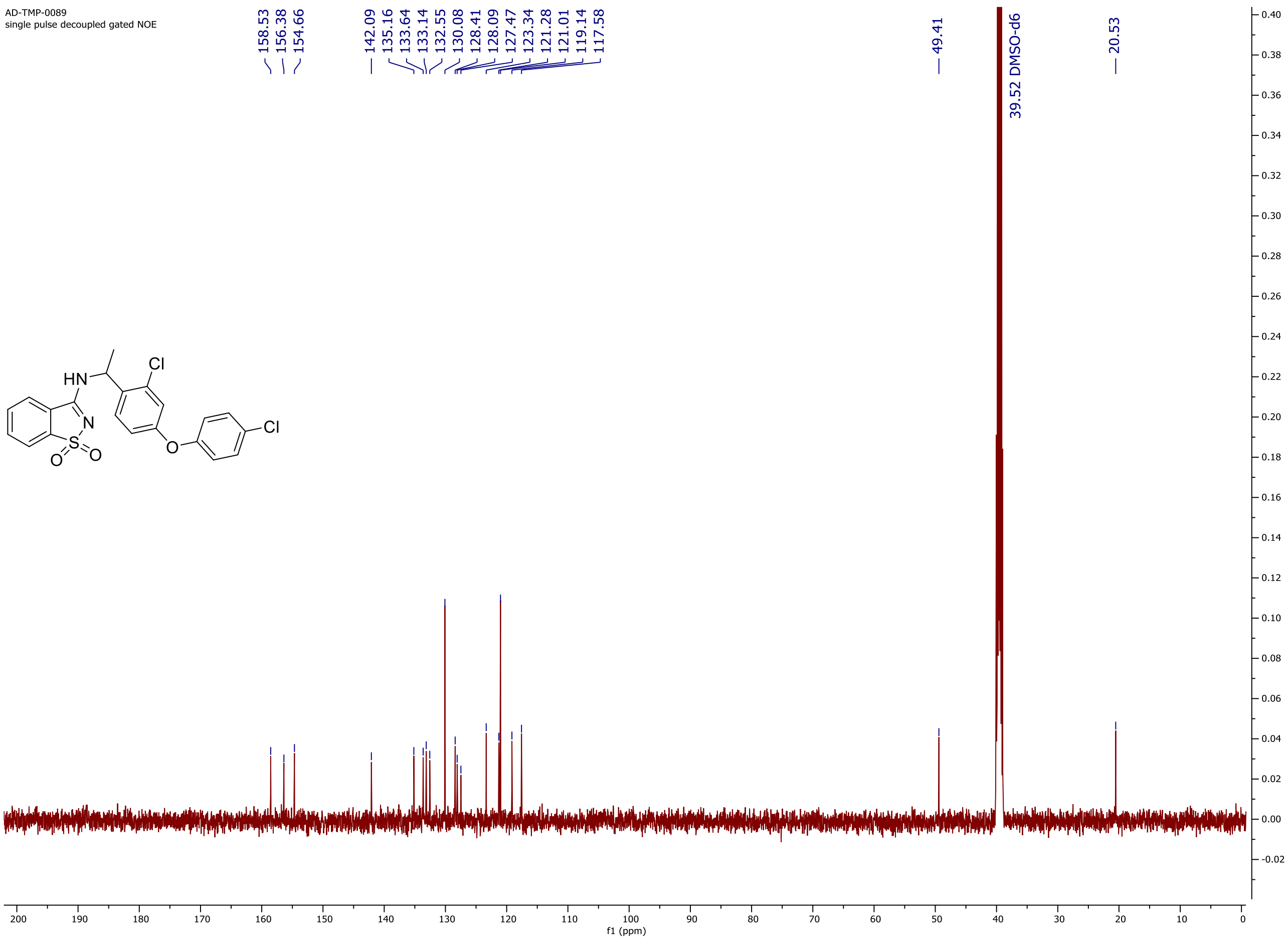

1.6.12. HPLC chromatogram and HRMS spectrum of compound **6d**.

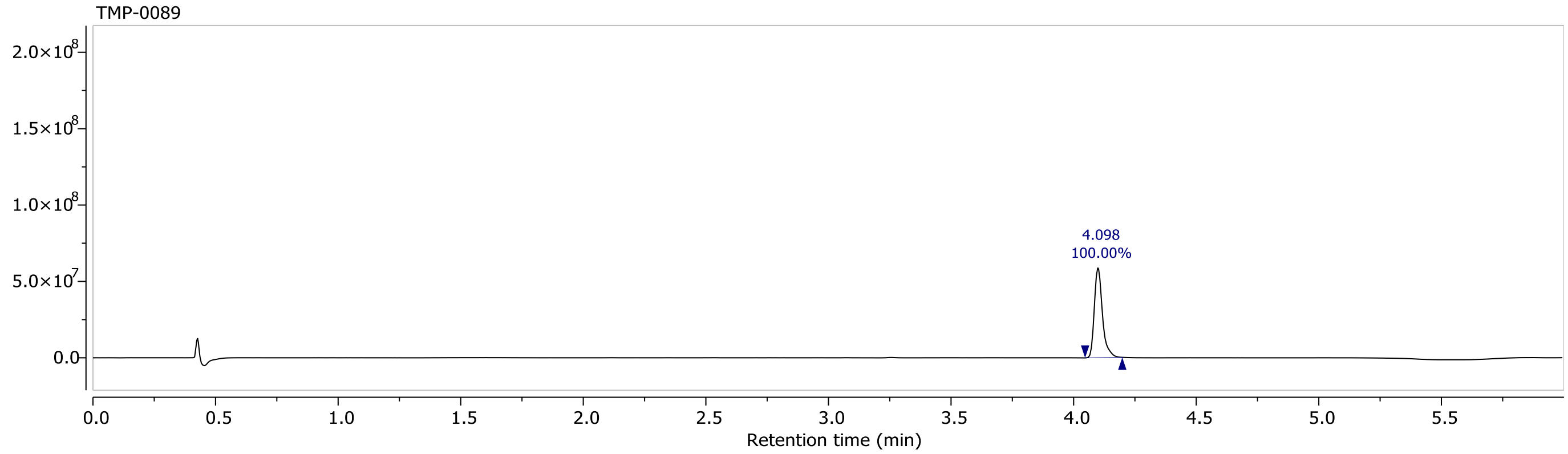

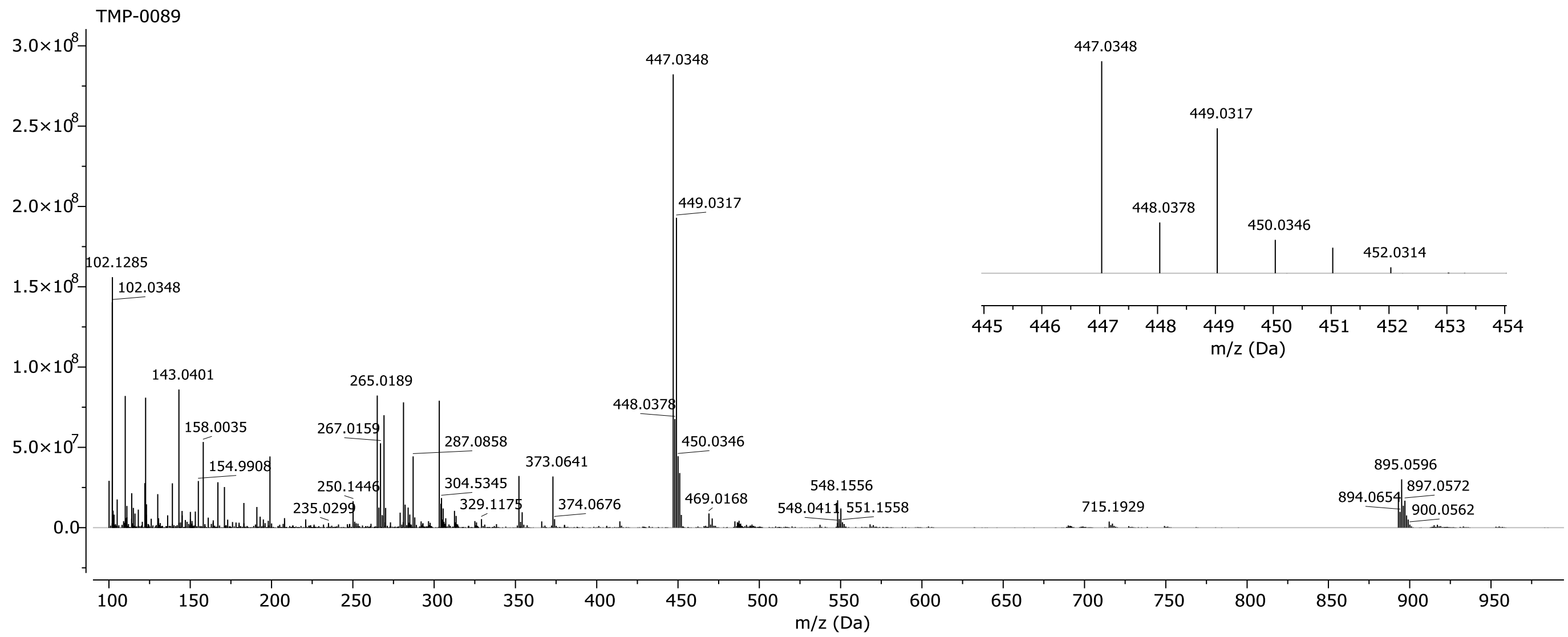

Calculated to $C_{21}H_{16}Cl_2N_2O_3S$ $[M+H]^+$: 447.0331; found $[M+H]^+$: 447.0348 (**error:** 3.8 ppm);

1.6.13. ^{1}H NMR (500 MHz) and ^{13}C NMR (125 MHz) spectra of compound **6e** in DMSO-*d*$_6$.

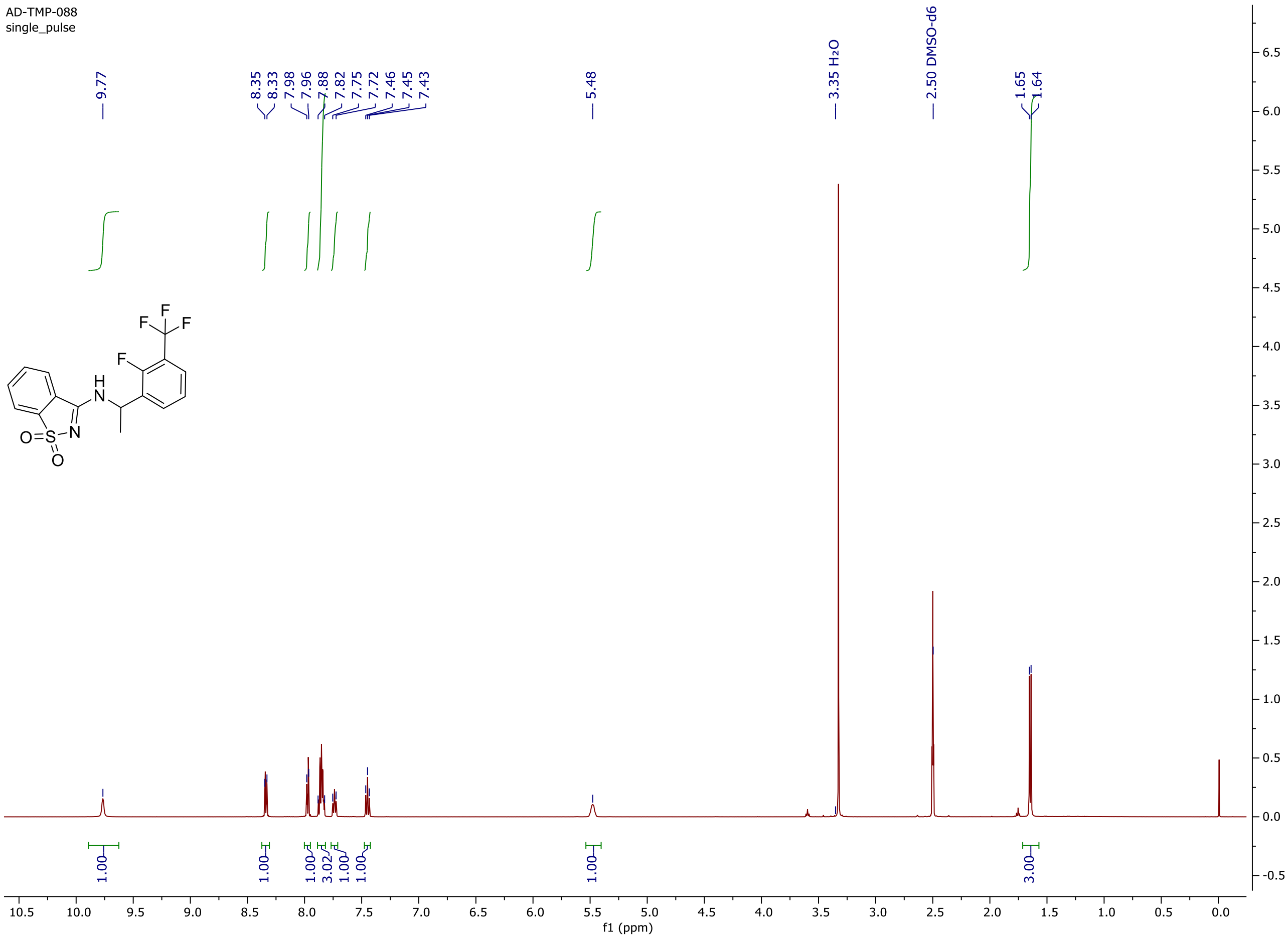

1.6.14. ^{13}C NMR (125 MHz) spectra of compound **6e** in DMSO-*d*6.

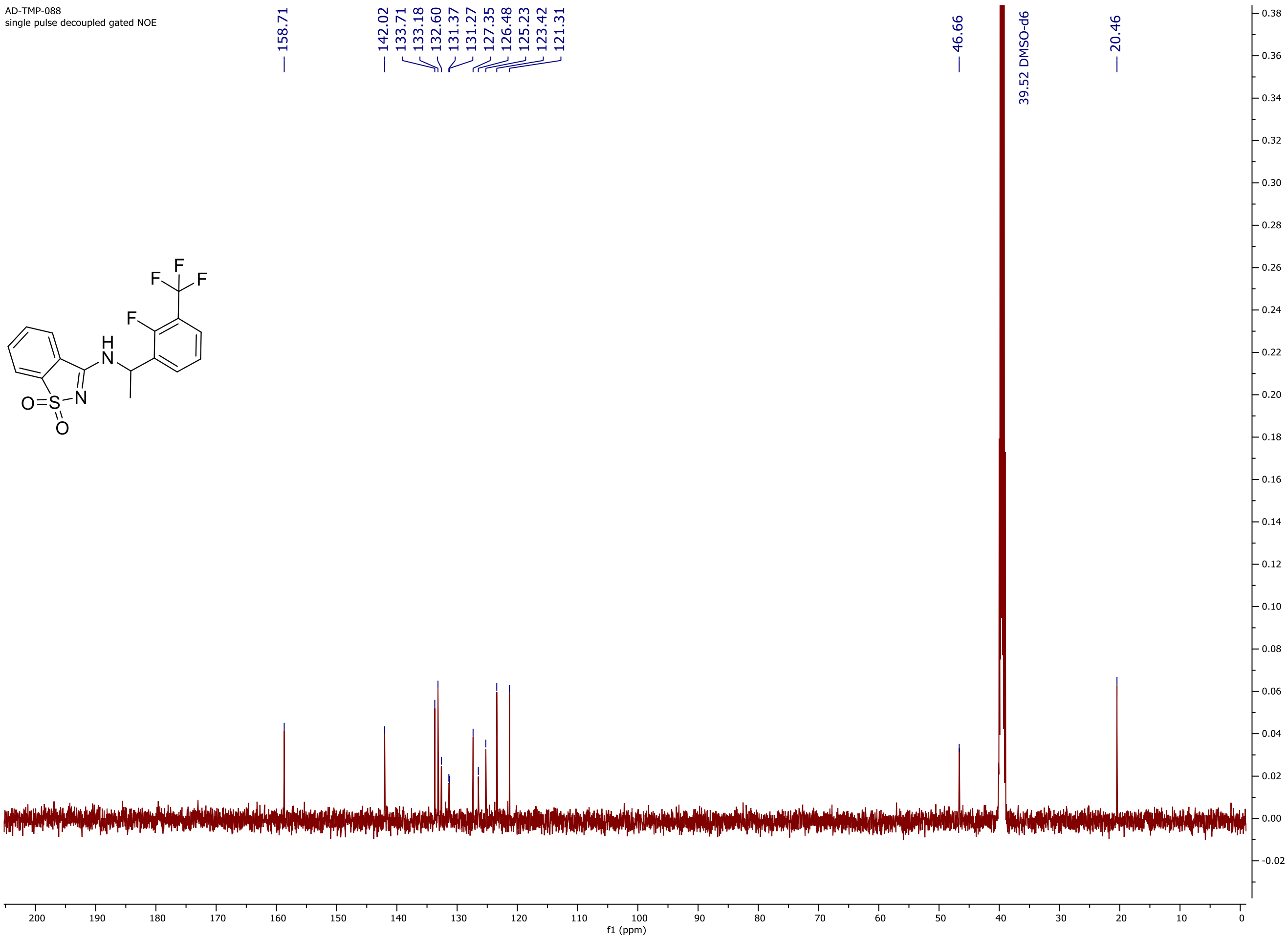

1.6.15. HPLC chromatogram and HRMS spectrum of compound **6e**.

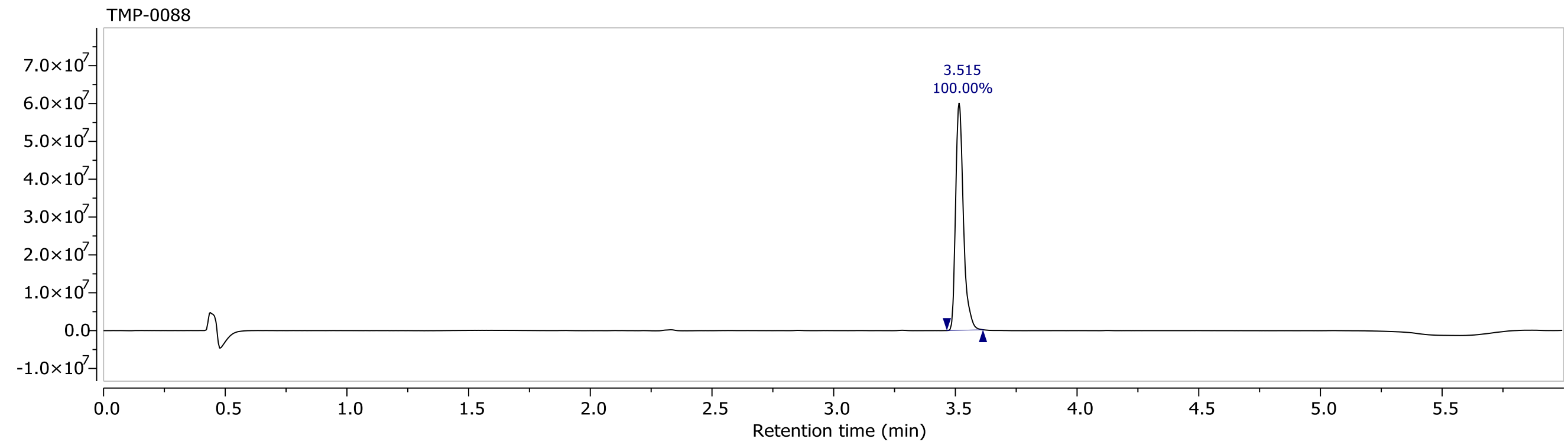

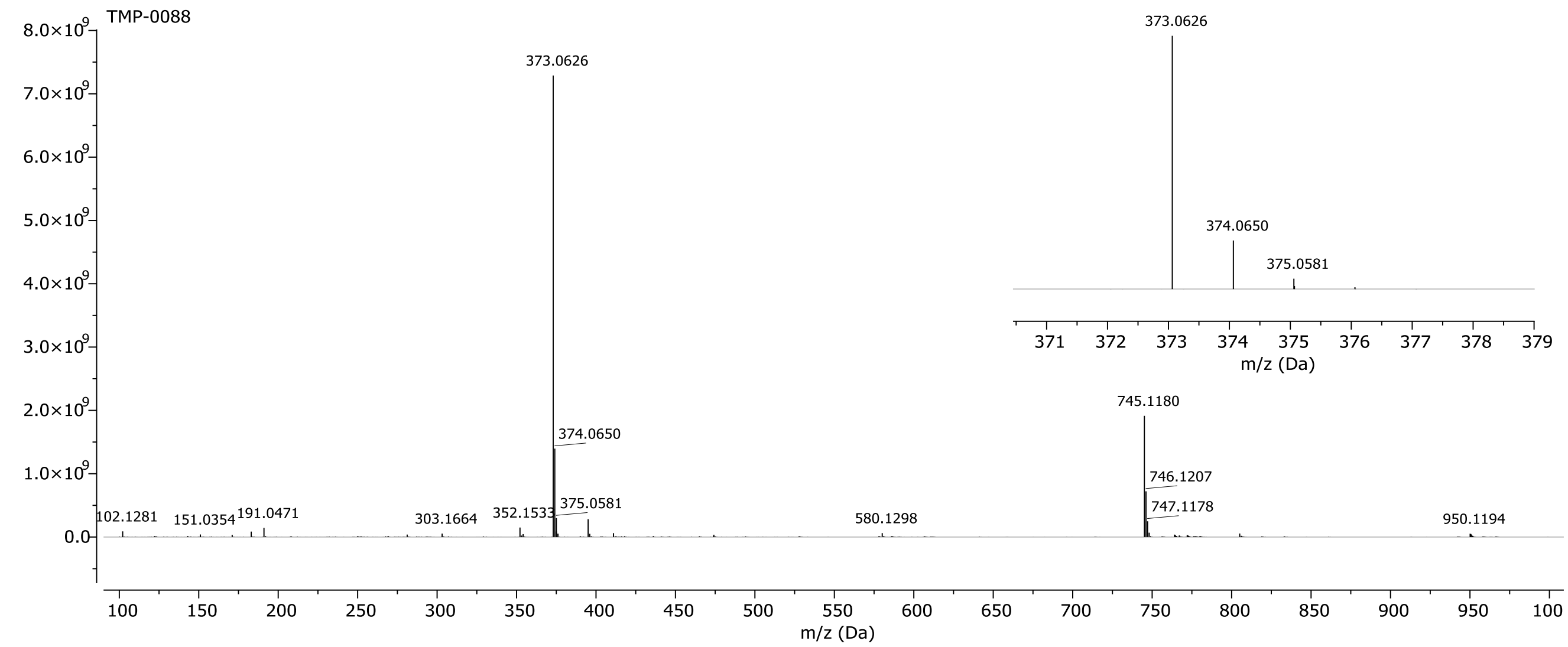

Calculated to $C_{16}H_{12}F_4N_2O_2S$ $[M+H]^+$: 373.0628; found $[M+H]^+$: 373.0626 (**error:** 0.53 ppm);

2. COMPOUND STRUCTURAL ELUCIDATION BY X-RAY CRYSTALLOGRAPHY

X-ray diffraction studies were conducted to support the identification of the products, define the absolute configuration of enantiopure compounds, and characterize the three-dimensional features of the molecules. In detail, structural data were obtained for compounds **1d**, **3b**, **3d**, **6a-d**. In this section, we will discuss the structures of **6a** and **6c**; crystallographic data of the remaining products can be found in the next paragraphs.

Compound **6a** crystallized as colorless plates from the slow evaporation of a methanol solution at room temperature. Indexing of the diffraction pattern suggested an I-centered monoclinic unit cell, with systematic absences compatible with the space group I2. The asymmetric unit, containing one molecule of the compound, is shown in **Figure S3A** as a displacement ellipsoid diagram. **6a** features a chiral center at C8, which was assigned the (*S*) absolute configuration based on the Flack parameter (Parsons' method).[2] Structurally, the compound comprises two planar fragments, namely the 3-aminobenzo[*d*]isothiazole 1,1-dioxide moiety and the phenyl group, linked by the stereogenic carbon C8. The mean planes of the two fragments are inclined at 105.8°, with the N2–C8–C10–C15 dihedral angle being 107.1(3)°. A Mogul geometry analysis indicated that all bond lengths and angles fall within expected ranges, except for the C7–C1–N2 angle, which is reduced to 121.8(2)° compared with the average of 123.4(6)°. This deviation is consistent with the observed intermolecular interaction pattern, involving a strong N–H···O hydrogen bond between N2–H2 and the sulfoxide oxygen atom O1 (D···A/Å = 2.96(1); D–H···A/° = 170(3)°). The latter also participates in a weaker C–H···O contact involving C6–H6 (D···A/Å = 3.38(1); D–H···A/° = 155.1(2)°). Due to the geometry and directionality of these interactions, the molecule effectively acts as a bidentate hydrogen-bond donor, with the relatively flexible C7–C1–N2 angle slightly compressed relative to unrestrained congeners. Similar behaviors can be observed in other literature structures exhibiting comparable interaction patterns.[3] These contacts generate molecular chains elongating along the *b* axis (**Figure S3B**). The second oxygen atom of the sulfoxide group (O2) is involved in a weak but significant C–H···O contact with C12–H12 (D···A/Å = 3.50(1); D–H···A/° = 162.2(2)°), resulting in orthogonal connections between parallel chains along the *a* axis. The extensive aromatic character of **6a** also enables π-π interactions (**Figure S3C**). The strongest of these is a nearly T-shaped contact between the phenyl ring and the benzo[*d*]isothiazole 1,1-dioxide moiety (centroid-centroid distance: 4.84 Å; relative orientation: 77.5°), linking molecules along the *c* axis. In the same direction, a weaker inclined stacking interaction connects adjacent molecules through their phenyl rings (centroid-centroid distance: 4.99 Å; relative orientation: 43.2°). Finally, almost parallel heteroaromatic stacking interactions between the benzo[*d*]isothiazole 1,1-dioxide moieties (centroid-centroid distances: 3.94 and 4.03 Å; relative orientation: 9.3°) reinforce the connectivity along the *a* axis.

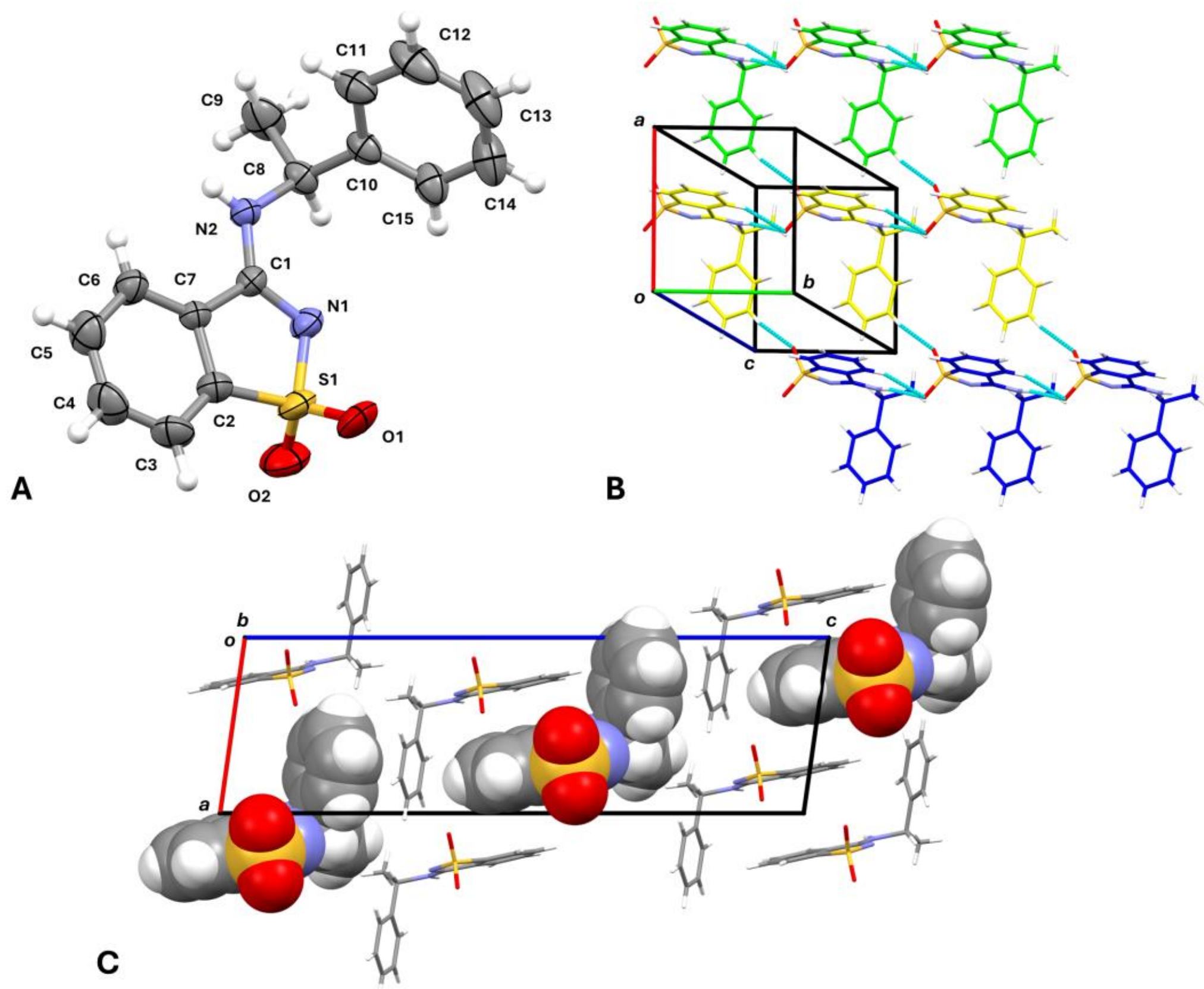

Figure S3. A. Thermal-ellipsoid diagram of **6a**, with an arbitrary atom- numbering scheme. Displacement ellipsoids are set at the 40% probability level. **B.** Stick model showing the main hydrogen bonds in an arbitrary view. The different colors facilitate the identification of the chains propagating along the *b* axis. **C.** Stick/spacefill model highlighting the main π-π stacking interactions viewed along the *b* axis.

The intermolecular interaction pattern was further examined by Hirshfeld surface (HS) analysis (HS data: V = 354.68 Å^3, A = 326.48 Å^2, G = 0.742, Ω = 0.119). The d_{norm} property mapped onto the HS features one prominent red region, corresponding to the short-range N–H···O contact, together with two weaker red areas associated with C–H···O interactions (**Figure S4A**). The corresponding two-dimensional fingerprint plot exhibits a relatively elongated shape, with two spikes extending towards the lower left region of the graph, indicating short-range contacts (**Figure S4B**). An arrow-shaped green region at the center of the plot further supports the presence of parallel stacking interactions, which is also corroborated by a large flat region observed on the curvedness surface (**Figure S4C**). Furthermore, the shape-index mapping aids the visualization of the strong T-shaped interaction between the phenyl ring and the benzo[*d*]isothiazole 1,1-dioxide moiety (**Figure S4D**). The calculation of the enrichment ratios, carried out following Jelsch's method, indicates that N···H/H···N, O···H/H···O, and C···C contacts are favored, further supporting the results of the structural study. Finally, CE-B3LYP interaction-energy analysis, based on B3LYP/6-31G(d,p) electron densities, reveals a balance between directional

electrostatic interactions and multiple dispersion-dominated contacts. The two most stabilizing interactions exhibit total energies (E_{tot}) of −57.6 and −51.0 kJ mol^{-1}, the former being dominated by electrostatic contributions and the latter largely driven by dispersion (**Figure S4E** and **S4F**).

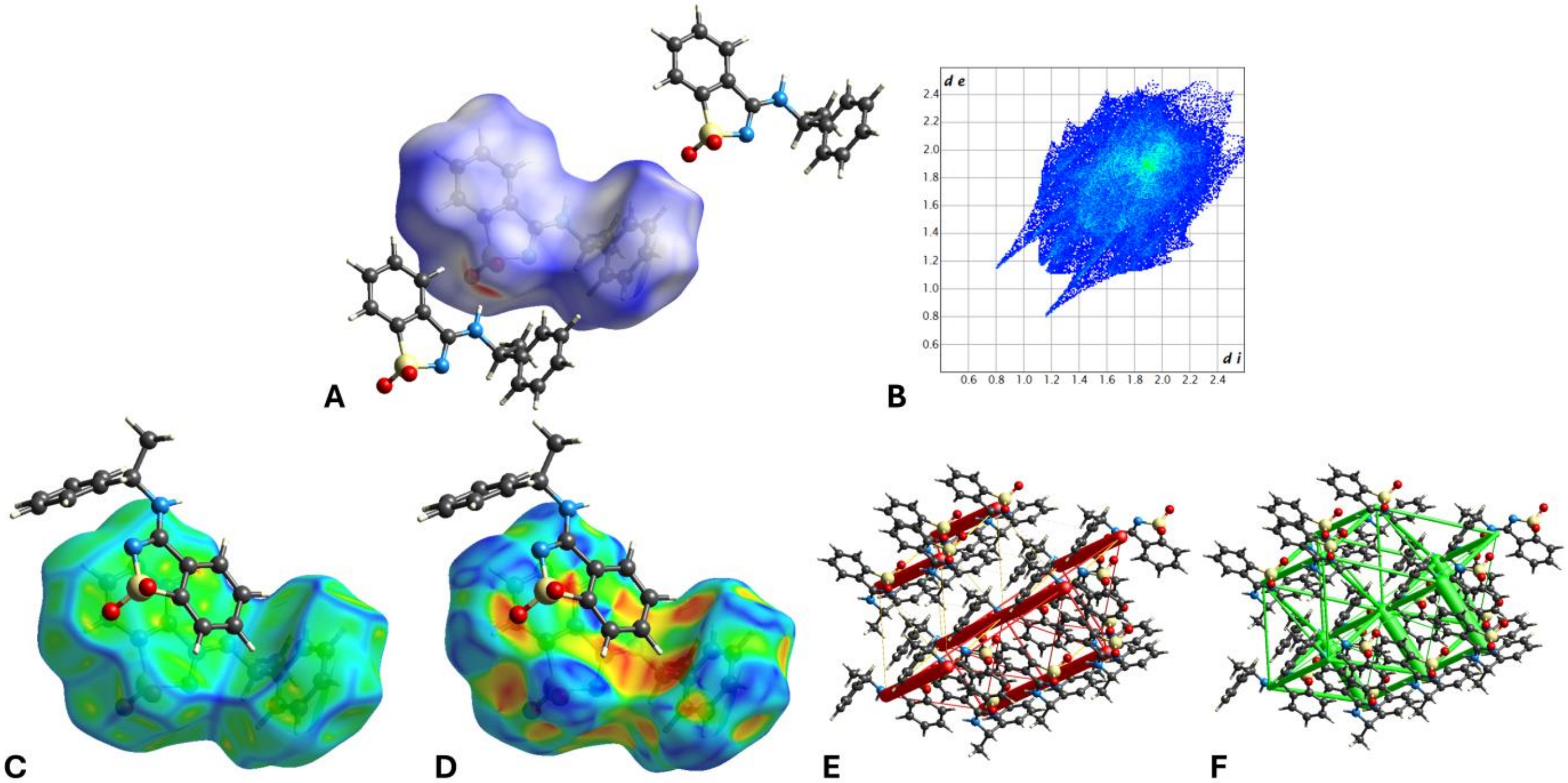

Figure S4. A. Three-dimensional representation of d_{norm} mapped onto the HS of **6a** (red-white-blue color scale: −0.4973 to 1.4065 a.u.). **B.** Two-dimensional Hirshfeld fingerprint plot (d_e vs d_i) derived from the d_{norm} surface. **C.** Three-dimensional representation of the curvedness property mapped onto the HS of **6a** (green-to-blue color scale: −3.8358 to 0.4256 a.u.). **D.** Three-dimensional representation of the shape index property mapped onto the HS of **6a** (red-to-blue color scale: −0.9916 to 0.9979 a.u.). **E-F.** Coulomb (**E**) and dispersion interaction energies (**F**) visualized as red and green cylinders, respectively, connecting molecular centroids and scaled to the interaction energy magnitude.

Compound **6c** crystallized as colorless plates from the slow evaporation of a methanol solution at room temperature. Diffraction data were consistent with a monoclinic unit cell in space group $P2_1/c$; the asymmetric unit is shown in **Figure S5A** as a displacement ellipsoid diagram. Unlike **6a**, **6c** is a racemic mixture featuring an additional benzyloxy substituent at the 3-position of the phenyl ring. Structurally, the molecule adopts a bend like that of its analogue, with the 3-aminobenzo[*d*]isothiazole 1,1-dioxide moiety inclined by 97.8° relative to the phenyl ring, which in turn forms an angle of 31.5° with the phenyl group of the benzyloxy substituent. The associated dihedral angles are 123.0(2)° for N2–C8–C10–C15 and 161.2(2)° for O3–C16–C17–C18. As observed for **6a**, bond lengths and angles fall within expected ranges, except for the C7–C1–N2 angle, which measures 121.5(1)°. The observed angular deviation arises from a strong N2–H2⋯O2 hydrogen bond involving the benzo[*d*]isothiazole 1,1-dioxide moiety (D⋯A/Å = 3.06(1); D–H⋯A/° = 163(2)°). The same oxygen atom (O2) also participates in a weaker interaction with C6–H6 (D⋯A/Å = 3.43(1); D–H⋯A/° = 157.1(1)°), while the second sulfoxide oxygen

(O1) is engaged in medium-to-long-range contacts with C3–H3 (D···A/Å = 3.25(1); D–H···A/° = 151.2(1)°) and C11–H11 (D···A/Å = 3.83(1); D–H···A/° = 164.6(1)°). Collectively, these interactions give rise to one-dimensional double-stranded chains propagating along the *a* axis, in which the benzisothiazole sulfone cores stack face-to-face and the aryl arms extend in opposite directions (**Figure S5B**). Within each strand, molecules are connected by the N2–H2···O2, C6–H6···O2, and C11–H11···O1 interactions, whereas strand pairing is mediated by the C3–H3···O1 contact. Notably, the dominant interaction between the two strands is a parallel π–π stacking between the heterocyclic cores (centroid–centroid distance: 3.69 Å). In addition, a weaker inclined π–π contact between the benzyloxy group and the phenyl ring further consolidates the intrastrand assembly (centroid–centroid distance: 5.18 Å; relative orientation: 31.5°). In contrast, interactions between adjacent chains are significantly weaker, with a C–H···O hydrogen bond between C21–H21 and O3 (D···A/Å = 3.41(1); D–H···A/° = 132.5(2)°) and minor C–H···π contacts that have a negligible impact on the packing.

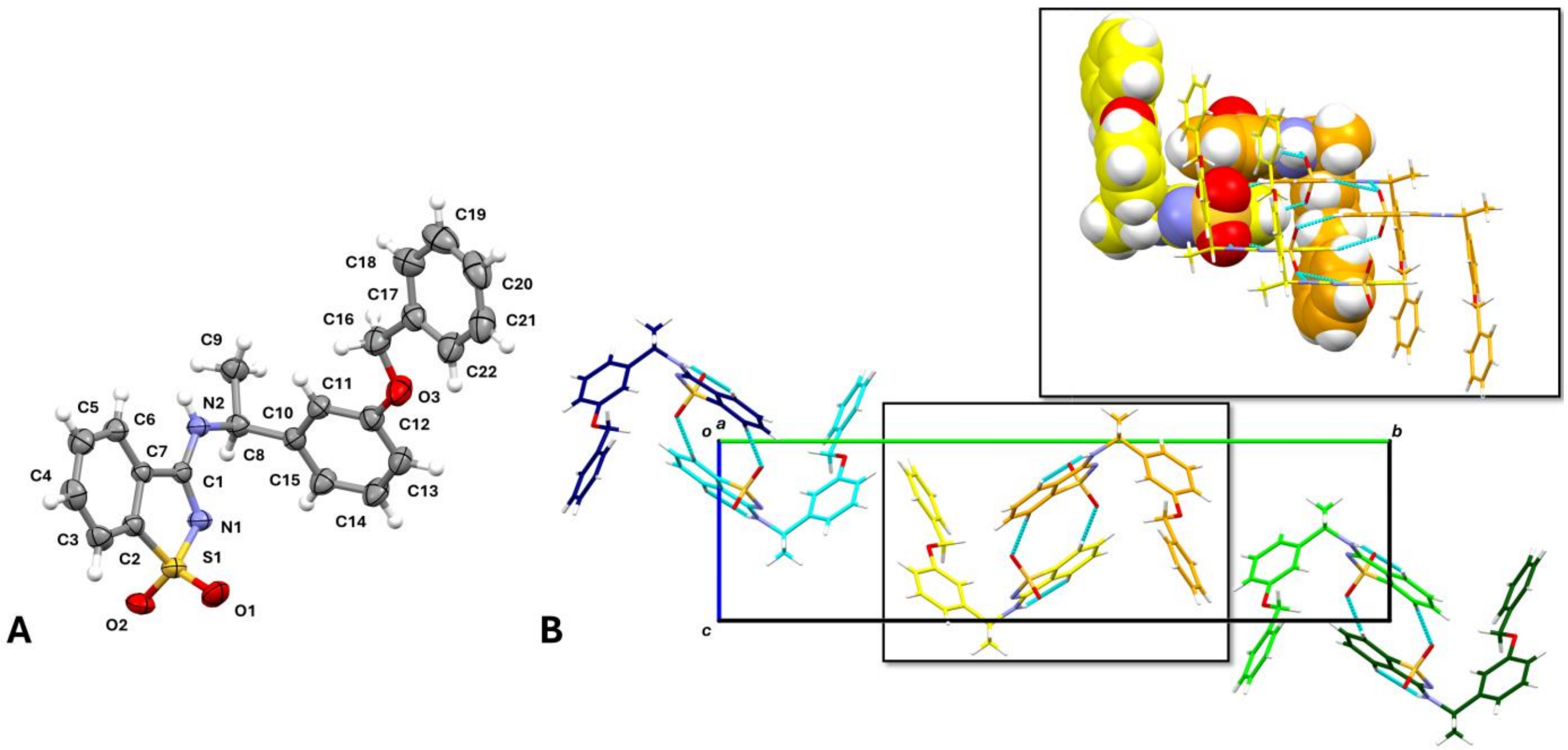

Figure S5. A. Thermal-ellipsoid diagram of **6c**, with an arbitrary atom-numbering scheme. Displacement ellipsoids are set at the 40% probability level. **B.** Stick model showing the main hydrogen bonds along the *a* axis. The different colors facilitate the identification of the double-strand chains propagating along the *a* axis. In the square, focus on the central chain with a stick/spacefill model showing the main hydrogen bonds and π-π stacking interactions.

Overall, the HS of **6c** shows similar features to that of **6a** (HS data: V = 482.50 Å^3, A = 428.59 Å^2, *G* = 0.694, Ω = 0.157). The d_{norm} surface presents red regions associated with the short-range hydrogen bonds involving the oxygen atoms of the SO_2 group (**Figure S6A**). The corresponding 2D plot is generally more compact, while retaining a very similar shape due to the conservation of the interaction types (**Figure S6B**). The curvedness property mapped onto the HS is particularly informative, as it

reveals a single flat region associated with the benzisothiazole sulfone moiety, indicative of a propensity for stacking interactions (**Figure S6C**). Notably, despite the presence of additional aryl groups in **6c**, no further flat regions are observed, accounting for the absence of significant π–π contacts. The shape-index mapping likewise corroborates the weakness of C–H···π interactions (**Figure S6D**). The enrichment ratios indicate that C···H/H···C, O···H/H···O, and C···C contacts are enriched. Finally, the CE-B3LYP interaction-energy analysis reveals several strongly stabilizing contacts, with total interaction energies reaching up to −75.4 and −90.8 kJ/mol for the most significant pairs (**Figure S6E** and **S6F**). These interactions are characterized by large attractive electrostatic and dispersion components, partially offset by repulsion, indicating a mixed stabilization regime. Additional contacts exhibit moderate stabilization, contributing to the overall lattice cohesion.

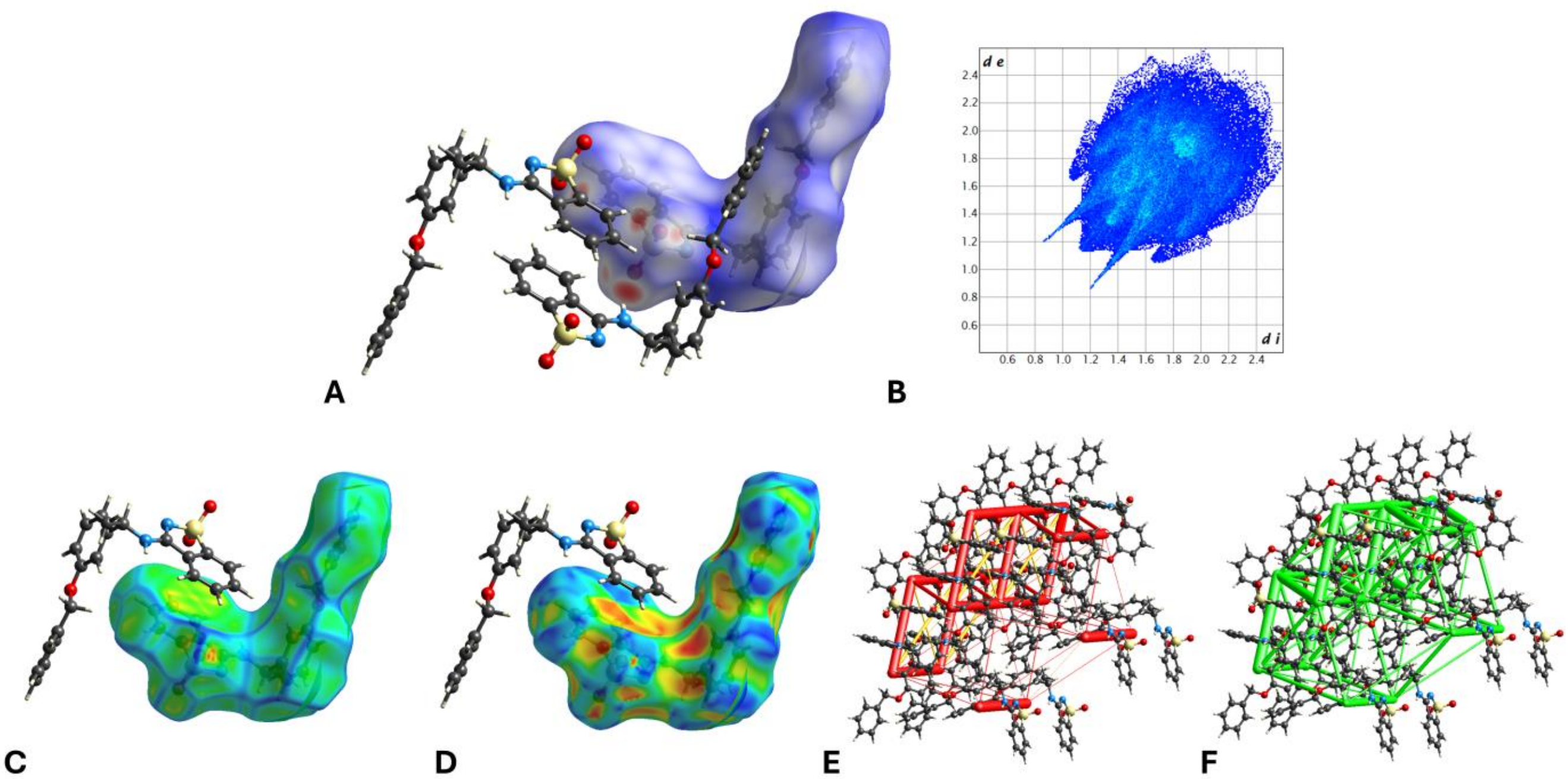

Figure S6. A. Three-dimensional representation of d_{norm} mapped onto the HS of **6c** (red-white-blue color scale: −0.3988 to 1.3927 a.u.). **B.** Two-dimensional Hirshfeld fingerprint plot (d_e vs d_i) derived from the d_{norm} surface. **C.** Three-dimensional representation of the curvedness property mapped onto the HS of **6c** (green-to-blue color scale: −3.7197 to 0.3563 a.u.). **D.** Three-dimensional representation of the shape index property mapped onto the HS of **6c** (red-to-blue color scale: −0.9972 to 0.9998 a.u.). **E-F.** Coulomb (**E**) and dispersion interaction energies (**F**) visualized as red and green cylinders, respectively, connecting molecular centroids and scaled to the interaction energy magnitude.

2.1 Compound Crystallography Experimental Procedures

Single crystals of **6a and 6c** were obtained as colorless plates from the slow evaporation of methanol solutions. X-ray diffraction data were collected on a Rigaku XtaLAB Synergy-S diffractometer (Rigaku, Tokyo, Japan) equipped with a microfocus Mo Kα source (λ = 0.71073 Å) and a HyPix-6000HE detector. Measurements were performed over 2θ = 2-25° at 50 kV and 1 mA using ω scans with a step width of

0.5° and an exposure time of 8 s and 25 s per frame for **6a** and **6c**, respectively. For the former, unit-cell parameters were refined from 9417 reflections in the range 2θ = 2-29°, revealing a metrically monoclinic lattice with space group I2. For the latter, 59,299 reflections were used in the same 2θ range, giving a monoclinic lattice with space group $P2_1/c$. Data reduction and absorption and Lorentz-polarization corrections were carried out using CrysAlisPro (v. 1.171.43.141a, Rigaku). The structures were solved by direct methods with SIR-2019/3[4] and refined using SHELXL-2025/1[5] within the WinGX suite (v. 2023.1).[6] Hydrogen atoms were introduced at idealized positions and treated using a riding model, with isotropic displacement parameters set to 1.2 U_{eq} of the corresponding parent atoms for aromatic, methylene, and methine groups, and 1.5 U_{eq} for methyl groups. Heteroatom-bound hydrogen atoms were refined freely. Geometrical parameters were analyzed using PARST[7] and Mercury (v. 2025.3.2),[8] while molecular illustrations were prepared with Mercury. Hirshfeld surface and energy framework calculations were performed with CrystalExplorer 21 (v. 21.5).[9] Overall summary is provided in **Table S3**, and further information include crystallographic data for **3b** (**Table S4**), **3d** (**Table S5**), **1d** (**Table S6**), **8d** (**Table S7**), and **8b** (**Table S8**).

Table S3. Crystal data and structure refinement statistics for **6a** and **6c**.

Identification code	**6a**		**6c**	
Empirical formula	$C_{15}H_{14}N_2O_2S$		$C_{22}H_{20}N_2O_3S$	
Formula weight	286.34		392.46	
Temperature (K)	293(2)		293(2)	
Wavelength (Å)	0.71073		0.71073	
Crystal system	Monoclinic		Monoclinic	
Space group	I2		$P2_1/c$	
Unit cell dimensions (Å/°)	a = 7.9791(2)	α = 90	a = 7.23220(10)	α = 90
	b = 6.96800(10)	β = 98.080(2)	b = 31.8797(3)	β = 102.4740(10)
	c = 26.2713(6)	γ = 90	c = 8.72660(10)	γ = 90
Volume ($Å^3$)	1446.14(5)		1964.51(4)	
Z	4		4	
Density calcd. (Mg/m^3)	1.315		1.327	
Abs. coefficient (mm^{-1})	0.226		0.190	
F(000)	600		824	
Crystal size (mm^3)	0.35 x 0.25 x 0.05		0.20 x 0.10 x 0.03	
θ range data collection (°)	1.566 to 25.350		2.474 to 25.348	
Index ranges	$-9 \le h \le 9$, $-8 \le k \le 8$, $-31 \le l \le 31$		$-8 \le h \le 8$, $-38 \le k \le 38$, $-10 \le l \le 10$	
Reflections collected	13,761		118,431	
Independent reflections	2652 [R_{int} = 0.0193]		3597 [R_{int} = 0.0449]	
Completeness to θ_{max} (%)	100.0		99.9	
Refinement method	Full-matrix least-squares on F^2		Full-matrix least-squares on F^2	
Data/restraints/parameters	2652/1/186		3597/0/258	
Goodness-of-fit on F^2	1.056		1.070	
Final R indices [$I>2\sigma(I)$]	R1 = 0.0276, wR2 = 0.0758		R1 = 0.0409, wR2 = 0.1126	
R indices (all data)	R1 = 0.0295, wR2 = 0.0771		R1 = 0.0455, wR2 = 0.1163	
Flack parameter (Parsons)	-0.03(2)		/	
Largest diff. peak/hole ($e \cdot Å^{-3}$)	0.105 and -0.151		0.334 and -0.345	
CCDC deposition number	2528438		2528439	

2.2 *N*-(1-(Naphthalen-2-yl)ethyl)formamide (3b)

Single crystals were obtained as colorless plates from the slow evaporation of a DCM/PE mixture. X-ray diffraction data were collected on a Rigaku XtaLAB Synergy-S diffractometer (Rigaku, Tokyo, Japan) equipped with a microfocus Mo Kα source (λ = 0.71073 Å) and a HyPix-6000HE detector. Measurements were performed over 2θ = 2-25° at 50 kV and 1 mA using ω scans with a step width of 0.5° and an exposure time of 70 s per frame. Unit-cell parameters were refined from 7220 reflections in the range 2θ = 2-29°, revealing a metrically monoclinic lattice with space group $P2_1/c$. Data reduction and absorption and Lorentz-polarization corrections were carried out using CrysAlisPro (v. 1.171.43.141a, Rigaku). The structure was solved by direct methods with SIR-2019/3[4] and refined using SHELXL-2025/1[5] within the WinGX suite (v. 2023.1).[6]

Table S4. Crystal data and structure refinement for **3b**.

Identification code	**3b**		**Thermal ellipsoid diagram (40% probability)**
Empirical formula	$C_{13}H_{13}NO$		
Formula weight	199.24		
Temperature (K)	293(2)		
Wavelength (Å)	0.71073		
Crystal system	Monoclinic		
Space group	$P2_1/c$		
Unit cell dimensions (Å/°)	a = 10.8176(6)	α = 90	
	b = 10.2219(5)	β = 100.389(4)	
	c = 10.0862(4)	γ = 90	
Volume ($Å^3$)	1097.01(9)		
Z	4		
Density calcd. (Mg/m^3)	1.206		
Abs. coefficient (mm^{-1})	0.076		
F(000)	424		
Crystal size (mm^3)	0.20 x 0.10 x 0.03		
θ range data collection (°)	1.914 to 25.343		
Index ranges	$-13 \le h \le 13$, $-12 \le k \le 12$, $-12 \le l \le 12$		
Reflections collected	17,066		
Independent reflections	2004 [R_{int} = 0.0440]		
Completeness to θ_{max} (%)	100.0		
Refinement method	Full-matrix least-squares on F^2		
Data/restraints/parameters	2004/0/141		
Goodness-of-fit on F^2	1.038		
Final R indices [$I>2\sigma(I)$]	R1 = 0.0413, wR2 = 0.1030		
R indices (all data)	R1 = 0.0630, wR2 = 0.1137		
Largest diff. peak/hole ($e\cdot Å^{-3}$)	0.112 and -0.147		
CCDC deposition number	2527969		

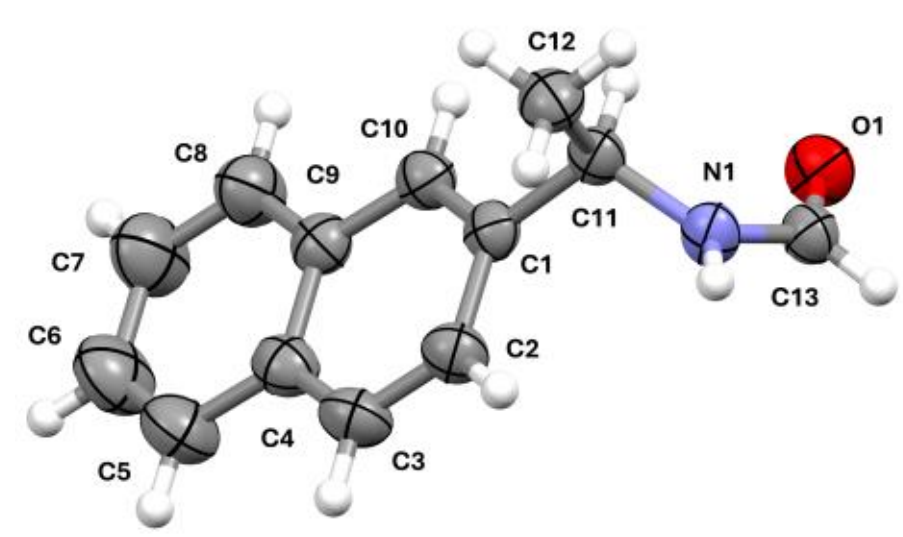

2.3 (*R*)-*N*-(1-(2-Chloro-4-(4-chlorophenoxy)phenyl)ethyl)formamide (3d)

Single crystals were obtained as colorless needles from the slow evaporation of an acetone/MeOH mixture. X-ray diffraction data were collected on a Rigaku XtaLAB Synergy-S diffractometer (Rigaku, Tokyo, Japan) equipped with a microfocus Mo Kα source (λ = 0.71073 Å) and a HyPix-6000HE detector. Measurements were performed over 2θ = 2-25° at 50 kV and 1 mA using ω scans with a step width of 0.5° and an exposure time of 10 s per frame. Unit-cell parameters were refined from 38,507 reflections in the range 2θ = 2-25°, revealing a metrically orthorhombic lattice with space group $P2_1 2_1 2_1$. Data reduction and absorption and Lorentz-polarization corrections were carried out using CrysAlisPro (v. 1.171.43.141a, Rigaku). The structure was solved by direct methods with SIR-2019/3[4] and refined using SHELXL-2025/1[5] within the WinGX suite (v. 2023.1).[6] The absolute configuration at the chiral center (C7) was assigned as (*R*) based on the Flack parameter.

Table S5. Crystal data and structure refinement for **3d**.

Identification code	**3d**	**Thermal ellipsoid diagram (40% probability)**
Empirical formula	$C_{15}H_{13}Cl_2NO_2$	
Formula weight	310.16	
Temperature (K)	293(2)	
Wavelength (Å)	0.71073	
Crystal system	Orthorhombic	
Space group	$P2_12_12_1$	
Unit cell dimensions (Å/°)	*a* = 5.01180(10) α = 90	
	b = 12.0932(2) β = 90	
	c = 24.2655(3) γ = 90	
Volume (Å^3)	1470.70(4)	
Z	4	
Density calcd. (Mg/m^3)	1.401	
Abs. coefficient (mm^{-1})	0.441	
F(000)	640	
Crystal size (mm^3)	0.50 x 0.10 x 0.03	
θ range data collection (°)	1.678 to 25.325	
Index ranges	$-6 \leq h \leq 6$, $-14 \leq k \leq 14$, $-29 \leq l \leq 29$	
Reflections collected	89,373	
Independent reflections	2676 [R_{int} = 0.0451]	
Completeness to θ_{max} (%)	100.0	
Refinement method	Full-matrix least-squares on F^2	
Data/restraints/parameters	2676/1/186	
Goodness-of-fit on F^2	1.044	
Final R indices [I>2σ(I)]	R1 = 0.0279, wR2 = 0.0728	
R indices (all data)	R1 = 0.0308, wR2 = 0.0751	
Flack parameter (Parsons)	-0.013(16)	
Largest diff. peak/hole (e·Å^{-3})	0.224 and -0.227	
CCDC deposition number	2528324	

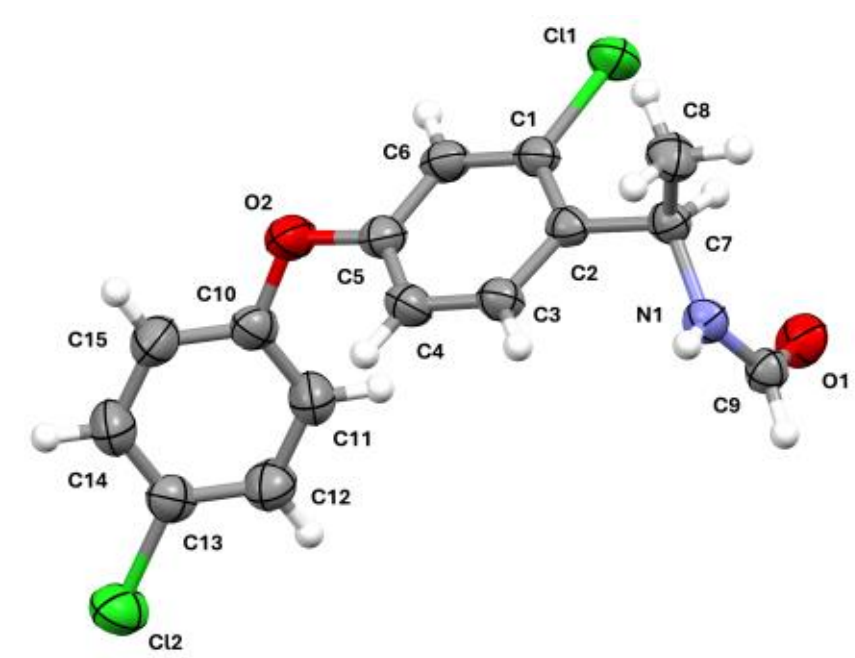

2.4 (*S*)-1-(2-Chloro-4-(4-chlorophenoxy)phenyl)ethan-1-amine hydrochloride (1d)

Single crystals were obtained as colorless plates from the slow evaporation of an acetone/MeOH/MeCN mixture. X-ray diffraction data were collected on a Rigaku XtaLAB Synergy-S diffractometer (Rigaku, Tokyo, Japan) equipped with a microfocus Mo Kα source (λ = 0.71073 Å) and a HyPix-6000HE detector. Measurements were performed over 2θ = 2-25° at 50 kV and 1 mA using ω scans with a step width of 0.5° and an exposure time of 5.3 s per frame. Unit-cell parameters were refined from 22,972 reflections in the range 2θ = 2-28°, revealing a metrically orthorhombic lattice with space group $P2_1\ 2_1\ 2_1$. Data reduction and absorption and Lorentz-polarization corrections were carried out using CrysAlisPro (v. 1.171.43.141a, Rigaku). The structure was solved by direct methods with SIR-2019/3[4] and refined using SHELXL-2025/1[5] within the WinGX suite (v. 2023.1).[6] The *p*-chlorophenyl ring is disordered over two positions with equal occupancy. The absolute configuration at the chiral center (C7) was assigned as (*S*) based on the Flack parameter.

Table S6. Crystal data and structure refinement for **1d**.

Identification code	**1d**	
Empirical formula	$C_{14}H_{14}Cl_3NO$	
Formula weight	318.61	
Temperature (K)	293(2)	
Wavelength (Å)	0.71073	
Crystal system	Orthorhombic	
Space group	$P2_12_12_1$	
Unit cell dimensions (Å/°)	a = 5.33230(10)	α = 90
	b = 7.2329(2)	β = 90
	c = 39.4256(8)	γ = 90
Volume (Å^3)	1520.57(6)	
Z	4	
Density calcd. (Mg/m^3)	1.392	
Abs. coefficient (mm^{-1})	0.593	
F(000)	656	
Crystal size (mm^3)	0.25 x 0.15 x 0.02	
θ range data collection (°)	2.066 to 25.348	
Index ranges	$-6 \le h \le 6$, $-8 \le k \le 8$, $-47 \le l \le 47$	
Reflections collected	46,150	
Independent reflections	2794 [R_{int} = 0.0624]	
Completeness to θ_{max} (%)	99.9	
Refinement method	Full-matrix least-squares on F^2	
Data/restraints/parameters	2794/93/221	
Goodness-of-fit on F^2	1.068	
Final R indices [I>2σ(I)]	R1 = 0.0399, wR2 = 0.1127	
R indices (all data)	R1 = 0.0417, wR2 = 0.1146	
Flack parameter (Parsons)	-0.01(2)	
Largest diff. peak/hole (e·Å^{-3})	0.273 and -0.288	
CCDC deposition number	2528325	

Thermal ellipsoid diagram (40% probability)

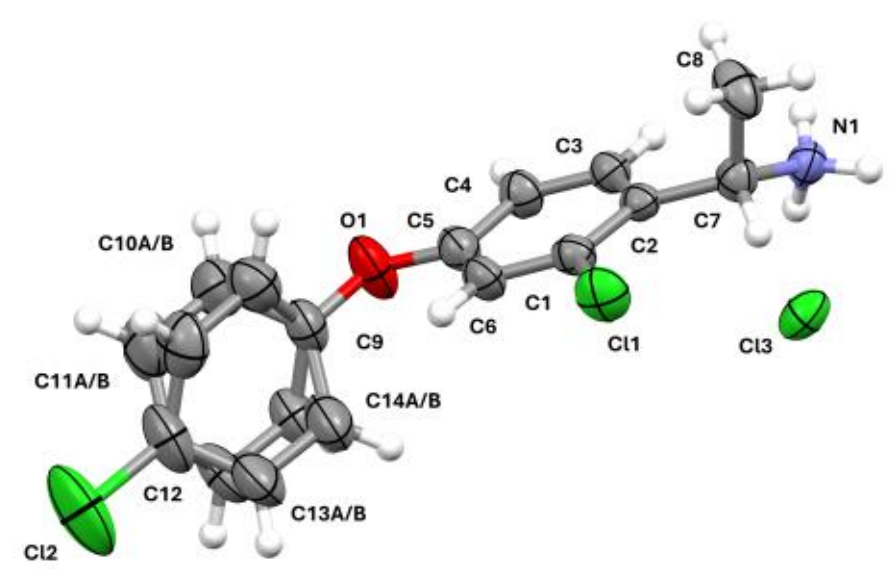

2.5 3-((1-(2-Chloro-4-(4-chlorophenoxy)phenyl)ethyl)amino)benzo[*d*]isothiazole 1,1-dioxide (6d)

Single crystals were obtained as colorless needles from the slow evaporation of a MeOH solution. X-ray diffraction data were collected on a Rigaku XtaLAB Synergy-S diffractometer (Rigaku, Tokyo, Japan) equipped with a microfocus Mo Kα source (λ = 0.71073 Å) and a HyPix-6000HE detector. Measurements were performed over 2θ = 2-25° at 50 kV and 1 mA using ω scans with a step width of 0.5° and an exposure time of 8.5 s per frame. Unit-cell parameters were refined from 108,506 reflections in the range 2θ = 2-29°, revealing a metrically orthorhombic lattice with space group $Pna2_1$. Data reduction and absorption and Lorentz-polarization corrections were carried out using CrysAlisPro (v. 1.171.43.141a, Rigaku). The structure was solved by direct methods with SIR-2019/3[4] and refined using SHELXL-2025/1[5] within the WinGX suite (v. 2023.1).[6] The structure was refined as a two-component inversion twin. The refined Flack and BASF parameters indicate nearly equal twin-domain volumes, consistent with a racemic crystal. The R and goodness-of-fit (GOOF) values remained relatively high upon refinement, likely due to residual Fourier-map artifacts associated with inversion twinning.

Table S7. Crystal data and structure refinement for **6d**.

Identification code	**6d**		**Thermal ellipsoid diagram (40% probability)**
Empirical formula (ASU)	$C_{21}H_{16}Cl_2N_2O_3S$		
Formula weight (ASU)	447.32		
Temperature (K)	293(2)		
Wavelength (Å)	0.71073		
Crystal system	Orthorhombic		
Space group	$Pna2_1$		
Unit cell dimensions (Å/°)	a = 22.5303(2)	α = 90	
	b = 7.00110(10)	β = 90	
	c = 25.4186(3)	γ = 90	
Volume ($Å^3$)	4009.45(8)		
Z	8		
Density calcd. (Mg/m^3)	1.482		
Abs. coefficient (mm^{-1})	0.454		
F(000)	1840		
Crystal size (mm^3)	0.30 x 0.08 x 0.02		
θ range data collection (°)	1.602 to 24.712		
Index ranges	$-26 \le h \le 26$, $-8 \le k \le 8$, $-29 \le l \le 29$		
Reflections collected	164,757		
Independent reflections	6828 [R_{int} = 0.0526]		
Completeness to θ_{max} (%)	99.8		
Refinement method	Full-matrix least-squares on F^2		
Data/restraints/parameters	6828/1/524		
Goodness-of-fit on F^2	1.118		
Final R indices [$I>2\sigma(I)$]	R1 = 0.0979, wR2 = 0.2211		
R indices (all data)	R1 = 0.0999, wR2 = 0.2256		
Flack parameter (Parsons)	0.43(13)		
Largest diff. peak/hole ($e{\cdot}Å^{-3}$)	2.458 and -0.420		
CCDC deposition number	2528702		

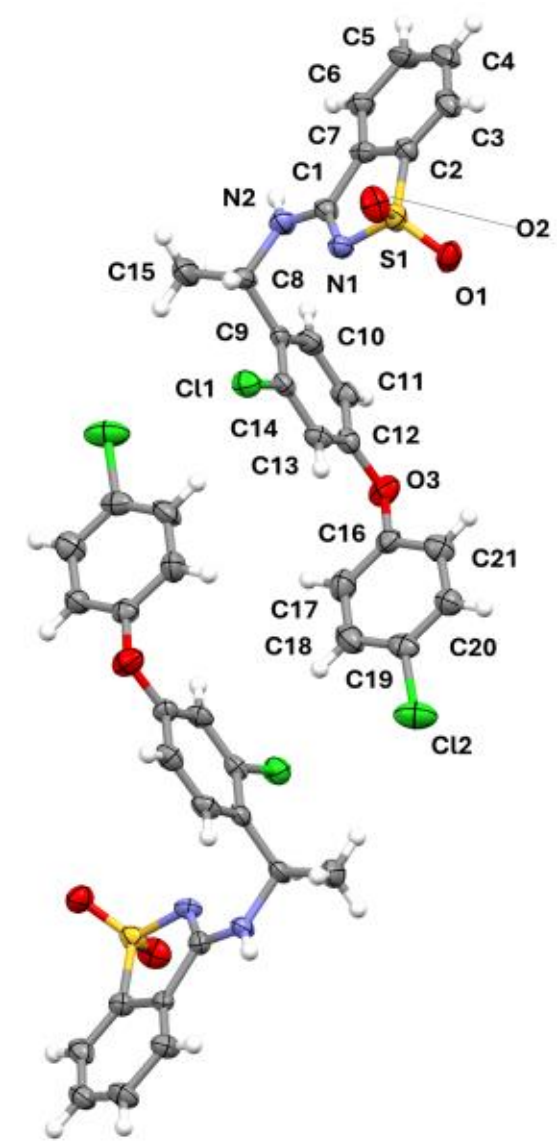

2.6 3-((1-(Naphthalen-2-yl)ethyl)amino)benzo[*d*]isothiazole 1,1-dioxide (6b)

Single crystals were obtained as colorless plates from the slow evaporation of an acetone/MeOH/MeCN mixture. X-ray diffraction data were collected on a Rigaku XtaLAB Synergy-S diffractometer (Rigaku, Tokyo, Japan) equipped with a microfocus Mo Kα source (λ = 0.71073 Å) and a HyPix-6000HE detector. Measurements were performed over 2θ = 2-25° at 50 kV and 1 mA using ω scans with a step width of 0.5° and an exposure time of 28.9 s per frame. Unit-cell parameters were refined from 36,567 reflections in the range 2θ = 3-34°, revealing a metrically monoclinic lattice with space group C2/c. Data reduction and absorption and Lorentz-polarization corrections were carried out using CrysAlisPro (v. 1.171.43.141a, Rigaku). The structure was solved by direct methods with SIR-2019/3[4] and refined using SHELXL-2025/1[5] within the WinGX suite (v. 2023.1).[6] The structure was refined as a two-component non-merohedral twin (BASF = 0.29), based on an *hklf5* file. The slightly elevated R_{int} value can be ascribed to the non-merohedral twinning of the crystal.

Table S8. Crystal data and structure refinement for **6b**.

Identification code	**6b**		**Thermal ellipsoid diagram (40% probability)**
Empirical formula	$C_{19}H_{16}N_2O_2S$		
Formula weight	336.40		
Temperature (K)	293(2)		
Wavelength (Å)	0.71073		
Crystal system	Monoclinic		
Space group	C2/c		
Unit cell dimensions (Å/°)	*a* = 33.2563(8)	*α* = 90	
	b = 7.07040(10)	*β* = 98.671(2)	
	c = 14.2909(4)	*γ* = 90	
Volume (Å^3)	3321.89(13)		
Z	8		
Density calcd. (Mg/m^3)	1.345		
Abs. coefficient (mm^{-1})	0.208		
F(000)	1408		
Crystal size (mm^3)	0.25 x 0.15 x 0.05		
θ range data collection (°)	2.884 to 24.708		
Index ranges	$-39 \le h \le 38$, $-8 \le k \le 8$, $-16 \le l \le 16$		
Reflections collected	392,903		
Independent reflections	4818 [R_{int} = 0.1543]		
Completeness to θ_{max} (%)	99.9		
Refinement method	Full-matrix least-squares on F^2		
Data/restraints/parameters	4818/0/218		
Goodness-of-fit on F^2	1.099		
Final R indices [I>2σ(I)]	R1 = 0.0528, wR2 = 0.1480		
R indices (all data)	R1 = 0.0604, wR2 = 0.1534		
Largest diff. peak/hole (e·Å^{-3})	0.181 and -0.251		
CCDC deposition number	2528437		

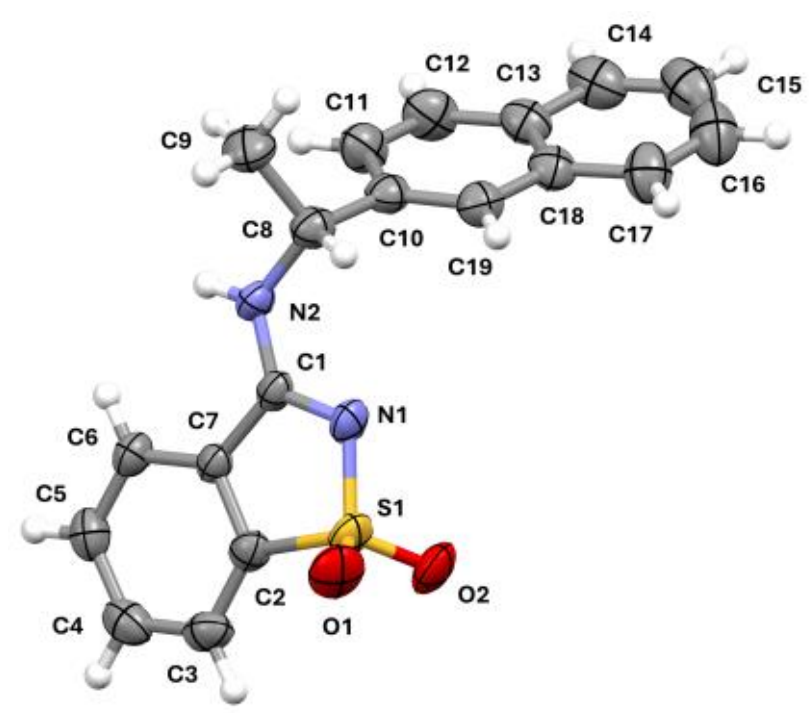

2.7 (*S*)-3-((1-Phenylethyl)amino)benzo[*d*]isothiazole 1,1-dioxide (6a)

Table S9. Hirshfeld surface analysis of **6a** showing the intermolecular contact network. Contact contributions were computed using *CrystalExplorer*, and enrichment ratios were determined according to the methodology of Jelsch *et al.*[10]

**Atoms**	**S**	**N**	**H**	**C**	**O**
Surface (%)	0.1	2.7	66.8	16.3	14.3
Contacts (%)					
S	0.0				
N	0.0	0.0			
H	0.1	5.0	41.5		
C	0.0	0.3	18.6	6.3	
O	0.3	0.0	26.9	1.0	0.3
Enrichments					
S	-				
N	-	-			
H	-	**1.41**	0.93		
C	-	-	0.86	**2.39**	
O	0.1	0.8	**1.41**	0.2	0.1

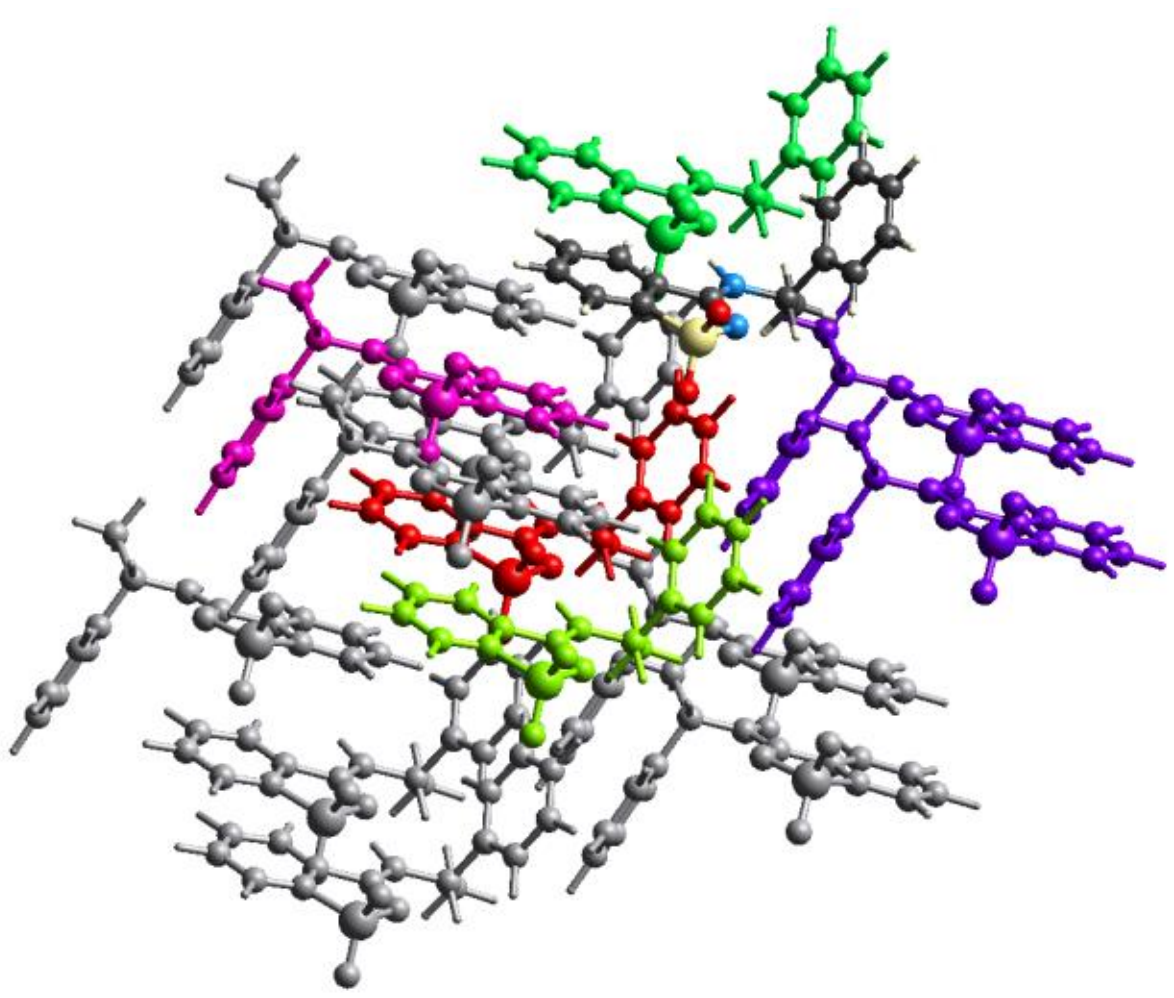

Interaction Energies (kJ/mol)
R is the distance between molecular centroids (mean atomic position) in Å.

Total energies, only reported for two benchmarked energy models, are the sum of the four energy components, scaled appropriately (see the scale factor table below)

	N	Symop	R	Electron Density	E_ele	E_pol	E_dis	E_rep	E_tot
	2	x, y, z	7.98	B3LYP/6-31G(d,p)	4.8	-3.1	-22.8	13.3	-8.9
	2	-x+1/2, y+1/2, -z+1/2	10.54	B3LYP/6-31G(d,p)	-0.2	-0.6	-17.0	7.9	-10.6
	2	x, y, z	10.59	B3LYP/6-31G(d,p)	-6.4	-2.9	-4.7	5.5	-9.5
	2	x, y, z	6.97	B3LYP/6-31G(d,p)	-48.3	-15.8	-29.9	50.6	-57.6
	1	-x, y, -z	5.44	B3LYP/6-31G(d,p)	-8.7	-9.1	-70.6	43.0	-51.0
	2	-x, y, -z	8.84	B3LYP/6-31G(d,p)	-3.9	-2.1	-5.0	0.2	-9.9
	2	-x+1/2, y+1/2, -z+1/2	8.50	B3LYP/6-31G(d,p)	2.0	-1.6	-11.8	5.4	-6.0
	1	-x, y, -z	9.16	B3LYP/6-31G(d,p)	-4.1	-2.9	-22.0	6.1	-21.9

Scale factors for benchmarked energy models
See Mackenzie et al. IUCrJ (2017)

Energy Model	k_ele	k_pol	k_disp	k_rep
CE-HF ... HF/3-21G electron densities	1.019	0.651	0.901	0.811
CE-B3LYP ... B3LYP/6-31G(d,p) electron densities	1.057	0.740	0.871	0.618

Figure S7. Results of the energy framework analysis, performed on **6a** with *CrystalExplorer*.

2.8 3-((1-(3-(Benzyloxy)phenyl)ethyl)amino)benzo[*d*]isothiazole 1,1-dioxide (6c)

Table S10. Hirshfeld surface analysis of **6c** showing the intermolecular contact network. Contact contributions were computed using *CrystalExplorer*, and enrichment ratios were determined according to the methodology of Jelsch *et al.* [10]

**Atoms**	**S**	**N**	**H**	**C**	**O**
Surface (%)	0.0	1.9	70.5	16.6	11.1
Contacts (%)					
S	0.0				
N	0.0	0.0			
H	0.0	2.0	46.6		
C	0.0	1.5	24.2	3.6	
O	0.0	0.2	21.6	0.2	0.1
Enrichments					
S	-				
N	-	-			
H	-	0.77	0.94		
C	-	-	**1.04**	**1.31**	
O	-	-	**1.38**	0.1	0.1

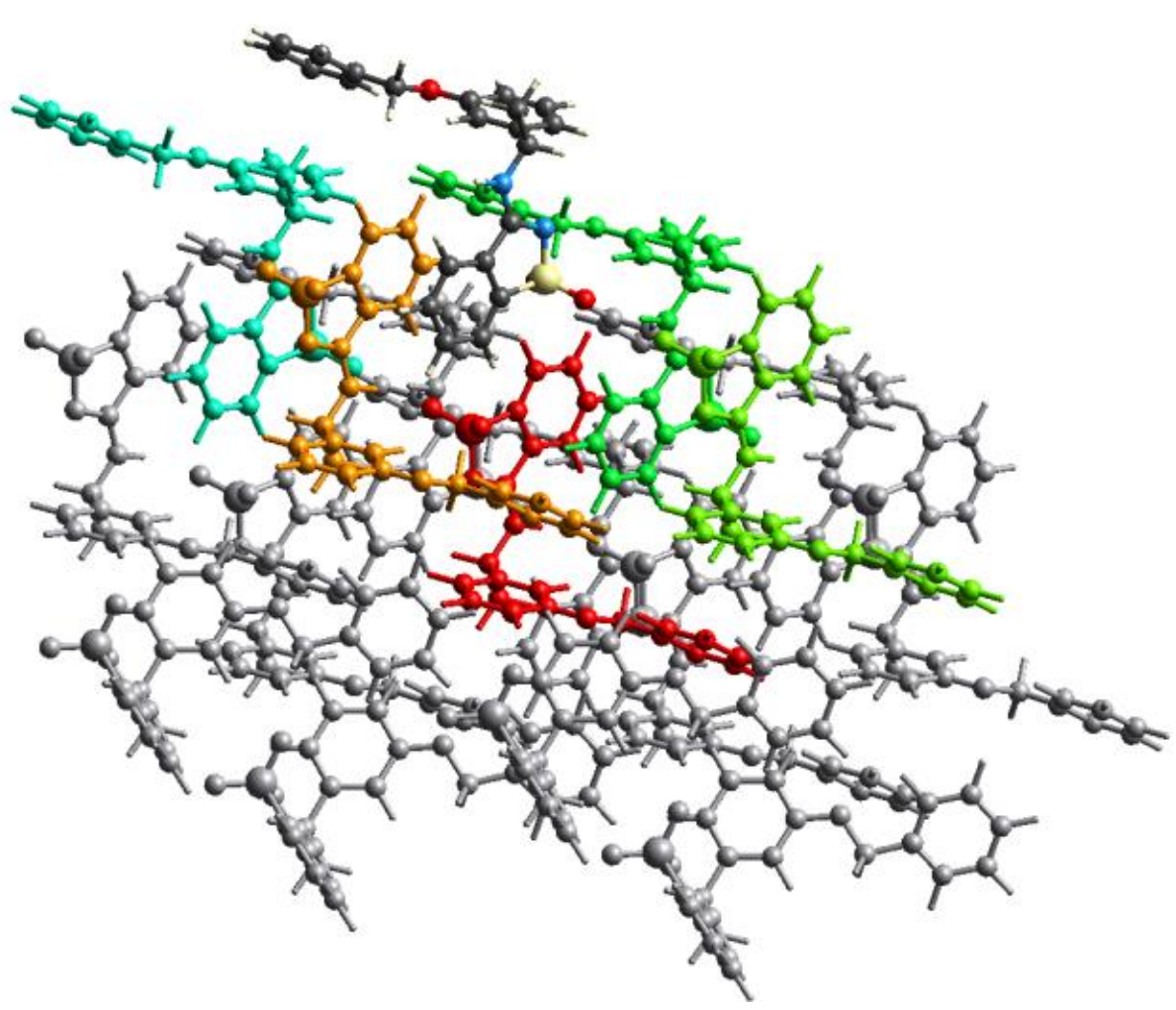

Interaction Energies (kJ/mol)
R is the distance between molecular centroids (mean atomic position) in Å.

Total energies, only reported for two benchmarked energy models, are the sum of the four energy components, scaled appropriately (see the scale factor table below)

	N	Symop	R	Electron Density	E_ele	E_pol	E_dis	E_rep	E_tot
	1	-x, -y, -z	11.31	B3LYP/6-31G(d,p)	-32.1	-8.6	-13.9	25.9	-36.4
	1	-x, -y, -z	7.21	B3LYP/6-31G(d,p)	-17.5	-7.3	-50.9	21.8	-54.7
	1	-x, -y, -z	8.92	B3LYP/6-31G(d,p)	-43.5	-14.9	-62.5	33.4	-90.8
	1	-x, -y, -z	13.52	B3LYP/6-31G(d,p)	15.6	-2.5	-2.9	0.0	12.2
	2	x, y, z	7.23	B3LYP/6-31G(d,p)	-49.0	-17.5	-45.3	46.6	-75.4
	2	x, y, z	8.73	B3LYP/6-31G(d,p)	1.2	-1.5	-22.9	10.7	-13.2
	2	x, -y+1/2, z+1/2	10.63	B3LYP/6-31G(d,p)	-3.0	-0.6	-22.8	10.6	-17.0
	2	x, -y+1/2, z+1/2	13.38	B3LYP/6-31G(d,p)	-5.0	-0.4	-9.7	0.0	-14.1
	2	x, y, z	12.48	B3LYP/6-31G(d,p)	-2.8	-0.7	-11.8	0.0	-13.7
	1	-x, -y, -z	8.98	B3LYP/6-31G(d,p)	0.2	-1.8	-17.4	7.5	-11.7
	1	-x, -y, -z	9.00	B3LYP/6-31G(d,p)	8.3	-3.3	-20.1	12.8	-3.2

Scale factors for benchmarked energy models
See Mackenzie et al. IUCrJ (2017)

Energy Model	k_ele	k_pol	k_disp	k_rep
CE-HF ... HF/3-21G electron densities	1.019	0.651	0.901	0.811
CE-B3LYP ... B3LYP/6-31G(d,p) electron densities	1.057	0.740	0.871	0.618

Figure S8. Results of the energy framework analysis, performed on **6c** with *CrystalExplorer*.

3. BIOLOGY

3.1 Protein Expression and Purification

The coding sequence corresponding to the catalytic region of human SOS1 (UniProt Q07889, residues 564-1049) codon optimized for *Escherichia coli (E. coli)* cloned into the pETM-11 expression vector (EMBL) using NcoI and HindIII (NEB). The resulting plasmid was transformed into *E. coli* BL21 Star (DE3) pLysS and a single colony was used to inoculate 10 mL of Luria-Bertani (LB) media supplemented with 50 mg/mL kanamycin and 35 mg/mL chloramphenicol and grown for 16 hours at 37 °C and 180 rpm. Thereafter, 1 mL of the preculture was used to inoculate 1 L of LB media (supplemented with 50 mg/mL kanamycin and 35 mg/mL chloramphenicol). To express recombinant SOS1 with a C-terminal 6His-tag, protein expression was induced by the addition of 0.5 mM isopropyl-1-β-d-thiogalactopyranoside (IPTG; abcr chemicals) when the culture OD600 reached 0.6. The culture was subsequently maintained at 18 °C in a shaking incubator (180 rpm) and, after an overnight incubation, the bacteria were collected by centrifugation at 4000g for 20 min. Lysis buffer (50 mM Tris-HCl, pH 8, 300 mM NaCl, 10% glycerol, 3 mM β-mercaptoethanol, and 25 mM imidazole, 20 µg/ml DNAse 1, 0.4 mg/ml lysozyme) was used to resuspend the cell pellet, and bacteria were lysed by sonication (50% amplitude, 2 sec on/15 sec off, for 5 min). The lysate was subsequently clarified by centrifugation at 50 000g for 45 min. The cleared cell-lysate was loaded on a 5 ml HiTrap HP column (GE Healthcare), washed with 5 column volumes of Buffer A (50 mM Tris-HCl, pH 8, 300 mM NaCl, 10% glycerol, and 3 mM β-mercaptoethanol) and subsequently eluted in Buffer B (50 mM Tris-HCl, pH 8, 300 mM NaCl, 10% glycerol, 3 mM β-mercaptoethanol, and 500 mM imidazole). His-tagged TEV protease was added to the elution (1:10 w/w), and the mixture was dialysed overnight at 4 °C against 50 mM TrisHCl, 50 mM NaCl, 10 % glycerol, and 3 mM BME to remove the imidazole and cleave the 6xHis tag from the SOS1 protein. Reverse HiTrap purification was used to elute the untagged SOS1 protein, which was further purified by size-exclusion chromatography (SEC) on a HiLoad 16/60 S200 (GE Healthcare) column equilibrated with SEC buffer (20 mM HEPES, pH 7.5, 50 mM NaCl). Finally, the purified SOS1 was concentrated to 10 mg ml^{-1} using a Vivaspin centrifugal concentrator (molecular weight cut-off (MWCO) 10 kDa, Sartorius). KRAS WT (1-169) was expressed and purified as previously described.[11]

3.2 SOS1 (co-)crystallization

Apo crystals were obtained using the sitting-drop vapour diffusion method at 18 ºC, with drops containing 0.5 µL protein solution (10 mg ml^{-1}) and 0.5 µL reservoir solution (20% ethylene glycol). To obtain co-crystals of SOS1 with the SOS1 binders, compounds (**6b** and **6e**) were dissolved in DMSO to a final concentration of 100 mM. Binding was performed overnight at 4 ºC at a 5:1 molar ratio of compound to SOS1 protein. After overnight binding, samples were centrifuged for 10 min at 20.000g to remove any aggregates and precipitates. Subsequently, the cleared and pre-incubated protein solutions were used

to obtain co-crystals by the sitting-drop vapour-diffusion method at 18 ºC. As with the apo crystals, drops contained 0.5 µL of protein solution (10 mg ml^{-1}) and 0.5 µL of reservoir solution (20% ethylene glycol). Data from X-ray diffraction were collected at 100 K on beamline P11 of the Deutsches Elektronen-Synchrotron (DESY) (Hamburg, Germany). Data integration and scaling were performed using XDS[12] and Aimless[13] from the CCP4 software.[14] The DIMPLE software in CCP4 was used to obtain the solved structures, utilising a previously solved SOS1 structure (without ligand) as the reference model (PDB: 5OVD). The DIMPLE output models were then subjected to iterative cycles of model building with COOT and refinement with REFMAC.[14,15] The structures were deposited into the PDB (30JO and 30JN for **6e** and **6b**, respectively). Statistics for both data collection and refinement are presented in **Table S2**.

3.3 Thermal Shift Assay

Initial screening for SOS1 binders was performed against a small-molecule library. Each reaction consisted of 1 µL of compound (5 mM or 10 mM stock solutions in 100% DMSO) mixed with 49 µL of master mix containing 5000× SYPRO™ Orange (Thermo Fisher Scientific, S6650), 157 µM SOS1, and 50 mM NaCl in phosphate-buffered saline (PBS; Glentham Life Sciences, GX8368), pH 7.2. The final assay conditions were 5 µM protein, 2% (v/v) DMSO, and ligand concentrations of 100 µM or 200 µM. Control samples containing 2% (v/v) DMSO were included on each plate, along with blank wells containing no protein. Plates were centrifuged at 475 × g for 5 min at 25 °C using an Eppendorf 5810R centrifuge equipped with an S-4-104 swing-bucket rotor, prior to loading and sealed to prevent evaporation during the experiment. The assays were performed using a temperature ramp starting from 25 °C, with an initial incubation step of 30 min, followed by a continuous melt curve from 25 °C to 95 °C with temperature increments of 0.5 °C and a dwell time of 30 s per step, during which fluorescence was recorded at each increment. Fluorescence was monitored in the HEX channel (excitation λ =535-556 nm, emission λ =560-580 nm). The same settings were employed for the P1/P2 enantiomers. All measurements were performed in triplicate. Inflection points of the melting curves were determined using Bio-Rad CFX96 control software and used as indicators of sample thermal stability.

3.4 Microscale Thermophoresis

MST measurements were performed on a Nanotemper Monolith NT.115 instrument (Nanotemper Technologies GmbH). Purified SOS1 was labeled using the RED-NHS Monolith Protein Labelling Kit according to the manufacturer's protocol. The labeled protein was concentrated using a centrifugal filter with a 3 kDa cutoff, diluted with glycerol to a final concentration of 30% (v/v), aliquoted, and stored at −80 °C. MST measurements were performed in MST buffer (PBS pH 7.2 supplemented with 50 mM NaCl and 0.01% Pluronic® F-127) using premium capillaries (Nanotemper Technologies GmbH). Labeled SOS1 was used at a final concentration of 50 nM. Ligands were prepared from a 10 mM stock

solution in DMSO and titrated in a 1:1 serial dilution in PCR tubes according to the manufacturer's protocol, yielding 16 ligand concentrations with a highest final concentration of 200 µM and a constant final DMSO concentration of 2.0% (v/v) in all samples. All binding reactions were incubated for 30 min at room temperature and subsequently centrifuged at 21,300 × g for 5 min at 4 °C using an Eppendorf® FA-24×2 rotor prior to capillary loading. All measurements were performed in quadruplicate using 20% LED power and 40% MST power. MST data were analyzed using MO.Affinity Analysis software (NanoTemper Technologies GmbH) and the thermophoretic response was evaluated at an on-time of 2.5 s for all measurements.

3.5 Loading of KRAS with Nucleotides

KRAS (50-100 µM) was incubated with a 5-fold molar excess of BODIPY-GDP in nucleotide exchange buffer consisting of 20 mM HEPES (pH 7.5), 100 mM NaCl, 1 mM TCEP, 5% (v/v) glycerol, and 10 mM EDTA for 3h on ice. After incubation, the exchange reaction was terminated by addition of 20 mM $MgCl_2$, followed by incubation on ice for 5-10 min. Excess and unbound nucleotide were removed by buffer exchange in Amicon® Ultra centrifugal filter units (2 mL, 10 kDa MWCO; MilliporeSigma, Burlington, MA, USA) to remove any unbound nucleotide.

3.6 Nucleotide Exchange Assay

Nucleotide exchange assays were performed following a previously reported protocol with minor modifications.[16] Assays were conducted in black, clear-bottom 384-well plates (ViewPlate-384 F, Revvity; cat. no. 6007470) in a total reaction volume of 50 µL. The assay buffer consisted of 20 mM HEPES (pH 7.5), 50 mM NaCl, 2 mM $MgCl_2$, and 1 mM TCEP at 25 °C. All reactions contained 0.5 µM SOS1, 10 µM compound, and 0.2% DMSO (v/v). For inhibitor-treated samples, SOS1 was preincubated with the test compound or the reference inhibitor BI-3406 prior to initiation of the assay or DMSO which served as negative control. BODIPY-GDP dissociation was monitored using 0.5 µM KRAS WT·BODIPY-GDP and BODIPY-GTP loading was assessed using 0.5 µM KRAS WT in the presence of 0.5 µM BODIPY-GTP. SOS1-mediated nucleotide exchange was monitored as a change in fluorescence over time for 30 minutes using an EnSpire multimode plate reader (PerkinElmer, Waltham, MA, USA) with excitation at 466.5 nm and emission collected at 512-520 nm. Apparent rate constants were obtained in GraphPad Prism 11 by fitting the fluorescence traces to a one-phase association model for BODIPY-GTP loading and a one-phase decay model for BODIPY-GDP dissociation. For each assay, the apparent rate constant (k_{obs}) and half-life ($t_{1/2}$) values obtained for test compounds were normalized to the corresponding DMSO control measured within the same experiment. Percent inhibition relative to DMSO was calculated as 100×[1-(k_{obs}_compound/ k_{obs}_DMSO)]. Mean values and standard deviations were calculated from four replicate measurements

3.7 Surface Plasmon Resonance

Surface plasmon resonance (SPR) experiments were performed at 25 °C using a Sierra SPR®-24 Pro instrument and the protein was immobilized on a High-Capacity Amine Sensors (Bruker Daltonics, part no. 1862614). Recombinant SOS1 was diluted at 30 µg/mL in 10 mM sodium acetate buffer, pH 5.0, containing 0.05% Tween-20. The sensor surface was activated using 400 mM 1-ethyl-3-(3-dimethylaminopropyl)carbodiimide hydrochloride (EDC) and 50 mM N-hydroxysuccinimide (NHS), supplied in the Amine Coupling Kit I (Bruker Daltonics, part no. 1862634). The EDC/NHS activation solution was injected at 10 µL/min, followed by injection of SOS1 over the flow cells B and C at the same flowrate with a contact time of 300 s, yielding an immobilization level of approximately 18,000 response units (RU). Remaining activated esters were blocked by injection of 1 M ethanolamine-HCl, pH 8.5, at 10 µL/min for 420 s, followed by a dissociation time of 60 s. Flow cell A was left unmodified and used as a reference surface. Binding experiments were performed in 10 mM HEPES, 150 mM NaCl, pH 7.4, supplemented with 0.05% Tween-20 and 1% (v/v) DMSO. DMSO calibration solutions containing 0.6%, 0.8%, 1.0%, 1.2%, and 1.4% DMSO were used to compensate for DMSO-dependent bulk refractive-index effects. Compound binding to immobilized SOS1 was monitored on both immobilized flow cells at 30 µL/min, using a contact time of 120 s and a dissociation time of 300 s. Four injections of running buffer containing 1% DMSO were applied between analyte injections to regenerate the detection spots. Data processing and kinetic analysis were performed using Sierra SPR-24 Pro Analyzer software (v3.6.28.5; Bruker Daltonics GmbH & Co. KG). Reference subtraction was performed using the unmodified flow cell A. Sensorgrams obtained at multiple analyte concentrations were globally fitted using a 1:1 Langmuir binding model to estimate apparent association (k_{on}) and dissociation (k_{off}) rate constants, from which apparent equilibrium dissociation constants (K_D) were calculated as k_{off}/k_{on}.

4. COMPUTATIONAL CHEMISTRY

4.1 Evaluation of Ligand Binding Cooperativity of the Co-crystal Structures

The analysis of binding cooperativity was conducted using Scorpion, an empirical scoring method based on localized interaction network descriptors that capture cooperative effects in protein-ligand interactions.[17] The .pdb files of SOS1 bound either to **6b** or **6f** were loaded in the VSC server using the standard view separated by interaction type, with a binding-site definition of 10 Å. Pictures were rendered with PyMOL (The PyMOL Molecular Graphics System, Version 3.1.4.1.).

5. REFERENCES

(1) Neochoritis, C. G.; Zarganes-Tzitzikas, T.; Stotani, S.; Dömling, A.; Herdtweck, E.; Khoury, K.; Dömling, A. Leuckart–Wallach Route Toward Isocyanides and Some Applications. *ACS Comb. Sci.* **2015**, *17* (9), 493–499. https://doi.org/10.1021/acscombsci.5b00066.

(2) Parsons, S.; Flack, H. D.; Wagner, T. Use of Intensity Quotients and Differences in Absolute Structure Refinement. *Acta Crystallogr B Struct Sci Cryst Eng Mater* **2013**, *69* (Pt 3), 249–259. https://doi.org/10.1107/S2052519213010014.

(3) Tamgho, I.-S.; Engle, J. T.; Ziegler, C. J. The Syntheses and Structures of Bis(Alkylimino)Isoindolines. *Tetrahedron Letters* **2013**, *54* (45), 6114–6117. https://doi.org/10.1016/j.tetlet.2013.08.134.

(4) Burla, M. C.; Caliandro, R.; Carrozzini, B.; Cascarano, G. L.; Cuocci, C.; Giacovazzo, C.; Mallamo, M.; Mazzone, A.; Polidori, G. Crystal Structure Determination and Refinement via SIR2014. *J Appl Cryst* **2015**, *48* (1), 306–309. https://doi.org/10.1107/S1600576715001132.

(5) Sheldrick, G. M. Crystal Structure Refinement with SHELXL. *Acta Cryst C* **2015**, *71* (1), 3–8. https://doi.org/10.1107/S2053229614024218.

(6) Farrugia, L. J. WinGX and ORTEP for Windows: An Update. *Journal of Applied Crystallography* **2012**, *45* (4), 849–854. https://doi.org/10.1107/S0021889812029111.

(7) Nardelli, M. Parst: A System of Fortran Routines for Calculating Molecular Structure Parameters from Results of Crystal Structure Analyses. *Computers & Chemistry* **1983**, *7* (3), 95–98. https://doi.org/10.1016/0097-8485(83)85001-3.

(8) Macrae, C. F.; Sovago, I.; Cottrell, S. J.; Galek, P. T. A.; McCabe, P.; Pidcock, E.; Platings, M.; Shields, G. P.; Stevens, J. S.; Towler, M.; Wood, P. A. Mercury 4.0: From Visualization to Analysis, Design and Prediction. *J Appl Crystallogr* **2020**, *53* (Pt 1), 226–235. https://doi.org/10.1107/S1600576719014092.

(9) Spackman, P. R.; Turner, M. J.; McKinnon, J. J.; Wolff, S. K.; Grimwood, D. J.; Jayatilaka, D.; Spackman, M. A. CrystalExplorer: A Program for Hirshfeld Surface Analysis, Visualization and Quantitative Analysis of Molecular Crystals. *J Appl Cryst* **2021**, *54* (3), 1006–1011. https://doi.org/10.1107/S1600576721002910.

(10) Jelsch, C.; Ejsmont, K.; Huder, L. The Enrichment Ratio of Atomic Contacts in Crystals, an Indicator Derived from the Hirshfeld Surface Analysis. *IUCrJ* **2014**, *1* (Pt 2), 119–128. https://doi.org/10.1107/S2052252514003327.

(11) Goebel, L.; Kirschner, T.; Koska, S.; Rai, A.; Janning, P.; Maffini, S.; Vatheuer, H.; Czodrowski, P.; Goody, R. S.; Müller, M. P.; Rauh, D. Targeting Oncogenic KRasG13C with Nucleotide-Based Covalent Inhibitors. *Elife* **2023**, *12*, e82184. https://doi.org/10.7554/eLife.82184.

(12) Kabsch, W. XDS. *Acta Cryst D* **2010**, *66* (2), 125–132. https://doi.org/10.1107/S0907444909047337.

(13) Evans, P. R.; Murshudov, G. N. How Good Are My Data and What Is the Resolution? *Acta Cryst D* **2013**, *69* (7), 1204–1214. https://doi.org/10.1107/S0907444913000061.

(14) Skubák, P.; Murshudov, G. N.; Pannu, N. S. Direct Incorporation of Experimental Phase Information in Model Refinement. *Acta Cryst D* **2004**, *60* (12), 2196–2201. https://doi.org/10.1107/S0907444904019079.

(15) Emsley, P.; Cowtan, K. Coot: Model-Building Tools for Molecular Graphics. *Acta Cryst D* **2004**, *60* (12), 2126–2132. https://doi.org/10.1107/S0907444904019158.

(16) Burns, M. C.; Sun, Q.; Daniels, R. N.; Camper, D.; Kennedy, J. P.; Phan, J.; Olejniczak, E. T.; Lee, T.; Waterson, A. G.; Rossanese, O. W.; Fesik, S. W. Approach for Targeting Ras with Small Molecules That Activate SOS-Mediated Nucleotide Exchange. *Proc Natl Acad Sci U S A* **2014**, *111* (9), 3401–3406. https://doi.org/10.1073/pnas.1315798111.

(17) Kuhn, B.; Fuchs, J. E.; Reutlinger, M.; Stahl, M.; Taylor, N. R. Rationalizing Tight Ligand Binding through Cooperative Interaction Networks. *J Chem Inf Model* **2011**, *51* (12), 3180–3198. https://doi.org/10.1021/ci200319e.
